# Antimicrobial triazinedione inhibitors of the translocase MraY–protein E interaction site: synergistic effects with bacitracin imply a new mechanism of action†

**DOI:** 10.1039/d4md00937a

**Published:** 2025-01-30

**Authors:** Julia A. Fairbairn, Rachel V. Kerr, Nika-Kare A. Pierre-White, Anthony Jacovides, Becca W. A. Baileeves, Phillip J. Stansfeld, Gerhard Bringmann, Andrew T. Merritt, Timothy D. H. Bugg

**Affiliations:** a Department of Chemistry, University of Warwick Coventry CV4 7AL UK T.D.Bugg@warwick.ac.uk; b School of Life Sciences, University of Warwick Coventry CV4 7AL UK; c Institute of Organic Chemistry, University of Würzburg Würzburg Germany; d LifeArc, SBC Open Innovation Campus Stevenage Herts SG1 2FX UK

## Abstract

*Escherichia coli* translocase MraY is the target for bacteriolytic protein E from bacteriophage ϕX174, interacting at a site close to Phe-288 on helix 9, on the extracellular face of the protein. A peptide motif Arg-Trp-x-x-Trp from protein E was used to design a set of triazinedione peptidomimetics, which inhibit particulate MraY (6d IC_50_ 48 μM), and show antimicrobial activity against Gram-negative and Gram-positive antibiotic-resistant clinical strains (7j MIC *Acinetobacter baumannii* 16 μg mL^−1^, *Staphyloccoccus aureus* MRSA 2–4 μg mL^−1^). Docking against a predicted structure for *E. coli* MraY revealed two possible binding sites close to helix 9, the binding site for protein E. Antimicrobial activity of analogue 6j was found to be synergistic with bacitracin in *Micrococcus flavus*, consistent with a link between this inhibition site and undecaprenyl phosphate uptake. Alkaloid michellamine B, also predicted to bind in the cleft adjacent to helix 9, was also found to be synergistic with bacitracin. These data provide experimental evidence that the unusual hydrophobic cleft adjacent to helix 9 in MraY is involved in uptake of undecaprenyl phosphate, in addition to recently identified transporters UptA and PopT, and that this process can be targeted by small molecules as a novel antibacterial mechanism.

## Abstract 2

A set of triazinedione peptidomimetics of an Arg-Trp-x-x-Trp motif found in MraY were found to show antimicrobial activity against antibiotic-resistant clinical isolates, *via* a novel mechanism of action.

## Graphical abstract

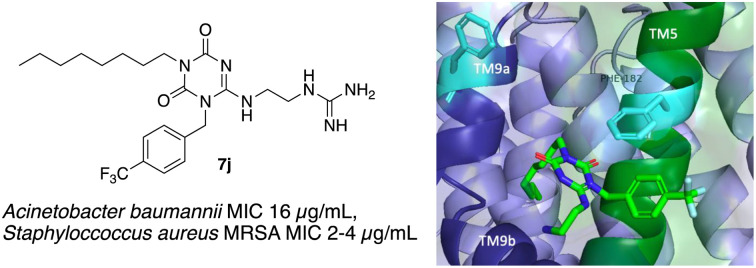

## Introduction

**Fig. 1:**
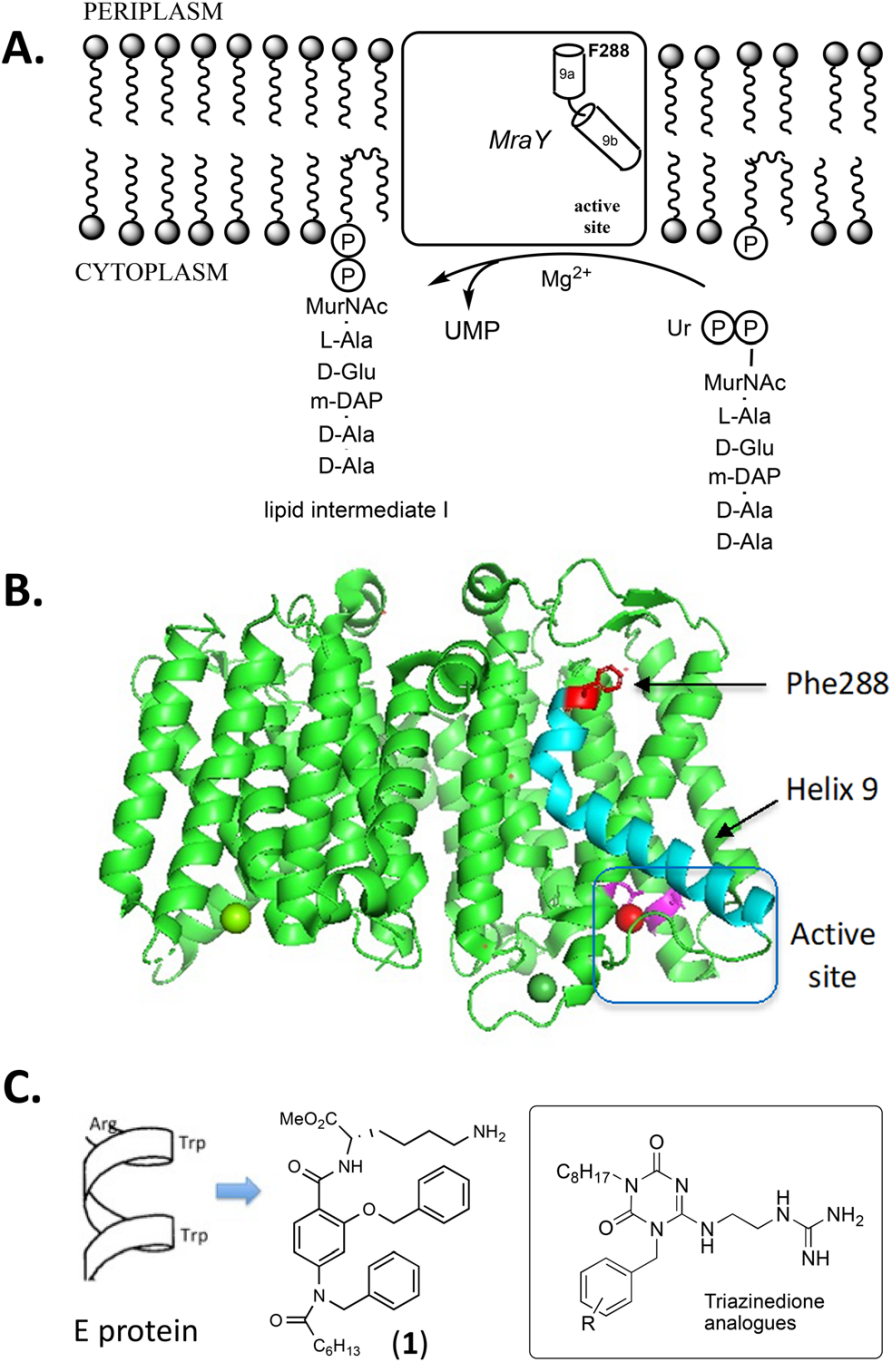
A. Reaction catalysed by translocase MraY. B. Structure of *Aquifex aeolicus* MraY, showing location of Phe-288 and enzyme active site. C. Structure of α-helical peptidomimetic 1 and triazinedione analogues described herein.

Phospho-MurNAc-pentapeptide translocase (MraY) catalyses the first reaction of the lipd-linked steps of bacterial peptidoglycan, namely the reaction of UDPMurNAc-l-Ala-γ-d-Glu-m-DAP-d-Ala-d-Ala with undecaprenyl phosphate to form lipid intermediate I (Fig. 1A).^1,2^ MraY is a 10-transmembrane helix integral membrane protein whose crystal structure was solved in 2013 (Fig. 1B).^3,4^ MraY is the site of action of several nucleoside natural product antibiotics such as the mureidomycins,^5,6^ pacidamycins,^7,8^ caprazamycins,^9^ and muraymycins,^10^ but despite a great deal of research effort into structure–function studies on these compounds,^11^ no derivative has yet been approved for clinical use.

MraY is also the site of action of the antibacterial lysis protein E from bacteriophage ϕX174.^12,13^ Protein E is a 91-amino acid protein that mediates host cell lysis in *Escherichia coli*, recruiting a SlyD helper protein.^12^ Genetic studies revealed several mutations in the *mraY* gene that conferred resistance to an Epos mutant not dependent upon SlyD, notably mutation F288L which was observed twice in the original study.^12^ Phe-288 is located on the extracellular face of helix 9, a bent α-helix that protrudes into the lipid bilayer (Fig. 1B).^3^ Recent cryo-EM studies have shown that protein E, which is also bent due to conserved Pro-19 in the transmembrane helix, binds in the hydrophobic groove adjacent to helix 9.^14^

Since the hydrophobic groove adjacent to Phe-288 is located on the extracellular face of the membrane, it is a potential site for antibacterial action that could be more easily targetted from the exterior, and our group has attempted to design small molecule agents that could target this site. Potent inhibition of particulate MraY was demonstrated by a 37-amino acid peptide encoding the transmembrane domain of protein E.^15^ A hypothesis was devised for recognition of Phe-288 or MraY by an Arg-Trp-x-x-Trp motif found in protein E, and inhibition of MraY was observed by synthetic pentapeptides based on this sequence motif, whereas no inhibition was observed for the recombinant F288L mutant MraY.^16^ Furthermore, a dipeptide derivative Arg-Trp-octyl ester was found to show antimicrobial activity against *Escherichia coli* K12 (MIC 31 μg mL^−1^) and *Pseudomonas putida* mt-2 (MIC 31 μg mL^−1^), and overexpression of *mraY* was found to substantially raise the observed MIC, consistent with an interaction with MraY.^16^ Peptidomimetic analogues mimicking the α-helical structure of the Arg-Trp-x-x-Trp motif were designed, resulting in mimetic 1 (see Fig. 1C) which showed both MraY inhibition (IC_50_ 140 μg mL^−1^) and antimicrobial activity against *Escherichia coli* K12 (MIC 7 μg mL^−1^) and *Pseudomonas fluorescens* Pf-5 (MIC 46 μg mL^−1^).^17^ Testing against clinical antibiotic-resistant ESKAPE pathogens revealed that this analogue was not active against Gram-negative ESKAPE pathogens, but did show activity against *Staphylococcus aureus* MRSA (MIC 16 μg mL^−1^) and *Enterococcus faecalis* (MIC 16 μg mL^−1^).^17^

In view of the low activity of α-helical peptidomimetics against Gram-negative ESKAPE pathogens, we have sought an alternative scaffold with more drug-like properties on which to position the three functional groups found in Arg-Trp-octyl ester,^16^ namely a guanidine or amine sidechain, an aromatic moiety, and an alkyl chain that could localise the compound in the membrane. Congiu *et al.* have reported a triazinedione scaffold which can position three substituents including a guanidine sidechain.^18^ Here we report a series of peptidomimetic analogues based on the triazinedione scaffold of Congiu *et al.* (general structure shown in Fig. 1C) which show improved antimicrobial activity, including activity against Gram-negative antibiotic-resistant clinical strains, and studies to investigate their mechanism of antimicrobial action.

## Results

### Synthesis of triazinedione analogues

**Fig. 2:**
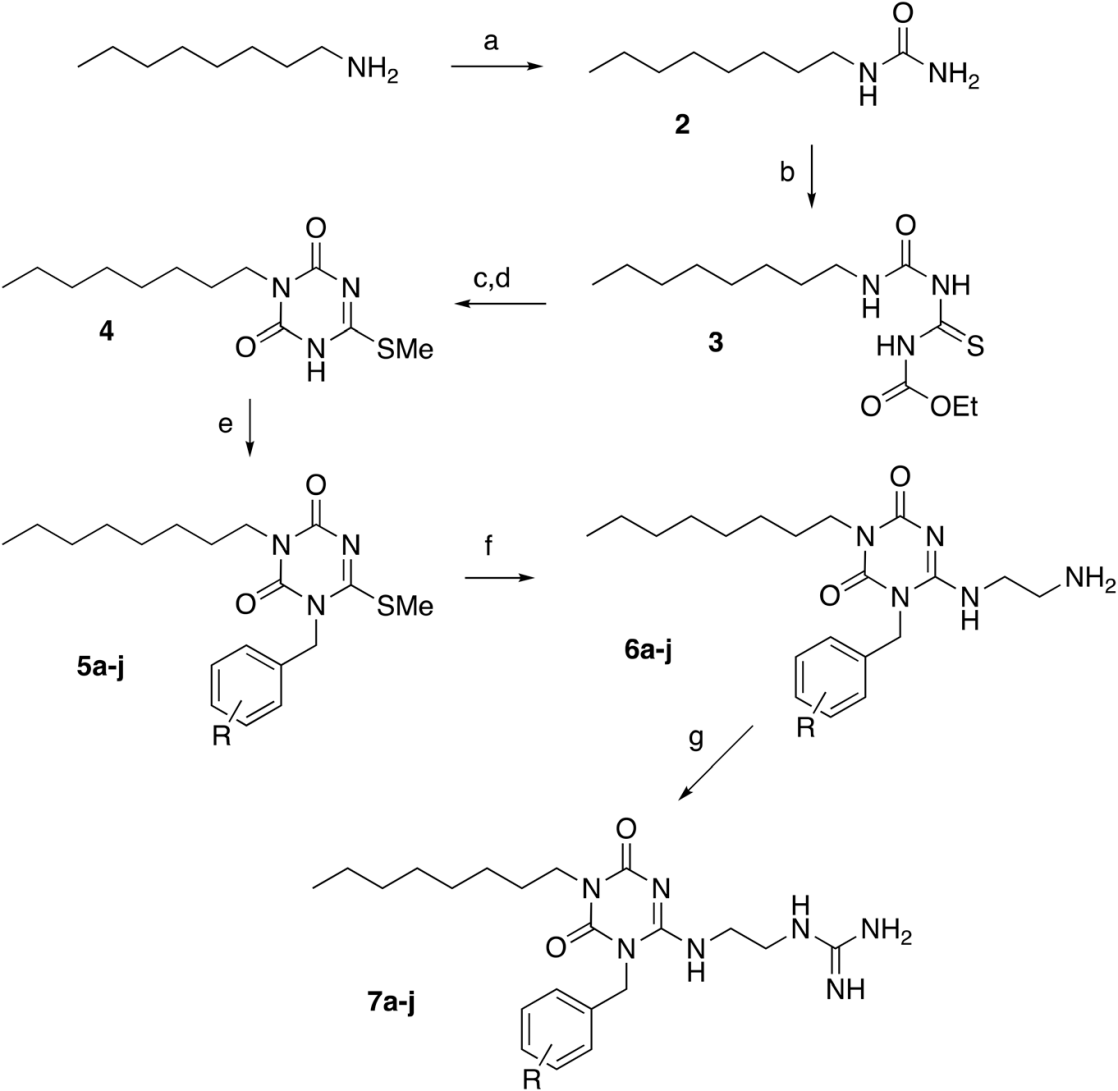
Synthetic scheme for triazinedione peptidomimetics. Reaction conditions & yields: a, KOCN, H_2_O, 60 °C, 90%; b, EtOCONCS, toluene, Δ, 72%; c, NaOMe, MeOH; d, MeI, 88%; e, K_2_CO_3_, ArCH_2_Br, 40–70%; f, ethylenediamine, 43–75%; g, 1*H*-pyrazole-1-carboxamidine, DIPEA. R substituents: a, H; b, 4-OCH_3_; c, 3-OCH_3_; d, 2-OCH_3_; e, 4-F; f, 4-Cl; g, 4-Br; h, 4-NO_2_; j, 4-CF_3_.

A set of 18 triazinedione analogues was synthesised using the synthetic route shown in Fig. 2. Analogues containing a range of aromatic substituents were synthesised, in order to examine the effect of insertion of an electron-donating group (4-OMe, 3-OMe, 2-OMe), electron-withdrawing group (4-NO_2_, 4-CF_3_), or halogen substituent (4-F, 4-Cl, 4-Br). *n*-Octylamine was converted to its corresponding urea (2), using potassium cyanate under acidic conditions, in 90% yield. Urea 2 was reacted with ethoxycarbonyl isothiocyanate at 110 °C for 6 hours, to give thiourea 3 in 72% yield. Cyclisation of 3 was achieved using anhydrous sodium methoxide in methanol, then alkylation using methyl iodide, followed by recrystallisation, to give cyclic thioimidate 4 in 88% yield. 4 was alkylated with a range of substituted benzyl bromides or chlorides using potassium carbonate as base, to give benzylated triazinediones 5a–j, typically in 40–70% yield. Reaction with ethylene diamine then proceeded in 43–75% yield to give a series of primary amine analogues 6a–j. Synthesis of the corresponding guanidines 7a–j was achieved using 1*H*-pyrazole-1-carboxamidine.

**Fig. 3:**
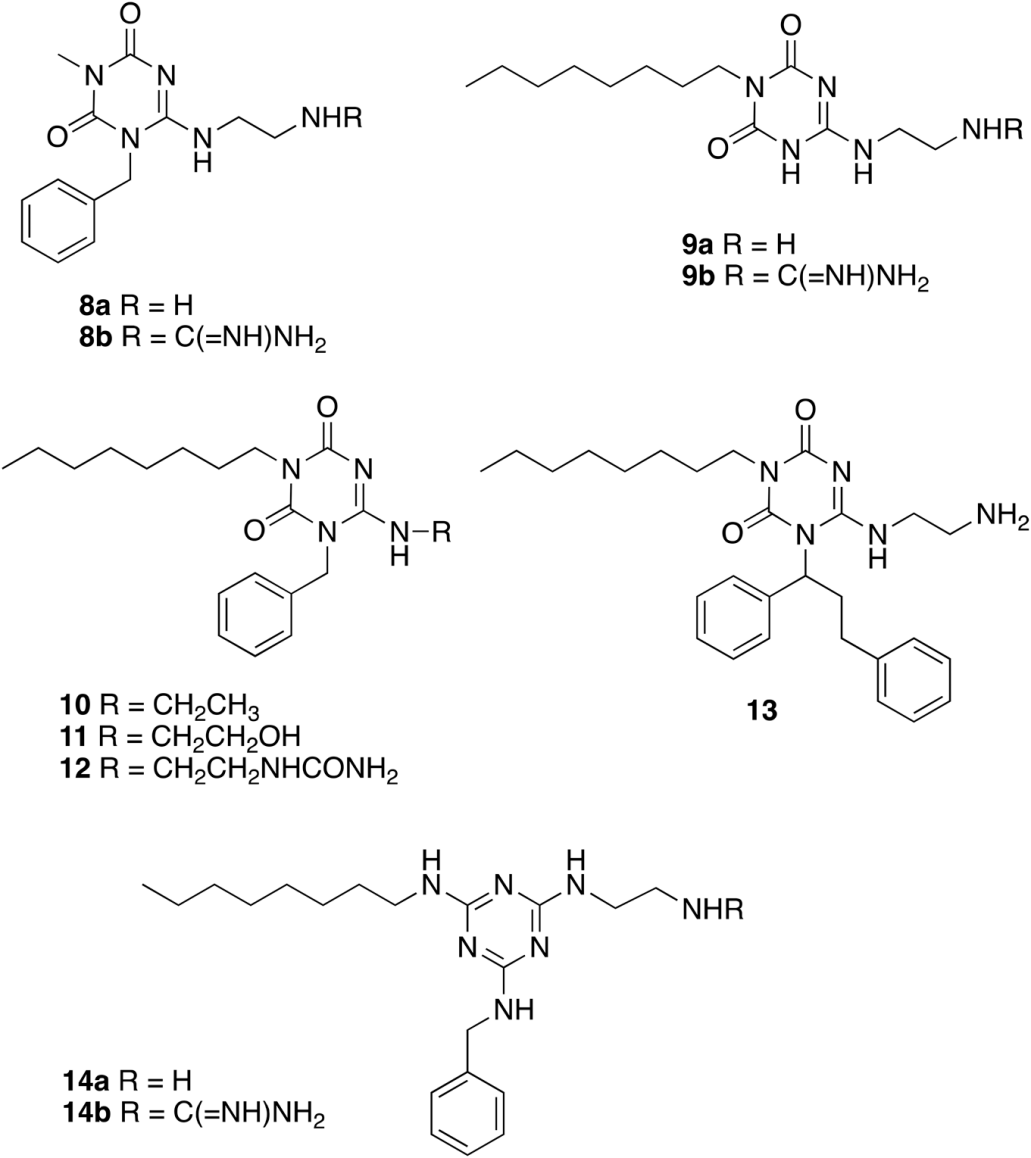
Analogues lacking key functional groups or additional groups.

Several analogues lacking key functional groups were also synthesised (see Fig. 3), using the same synthetic route, to examine the importance of each functional group. Starting from commercially available 1-methylurea, amine 8a and guanidine 8b containing a methyl group in place of the *n*-octyl substituent were prepared. Starting from synthetic intermediate 4, amine 9a and guanidine 9b were prepared, lacking the aromatic group. Starting from synthetic intermediate 5a, analogue 10 was prepared, containing an *N*-ethyl sidechain in place of a basic sidechain, and analogue 11 containing an alcohol group in place of an amine. Analogue 12 containing an uncharged urea sidechain in place of a guanidine was prepared from intermediate 6a.

Since the Arg-Trp-x-x-Trp contains two aromatic groups, and the most active α-helical peptidomimetic 1 prepared by Kerr *et al.* also contained two aromatic sidechains,^17^ analogue 13 was also prepared, containing a sidechain with two aromatic groups, *via* alkylation with the corresponding bis-aryl chloride. A further pair of analogues 14a (amine) and 14b (guanidine) containing the same substituents but on a slightly different triazine-1,3,5-triamine scaffold was also synthesised, from 1,3,5-trichlorotriazine.

### Antimicrobial testing

The set of analogues were tested for antimicrobial activity against laboratory strains of *E. coli* K12, *B. subtilis* W23, and *Pseudomonas fluorescens* Pf-5. As shown in Table 1, the compounds showed antimicrobial activity, comparable or better than the earlier α-helical peptidomimetic.^17^ The compounds containing the guanidine sidechain (7a–j) showed improved antimicrobial activity, compared with those containing the amine sidechain (6a–j). Best activity was observed for compound 7j containing the 4-CF_3_ aryl substituent, which showed MIC values of 4 μg mL^−1^ against *E. coli* K12, and 1 μg mL^−1^ against *B. subtilis* W23. Compounds lacking the *n*-octyl chain (8ab) or aromatic ring (9a) or amine/guanidine sidechain (10, 11) showed no antimicrobial activity, demonstrating the importance of each of these groups for antimicrobial activity. Compound 12 containing a urea sidechain in place of a guanidine showed weak antimicrobial activity (*E. coli* MIC 64 μg mL^−1^), indicating that the charged guanidinium sidechain was beneficial but not essential for antimicrobial action. Compound 13 containing the bis-aryl substituent showed poor antimicrobial activity. Compounds 14a and 14b based on a triazine-triamine scaffold showed 4-fold reduced antimicrobial activity (*E. coli* MIC 64 μg mL^−1^), compared with compounds 6a/7a, hence this scaffold was not investigated further.

**Table 1:** Antimicrobial activity (MIC_50_) of triazinedione analogues against laboratory strains, and % inhibition of overexpressed MraY @200 μM concentration, by continuous fluorescence assay

Cpd	Substituent	Sidechain	*Escherichia coli* K12 (μg mL^−1^)	*Bacillus subtilis* W23 (μg mL^−1^)	*Pseudomonas fluorescens* Pf-5 (μg mL^−1^)	% MraY inhibition @200 μM
6a	H	Amine	16	8	64	36
6b	4-OCH_3_	Amine	16	8	32	50
6c	3-OCH_3_	Amine	64	32	>256	42
6d	2-OCH_3_	Amine	64	32	>256	80
6e	4-F	Amine	64	32	128	35
6f	4-Cl	Amine	8	4	64	15
6g	4-Br	Amine	16	16	64	68
6h	4-NO_2_	Amine	64	32	>256	63
6j	4-CF_3_	Amine	64	32	>256	59
7a	H	Guanidine	16	8	32	41
7b	4-OCH_3_	Guanidine	16	2	32	48
7c	3-OCH_3_	Guanidine	16	4	32	57
7d	2-OCH_3_	Guanidine	16	4	>256	57
7e	4-F	Guanidine	16	4	32	63
7f	4-Cl	Guanidine	8	8	32	42
7g	4-Br	Guanidine	16	4	256	51
7h	4-NO_2_	Guanidine	64	32	256	70
7j	4-CF_3_	Guanidine	4	1	16	43
8a	Alkyl C_1_	Amine	>256	>256	NT	6.5
8b	Alkyl C_1_	Guanidine	>256	>256	NT	42
9a	No aryl	Amine	>256	>256	NT	38
10	H	*N*-Ethyl	>256	>256	NT	36
11	H	Hydroxyl	>256	>256	NT	39
12	H	Urea	64	128	NT	39
13	Bis-aryl	Amine	256	128	NT	64
14a	Triazine	Amine	64	NT	NT	23^a^
14b	Triazine	Guanidine	64	NT	NT	24^a^

a Measured by radiochemical assay.

A group of 8 analogues which showed higher antimicrobial activity was further tested against a panel of 6 Gram-negative and Gram-positive antibiotic-resistant clinical strains (see Table 2). Best activity was observed for compound 7j containing the 4-CF_3_ aryl substituent, against *Enterobacter cloacae* 19434 (MIC 16 μg mL^−1^), *Klebsiella pneumoniae* 700603 (MIC 16 μg mL^−1^), *Acinetobacter baumannii* 19606 (MIC 16 μg mL^−1^), *Pseudomonas aeruginosa* NCTC 13437 (MIC 32 μg mL^−1^), *Staphylococcus aureus* MRSA USA300 JE2 (MIC 2–4 μg mL^−1^), and *Enterococcus faecium* 19434 (MIC 8–16 μg mL^−1^). Guanidine-containing analogues 7a and 7b also showed effective antimicrobial activity against the panel of strains, with best activity against *S. aureus* MRSA (MIC 8 μg mL^−1^).

**Table 2:** Antimicrobial activity (MIC_50_) of triazinedione analogues against antibiotic-resistant clinical strains

Cpd	R	Sidechain	*Enterobacter cloacae* 19434 (μg mL^−1^)	*Klebsiella pneumoniae* 700603 (μg mL^−1^)	*Acinetobacter baumannii* 19606 (μg mL^−1^)	*Pseudomonas aeruginosa* NCTC 13437 (μg mL^−1^)	*Staphylococcus aureus* MRSA USA300 JE2 (μg mL^−1^)	*Enterococcus faecium* 19434 (μg mL^−1^)
6a	H	Amine	16–32	64	32	32–64	16–32	32
7a	H	Guanidine	16–32	64–128	32–64	64	8	32
6b	4-OMe	Amine	16–32	32–64	32	64	16–32	32
7b	4-OMe	Guanidine	64	128	64	64–128	8–16	32
6d	2-OMe	Amine	256	>256	256	>256	128–256	>256
6j	4-CF_3_	Amine	8–16	32	16	32	8	16
7j	4-CF_3_	Guanidine	16	16	16	32	2–4	8–16
13	Bis-aryl	Amine	>256	>256	128–256	>256	128–256	256

### Activity against translocase MraY

**Fig. 4:**
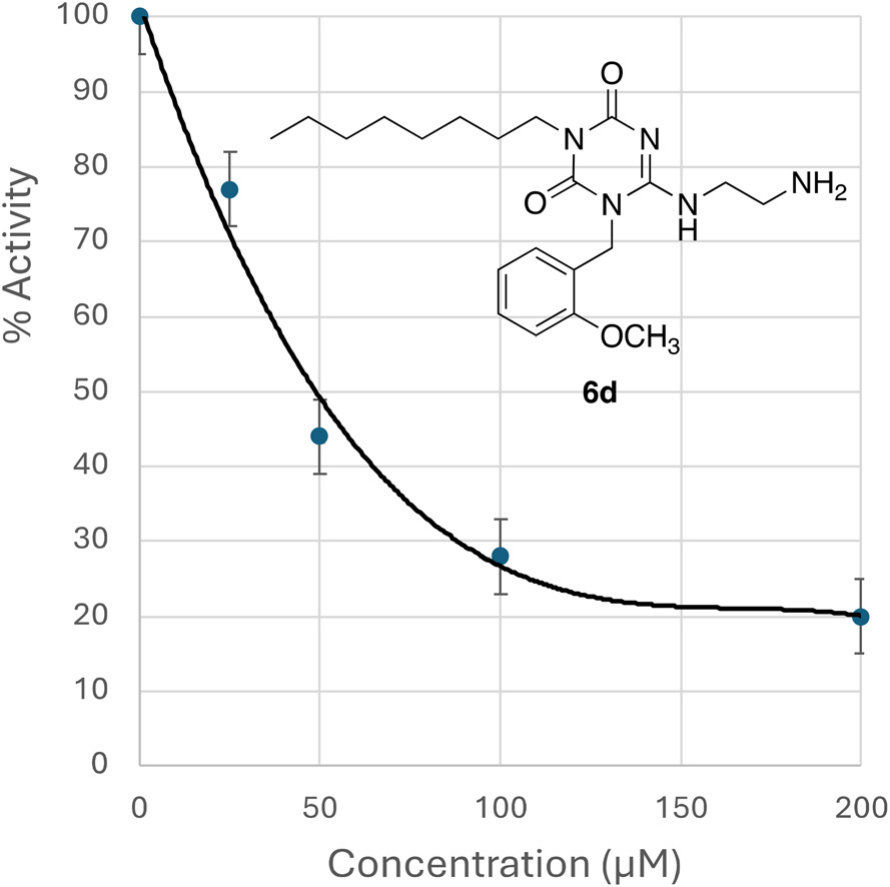
IC_50_ determination for compound 6d against overexpressed *E. coli* MraY.

The set of analogues were assayed against particulate *E. coli* MraY from overexpressed membranes, using a fluorescence enhancement assay with fluorescent substrate UDPMurNAc-l-Ala-γ-d-Glu-l-Lys(ε-*N*-dansyl)-d-Ala-d-Ala, used previously to assay MraY.^5,16,17^ Compounds 14a and 14b were assayed using a radiochemical assay used previously by Mihalyi *et al.*,^19^ since they exhibited background fluorescence that interfered with the continuous fluorescence assay. As shown in Table 1, the analogues showed 15–80% inhibition of MraY at 200 μM concentration, with highest enzyme inhibition shown by compound 6d. For this compound, an IC_50_ value of 48 μM was determined, as shown in Fig. 4. It has been observed previously using 37 amino acid peptide E_pep_ that incomplete inhibition of particulate MraY is observed even at high inhibitor concentrations, because the site of action is remote from the enzyme active site.^15^

Although the compounds showed MraY inhibition, the enzyme inhibition data did not correlate with the observed antimicrobial activity. Therefore, further experiments were carried out, to investigate in more detail the mode of action for these compounds. Previously we have overexpressed site-directed mutant R288L MraY, containing the mutation which confers resistance to protein E, and found that no inhibition of this mutant was observed by either 37-amino acid E_pep_ containing the transmembrane domain of protein E, or by synthetic pentapeptides based on the Arg-Trp-x-x-Trp motif.^16^ Compounds 6a–j and 7a–j were assayed against overexpressed mutant R288L MraY, and no inhibition was observed at 200 μM concentrtion, consistent with the compounds targeting this region of the protein. We have also observed previously that overexpression of *mraY* in *E. coli* leads to a higher observed MIC for agent Arg-Trp-octyl ester.^16^ The set of analogues were tested for antimicrobial activity against *E. coli* C43 containing a pET28a vector overexpressing *mraY*, compared with the same strain containing empty vector. Although there were some differences in MIC between *E. coli* C43 with & without the empty vector, there was no increase in MIC observed for the strain overexpressing *mraY*, compared with empty vector (data shown in ESI† Table S1).

**Fig. 5:**
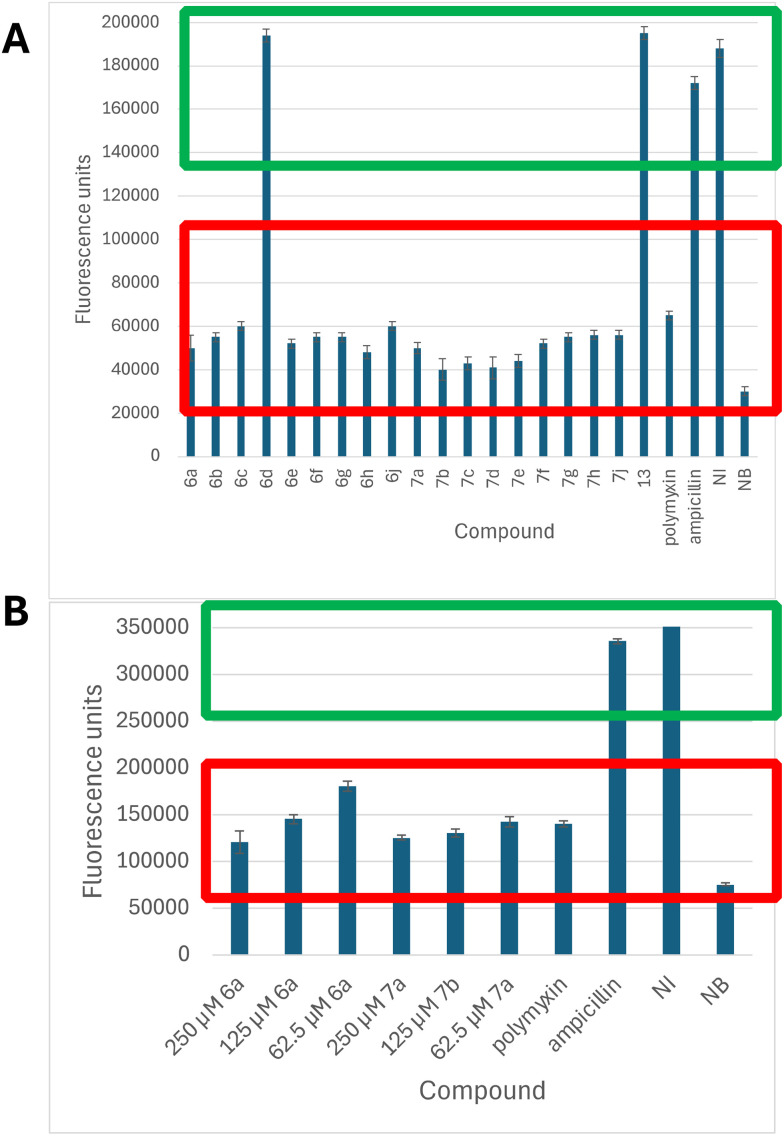
alamarBlue cell viability assays in *E. coli* Top10 cells. A. Testing of the set of analogues at 250 μg mL^−1^ concentration, compared with ampicillin and polymyxin. B. Testing of analogues 6a and 7a at 62.5, 125 and 250 μg mL^−1^ concentrations. Green box indicates bacteriostatic range, red box indicates bacteriocidal range. Key: NI, no inhibitor; NB, no bacteria.

Cell viability assays were carried out, using the alamarBlue reagent,^20^ in order to probe whether the compounds were bacteriostatic or bacteriocidal. *E. coli* Top10 cells were grown in the presence of 250 μg mL^−1^ concentration of inhibitor, and the results compared with ampicillin, a bacteriostatic agent,^21^ and polymyxin, a bacteriocidal agent.^22^ As shown in Fig. 5A, the majority of compounds appeared to be bacteriocidal at 250 μg mL^−1^, with the exception of compound 6d (R = 2-OMe, amine sidechain), and compound 13 (bis-aryl analogue). Two compounds, 6a and 7a, were tested at different concentrations, as shown in Fig. 5B, and the results showed concentration-dependent effects, with less bacteriocidal effects observed at lower concentration.

### Docking of peptidomimetics with MraY structure

**Fig. 6:**
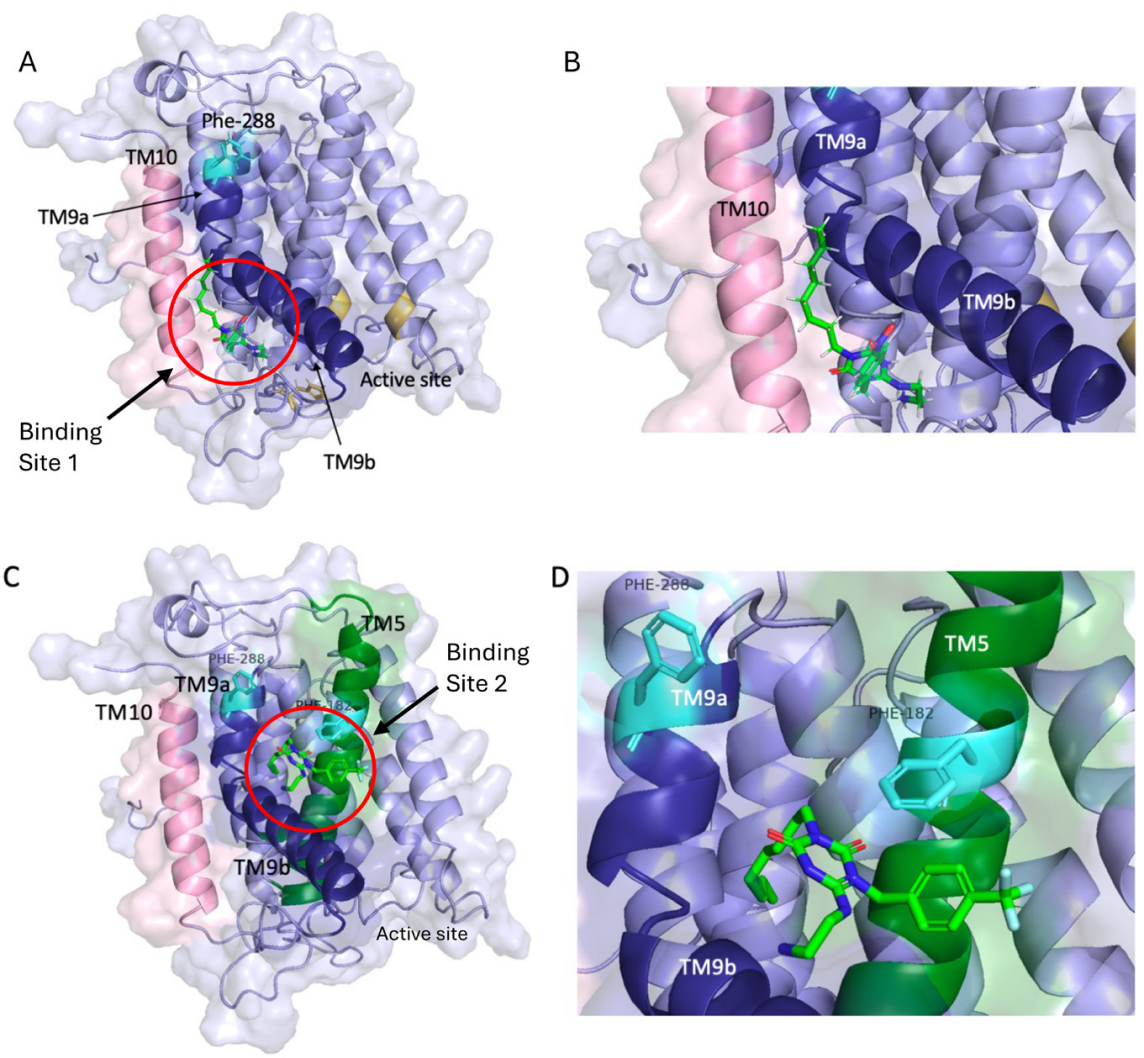
Docking of peptidomimetics to structure of *E. coli* MraY. A and B, binding site 1, complex with compound 6h. C and D, binding site 2, complex with compound 6j. The molecular interactions in these two poses are shown in ESI† Fig. S1 and S2.

In order to probe possible binding sites for the peptidomimetic structures with *E. coli* MraY, a predicted *E. coli* MraY structure was generated using AlphaFold2 software,^23^ which matched the experimentally determined *A. aeolicus* MraY structure.^3^ The set of peptidomimetic structures was docked with this MraY structure using Schrodinger Maestro software, which generated the top 50 poses for the library of compounds as a set. The compounds were observed to dock at two possible interaction sites, shown in Fig. 6, both close to helix 9 in the structure, but not immediately adjacent to Phe-288. Site 1 (observed for 27 out of 50 poses, Fig. 6A and B) is located between helix 9b and helix 10, close to the MraY active site, which is situated on the other side of helix 9b. Interactions were observed between the peptidomimetics and the sidechains of Glu-300 of helix 9b and Asp-198 of helix 5 (see ESI† Fig. S1). Compounds showing higher MraY inhibition, including compound 6d, were generally observed at site 1 (see ESI† Table S2). Site 2 (observed for 23 out of 50 poses, Fig. 6C and D) is located at the “elbow” of helix 9a and 9b, interacting with Phe-182 of helix 5. In this site, π–π interactions were observed between the aromatic sidechain of the peptidomimetics and the sidechain of Phe-182 (see ESI† Fig. S2). Compounds showing higher antimicrobial activity, including compounds 6j and 7j, were generally observed at site 2 (see ESI† Table S2).

Further docking of compound 7j to an AlphaFold2 (ref. 23) model of *E. coli* MraY using SwissDock^24^ also gave bound conformations in binding site 2 shown above (see ESI† Fig. S3). Docking of dipeptide Arg-Trp-octyl ester, identified in our earlier work,^16^ using SwissDock also gave bound conformations in binding site 2, between helix 9 and helix 5, which are shown in ESI† Fig. S4.

The observed interaction of ligands in binding site 2 with Phe-182 in helix 5 suggested that there might be interactions of protein E and peptidomimetics thereof to Phe-182, which is conserved as Phe in >80% of Gram-negative MraY sequences (see ESI† Fig. S5). Examination of protein E sequences revealed a conserved Phe-12 residue in the transmembrane domain of E (see ESI† Fig. S6), which we hypothesised could potentially interact with Phe-182 of MraY. This hypothesis was tested by synthesis and assay of decapeptide RWLLWLLLLF, longer than RW-containing pentapeptides tested previously by Rodolis *et al.*^16^ At 200 μM concentration, 35% inhibition of *E. coli* MraY was observed by this decapeptide. However, when compared with IC_50_ values measured previously for pentapeptides RWGGW (IC_50_ 209 μM) and RWGLW (IC_50_ 590 μM),^16^ there was no significant improvement in binding, hence we could not obtain experimental evidence for a specific interaction of Phe-12 of protein E.

### Antimicrobial activity is synergistic with bacitracin

The lack of a clear correlation between antimicrobial activity and MraY inhibition suggests that killing action of these peptidomimetics is not *via* inhibition of the MraY active site. We considered another hypothesis for the mechanism of antimicrobial action. Two transporters for undecaprenyl phosphate, the lipid substrate for MraY, have recently been identified in *B. subtilis*: UptA from the DedA superfamily, and PopT containing domain DUF368.^25,26^ We noted that the double *B. subtilis* mutant lacking both UptA and PopT was still viable, implying that there is another cellular transporter for undecaprenyl phosphate.^25^ Rodolis *et al.* have previously proposed that MraY may assist in the uptake of uridyl peptide antibiotics to the MraY active site, *via* the hydrophobic channel adjacent to helix 9.^27^ It seemed possible therefore that the hydrophobic channel adjacent to helix 9 may be an additional channel for uptake of undecaprenyl phosphate, and that blocking this process might reduce the supply of undecaprenyl phosphate sufficiently to cause cell death.

**Fig. 7:**
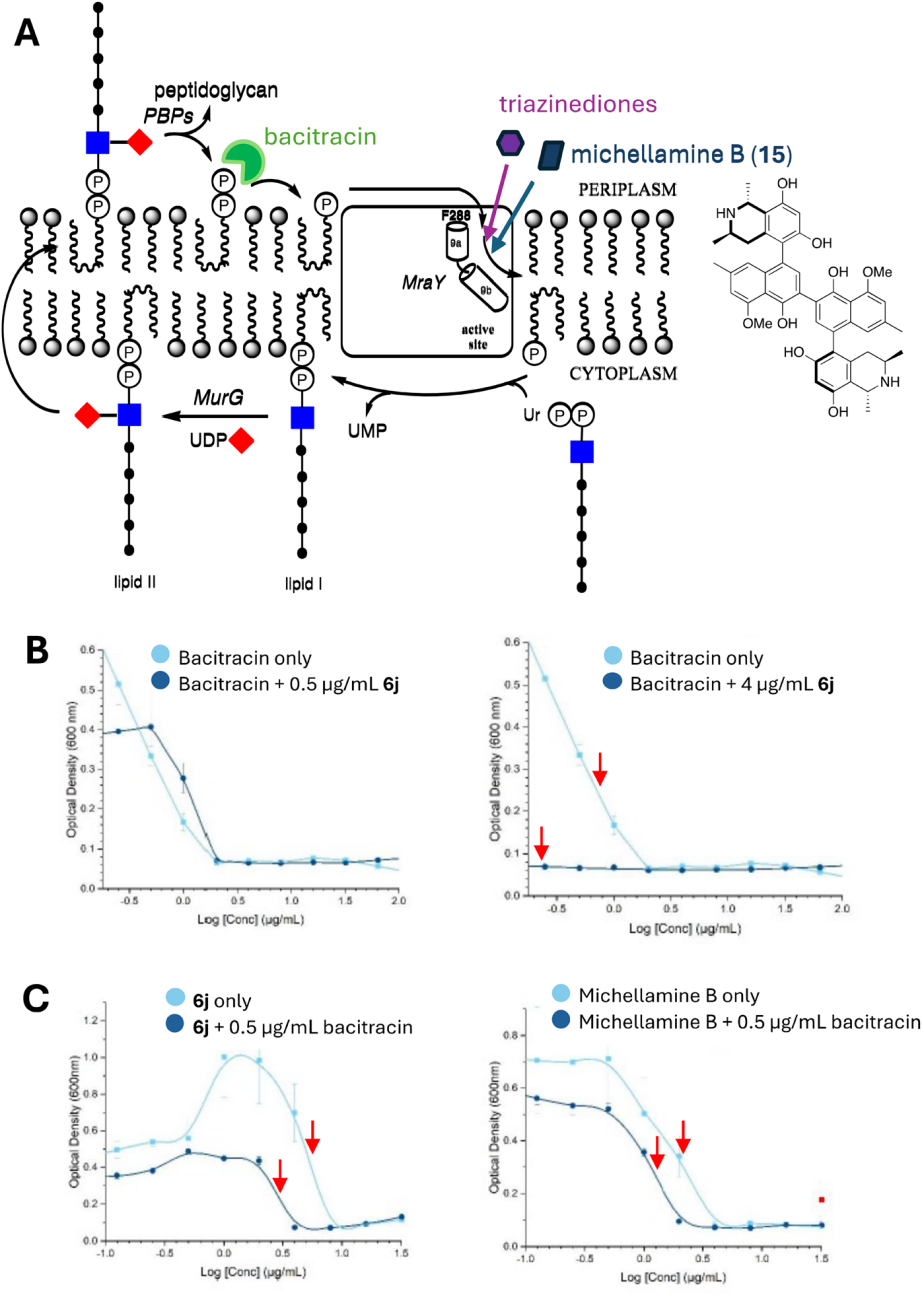
Synergistic effects between bacitracin and triazinedione 6j and michellamine B. A. Hypothesis for uptake of undecaprenyl phosphate by MraY, showing sites of action of bacitracin, triazinedione 6j, and michellamine B (chemical structure shown). B. Growth inhibition of *Micrococcus flavus* by bacitracin, showing reduction in MIC_50_ (red arrows) in the presence of 4 μg mL^−1^6j. C. Growth inhibition of *M. flavus* by 6j (left panel) and michellamine B (right panel), showing reduction in MIC_50_ (red arrows) in the presence of 0.5 μg mL^−1^ bacitracin. Growth experiments carried out with biological triplicates, error bars show standard deviation.

Given that *mraY* is an essential bacterial gene,^28^ it is not possible to investigate this hypothesis *via* gene knockout. However, blocking of undecaprenyl phosphate uptake might be synergistic with other agents that can reduce availability of undecaprenyl phosphate, such as bacitracin, which binds undecaprenyl pyrophosphate on the outer face of the cytoplasmic membrane,^29^ as illustrated in Fig. 7A. Although bacitracin has no antimicrobial activity against Gram-negative bacteria, since it is unable to penetrate the outer membrane of Gram-negative bacteria, we found that both bacitracin and peptidomimetic 6j are active against *Micrococcus flavus*. Bacitracin MIC against *M. flavus* was found to be 2 μg mL^−1^, while MIC for 6j was 8 μg mL^−1^. In the presence of 4 μg mL^−1^6j, MIC for bacitracin was reduced from 2 μg mL^−1^ to 0.25 μg mL^−1^, whereas this effect was not observed in the presence of 0.5 μg mL^−1^6j (see Fig. 7B). Moreover, MIC for 6j was reduced from 8 μg mL^−1^ to 2 μg mL^−1^ in the presence of 1 μg mL^−1^ bacitracin (see Fig. 7C). Hence, compound 6j is shown to be synergistic with bacitracin.

We also tested another ligand found previously from library screening to be an MraY inhibitor, naphthoisoquinoline alkaloid michellamine B (15), which from docking studies had been predicted to bind in the hydrophobic cleft adjacent to helix 9 (see ESI† Fig. S7).^19^ Michellamine B showed an MIC of 4 μg mL^−1^ against *M. flavus*, which was reduced to 2 μg mL^−1^ in the presence of 0.5 μg mL^−1^ bacitracin (see Fig. 7C). Hence, synergistic effects are also observed for a structurally unrelated agent that binds to the same site in MraY.

## Discussion

Compared with our previous series of α-helical peptidomimetics based on the Arg-Trp-x-x-Trp motif,^17^ the triazinedione series of peptidomimetics reported here show improved antimicrobial activity, especially against clinical antibiotic-resistant strains (see Table 2). The most active compound, against both laboratory strains and clinical strains, is guanidine analogue 7j containing a 4-CF_3_ aryl substituent, which shows good activity against Gram-negative *Enterobacter cloacae* 19 434 (MIC 16 μg mL^−1^), *Klebsiella pneumoniae* 700 603 (MIC 16 μg mL^−1^), and *Acinetobacter baumannii* 19 606 (MIC 16 μg mL^−1^), and is very active against Gram-positive *Staphylococcus aureus* MRSA USA300 JE2 (MIC 2–4 μg mL^−1^), and *Enterococcus faecium* 19 434 (MIC 8–16 μg mL^−1^). There is consistent antimicrobial activity across the series of compounds, and across multiple bacterial strains, suggesting that this is an effective antimicrobial target site, which can be rationalised by Phe-288 being on the periplasmic face of MraY (see Fig. 1B), allowing better access to this site by external agents.

Although these analogues do show inhibition of particulate MraY enzyme activity, there is surprisingly no clear correlation between antimicrobial activity & MraY inhibition activity (see Tables 1 and 2). This observation implies that the killing action of this series of compounds is not *via* inhibition at the MraY active site, and raises the possibility of either second protein target, or the existence of another binding site within MraY. It is clear from the MraY structure that Phe-288, which is linked to the action of protein E,^12^ is remote from the enzyme active site (see Fig. 1B). Furthermore, peptide E_pep_ has been previously shown not to inhibit detergent-solubilised MraY, but does inhibit particulate MraY,^15^ and we also observed no inhibition of MraY by Arg-Trp-octyl ester.^16^ Hence it is not necessarily the case that there must be a correlation between binding of ligands close to Phe-288 and MraY active site inhibition.

Therefore we have used several approaches to probe further the mechanism of action of these compounds. We have observed no inhibition of the R288L MraY mutant, as observed previously for Arg-Trp-containing pentapeptides,^16^ which is consistent with these compounds targetting a site near Phe-288. Molecular docking against a predicted structure of *E. coli* MraY suggests two possible binding sites, both near the bent helix 9, but further along the helix from Phe-288. A possible rationalisation is therefore that binding to site 1, located close to the MraY active site, has a greater effect on MraY activity in active site, which is supported by the observation of binding to site 1 by compound 6d which shows strongest MraY inhibition (see Fig. 4), but which shows weak antibacterial activity against clinical strains (see Table 2). Conversely, our hypothesis is that binding to site 2, located at the “elbow” of helix 9a and 9b in MraY, has little effect on MraY active site, but does somehow lead to antimicrobial activity, which is supported by the observation of binding to site 2 by compounds 6j and 7j which show strong antimicrobial activity against clinical strains. We cannot rule out the possibility of a second site of action for these compounds, but this hypothesis can explain the observed data.

The outstanding question is why binding to site 2 actually leads to antimicrobial action. We have previously proposed in 2006 that there may be a protein–protein interaction between MraY and an unidentified cell division protein, and that blocking this interaction may lead to cell death during cell division,^15^ which is still a possible hypothesis. However, recent reports of two protein transporters for undecaprenyl phosphate,^25,26^ which is the substrate for MraY, raised the possibility that the unusual hydrophobic cleft formed by bent helix 9 in MraY might be a further site for uptake of undecaprenyl phosphate, a hypothesis that we have previously proposed in 2014 for cellular uptake of uridyl peptide antibiotic inhibitors of MraY.^27^

In order to investigate further, we have tested whether the triazinedione compounds are synergistic with bacitracin, which sequesters undecaprenyl pyrophosphate on the cell surface.^29^ We have observed an 8-fold reduction in MIC for bacitracin in the presence of a sub-MIC dose of compound 6j, and a 4-fold reduction in MIC for compound 6j in the presence of a sub-MIC dose of bacitracin. These observations imply a connection between the site of killing action of compound 6j and undecaprenyl phosphate processing. The most likely explanation is that the hydrophobic cleft adjacent to helix 9 is involved in uptake of undecaprenyl phosphate from the exterior of membrane, directly into the MraY active site. Blocking this uptake limits the availability of undecaprenyl phosphate, which then leads to cell death. The action of bacitracin reduces the pool of undecaprenyl pyrophosphate (and hence undecaprenyl phosphate), making the microbe more susceptible to the action of 6j. Conversely, the binding of 6j will partially block the whole lipid cycle, which requires a catalytic amount of C_55_ lipid carrier,^1^ which then renders the microbe more susceptible to bacitracin. The observation of a 2-fold reduction in MIC for michellamine B, a structurally unrelated ligand that is also predicted to bind to the hydrophobic cleft adjacent to helix 9, in the presence of a sub-MIC dose of bacitracin, further supports this hypothesis. Consequently this is the first experimental evidence in favour of MraY being involved in uptake of undecaprenyl phosphate. We also note a recent report by Ichikawa and coworkers of synthetic nucleoside MraY inhibitors, in which the addition of substituent containing a C_16_ acyl group attached to an l-arginine-aryl spacer notably improved the antimicrobial properties of the compounds,^30^ which may relate to our observations in this series of compounds. Targetting uptake of undecaprenyl phosphate uptake is a novel mechanism of antimicrobial action, that appears effective against a range of Gram-negative and Gram-positive pathogens.

## Experimental section

### Materials

Chemical and biochemical reagents were purchased from Merck, Sigma Aldrich or Fisher Scientific. Undecaprenyl phosphate was purchased from Larodan Fine Chemicals. Michellamine B (15) was isolated from the Central-African liana *Ancistrocladus congolensis* (Ancistrocladaceae), following a published protocol.^31^

1-Octylurea (2) was prepared using a modification of the method of Congiu *et al.*^18^ 1-Octylamine (3.91 g, 5 mL, 3.03 × 10^−2^ mol, 1 eq.) and concentrated hydrochloric acid (3 mL, 3.60 × 10^−2^ mol, 1.2 eq.) were stirred in hot ethanol (60 °C, 20 mL) for 15 minutes. A solution of potassium cyanate (9.82 g, 1.21 × 10^−1^ mol, 4 eq.) in water (20 mL) was added, the solution was allowed to cool to room temperature, then stirred at room temperature for 48 h. The resulting white precipitate was collected *via* vacuum filtration to afford compound 2 (4.77 g, 91%). *R*_f_ 0.53 (9 : 1 EtOAc : MeOH). ^1^H NMR: (400 MHz, CD_3_OD) *δ*_H_ 6.14 (s, 2H, CONH_2_), 5.45 (s, 1H, NH), 3.08 (t, *J* = 7.0 Hz, 2H, CH_2_N), 1.47 (qui, *J* = 6.8 Hz, 2H, CH̲_2_CH_2_N), 1.38–1.19 (m, 10H), 0.90 (t, *J* = 6.6 Hz, 3H, CH_3_) ppm. ^13^C NMR: (100 MHz, CD_3_OD) *δ*_C_ 151.6, 41.0, 33.0, 31.2, 30.5, 30.4, 27.9, 23.7, 14.4 ppm. LRMS *m*/*z* (ESI): 173.1 (M + H)^+^, 195.1 (M + Na)^+^. HRMS *m*/*z* (ESI): calculated for C_9_H_20_N_2_NaO^+^ 195.1473, observed 195.1468.

1-((Octyl)aminocarbonyl)-3-(ethoxycarbonyl) thiourea (3) was prepared using a modification of the method of Congiu *et al.*^18^ Urea 2 (5.00 g, 2.90 × 10^−2^ mol, 1 eq.) and ethoxycarbonyl isothiocyanate (4.57 g, 5.48 mL, 4.64 × 10^−2^ mol, 1.6 eq.) were refluxed in toluene (60 mL) at 110 °C for 6 h. Upon completion of the reaction, as demonstrated by TLC, the toluene was removed under reduced pressure to afford a thick orange oil. This oil was resuspended in ethyl acetate (80 mL) and washed once with water (80 mL), and once with saturated sodium chloride solution (80 mL). The organic layer was collected, dried (MgSO_4_), and the solvent was removed under reduced pressure. The crude yellow oil was recrystallised in hot petroleum ether (b.p. 60–80 °C) (100 mL). The resulting yellow solid was washed with cold petroleum ether and collected *via* vacuum filtration to afford compound (3) as a yellow solid (6.31 g, 72%). *R*_f_ 0.82 (1 : 1 EtOAc : petroleum ether). ^1^H NMR: (400 MHz, d_6_-acetone) *δ*_H_ 11.52 (s, 1H, NH), 10.45 (s, 1H, NH), 9.39 (s, 1H, NH), 4.28 (q, *J* = 7.1 Hz, 2H, OCH̲_2_CH_3_), 3.33 (q, *J* = 6.5 Hz, 2H, CH_2_N), 1.58 (qui, *J* = 6.9 Hz, 2H), 1.48–1.20 (m, 13H), 0.89 (t, *J* = 5.8 Hz, 3H, CH_3_) ppm. ^13^C NMR: (100 MHz, d_6_-acetone) *δ*_C_ 180.1, 153.8, 152.9, 63.7, 47.3, 40.6, 32.5, 30.1, 29.9, 27.6, 23.3, 15.0, 14.3 ppm. LRMS *m*/*z* (ESI): 304.2 (M + H)^+^, 326.2 (M + Na)^+^. HRMS *m*/*z* (ESI): calculated for C_13_H_25_N_3_NaO_3_S^+^ 326.1509, observed 326.1509.

6-(Methylthio)-3-octyl-1,3,5-triazine-2,4(1*H*,3*H*)-dione (4) was prepared using a modification of the method of Congiu *et al.*^18^ Thiourea 3 (3.58 g, 1.18 × 10^−2^ mol, 1 eq.) and sodium methoxide solution (4.1 mL, 1.77 × 10^−2^ mol, 1.5 eq., 25% conc. in MeOH) were refluxed in anhydrous methanol (40 mL) at 65 °C for 2 hours. Upon completion, as determined by TLC, the solution was allowed to cool to room temperature. Iodomethane (2.2 mL, 3.53 × 10^−2^ mol, 3 eq.) was added dropwise and the resulting solution was stirred at room temperature for 4 h, followed by TLC. Upon completion, the methanol was removed under reduced pressure, and diethyl ether (50 mL) was added to the residue, leading to the formation of a white solid. The resulting white precipitate was collected *via* vacuum filtration and washed with ice-cold diethyl ether to afford compound 4 as a white solid (2.82 g, 88%). *R*_f_ 0.38 (1 : 1 EtOAc : petroleum ether). ^1^H NMR: (400 MHz, CD_3_OD) *δ*_H_ 8.55 (s, NH), 3.81 (t, *J* = 7.5 Hz, 2H, CH_2_N), 2.38 (s, 3H, SCH_3_), 1.60 (qui, *J* = 7.0 Hz), 1.36–1.26 (m, 10H), 0.89 (t, *J* = 6.5 Hz, 3H, CH_3_) ppm. ^13^C NMR (100 MHz, CH_3_OD) *δ*_C_ 182.3, 160.9, 149.2, 42.1, 33.0, 30.5, 30.4, 28.9, 28.1, 23.7, 14.4, 13.7 ppm. LRMS *m*/*z* (ESI): 270.1 (M–H)^−^. HRMS *m*/*z* (ESI): calculated for C_12_H_21_N_3_NaO_2_S^+^ 294.1238, observed 294.1247.

Method for preparation of substituted triazinediones 5a–j was adapted from the method of Congiu *et al.*^18^ 1-benzyl-6-(methylthio)-3-octyl-1,3,5-triazine-2,4(1*H*,3*H*)-dione (5a) Compound 4 (0.70 g, 2.58 ×10^−3^ mol, 1 eq.) and potassium carbonate (1.78 g, 1.29 × 10^−2^ mol, 5 eq.) were added to a round-bottom flask and placed under nitrogen. Anhydrous DMF (20 mL) was added, and benzyl bromide (1.10 g, 0.77 mL, 6.45 × 10^−3^ mol, 2.5 eq.) was added dropwise. The resulting solution was stirred at room temperature for 120 h and followed by TLC until completion. Work-up method A (compounds 5a, b, e, f, g). Distilled water (80 mL) was added to the solution, leading to the formation of a fine, white precipitate. The resulting white solid was collected *via* vacuum filtration. Work-up method B (compounds 5c, d, h, j). Distilled water (100 mL) was added to the flask, but no precipitate was formed. The product was extracted with ethyl acetate (2 × 50 mL). The combined organic layers were washed with saturated sodium chloride solution (80 mL), dried (MgSO_4_) and evaporated at reduced pressure to yield the crude product as an oil. The product was purified by neutral alumina column chromatography, eluting with 9 : 1 petroleum ether/ethyl acetate. Fractions shown by TLC to contain the desired product were combined and evaporated at reduced pressure to give the product as an oil.

Compound 5a was isolated *via* method A (0.418 g, 48%). *R*_f_ 0.73 (1 : 1 EtOAc : petroleum ether). ^1^H NMR: (400 MHz, d_6_-acetone) *δ*_H_: 7.40–7.29 (m, 5H), 5.19 (s, 2H, CH_2_Ar), 3.86 (t, *J* = 7.4 Hz, 2H, CH_2_N), 2.51 (s, 3H, SCH_3_), 1.65 (qui, *J* = 7.0 Hz, 2H), 1.43–1.25 (m, 10H), 0.88 (t, *J* = 6.7 Hz, 3H, CH_3_) ppm. ^13^C NMR: (100 MHz, d_6_-acetone): 170.5, 152.4, 151.5, 136.3, 129.5, 128.6, 128.0, 48.8, 42.9, 32.5, 30.0, 29.9, 28.1, 27.5, 23.3, 15.1, 14.3 ppm. LRMS *m*/*z* (ESI): 384.2 (M + Na)^+^. HRMS *m*/*z* (ESI): calculated for C_19_H_27_N_3_NaO_2_S^+^ 384.1709, observed 384.1716. Data and yields for compounds 5b–j are given in ESI.†

Method for preparation of triazinedione amines 6a–j was adapted from the method of Congiu *et al.*^18^ Compound 5a (0.418 g, 1.16 × 10^−3^ mol, 1 eq.) was dissolved under nitrogen with anhydrous toluene (20 mL), ethylenediamine (0.347 g, 0.39 mL, 5.78 × 10^−3^ mol, 5 eq.) was added, and the reaction was heated at reflux (110 °C) for 18 h. The reaction mixture was then cooled to room temperature and the toluene was removed under reduced pressure. The resulting brown residue was resuspended in EtOAc (40 mL), and the organic layer was washed with distilled water (2 × 20 mL) and saturated sodium chloride solution (20 mL). The organic layer was collected and dried (MgSO_4_). The solvent was removed under reduced pressure to afford compound 6a as a yellow liquid (0.232 g, 54%). Samples of compounds 6c, d, g, h, j were further purified by reverse phase C_18_ HPLC using a Kromasil 100-5-C18 column with an elution gradient of H_2_O/MeOH (50 : 50 to 10 : 90) over 50 minutes with a flow rate of 2 mL min^−1^. Fractions containing the desired product were identified using mass spectrometry then combined. Methanol was removed under reduced pressure and the sample was lyophilised to give pure compounds.

Data for compound 6a (0.232 g, 54% yield). ^1^H NMR: (400 MHz, d_6_-acetone): *δ*_H_ 7.48–7.14 (m, 5H), 5.19 (s, 2H, CH_2_Ar), 3.84 (t, *J* = 7.5 Hz, 2H, CH_2_N), 3.52 (t, *J* = 6.2 Hz, 2H, CH_2_N), 3.25 (t, *J* = 6.2 Hz, 2H, CH_2_N), 1.61 (qui, *J* = 8.7 Hz, 2H), 1.39–1.18 (m, 10H), 0.88 (t, *J* = 6.0 Hz, 3H, CH_3_) ppm. ^13^C NMR: (100 MHz, d_6_-acetone): *δ*_C_ 169.0, 154.5, 152.6, 136.3, 129.7, 128.6, 127.6, 50.1, 45.7, 43.2, 42.6, 32.5, 30.1, 30.0, 28.6, 27.6, 23.3, 14.4 ppm. LRMS *m*/*z* (ESI): 374.2 (M + H)^+^. HRMS *m*/*z* (ESI): calculated for C_20_H_32_N_5_O_2_^+^ 374.2544, observed 374.2551. Data and yields for compounds 6b–j are given in ESI.†

Method for preparation of triazinedione guanidines 7a–j was adapted from the method of Congiu *et al.*^18^ Compound 6a (0.100 g, 2.68 × 10^−4^ mol, 1 eq.) was dissolved in hot acetonitrile (50 °C, 20 mL), and was added to 1*H*-pyrazole-1-carboxamidine (0.039 g, 2.68 × 10^−4^ mol, 1 eq.). Di-isopropyl-ethylamine (DIPEA, 0.069 g, 0.093 mL, 5.35 × 10^−4^ mol, 2 eq.) was added, and the resulting solution was then stirred at room temperature for 48 h. A white solid had precipitated, which was collected *via* vacuum filtration and washed with ice-cold acetonitrile to afford compound 7a as a white solid (0.0632 g, 57%).

Data for 7a (57% yield). ^1^H NMR: (400 MHz, d_6_-DMSO) *δ*_H_ 7.43–7.16 (m, 5H), 5.15 (s, 2H, CH_2_Ar), 3.70 (t, *J* = 7.6 Hz, 2H, CH_2_N), 3.36 (t, *J* = 5.7 Hz, 2H, CH_2_N), 3.31 (t, *J* = 5.6 Hz, 2H, CH_2_N), 1.50 (qui, *J* = 7.2 Hz, 2H), 1.32–1.13 (m, 10H), 0.84 (t, *J* = 6.9 Hz, 3H, CH_3_) ppm. ^13^C NMR: (100 MHz, d_6_-DMSO) *δ*_C_ 157.3, 154.2, 153.7, 151.2, 135.7, 128.7, 127.6, 126.8, 44.9, 41.5, 40.3, 39.4, 31.4, 28.9, 28.8, 27.5, 26.5, 22.3, 14.1 ppm. LRMS *m*/*z* (ESI): 416.3 (M + H)^+^. HRMS *m*/*z* (ESI): calculated for C_21_H_34_N7O_2_^+^ 416.2768, observed 416.2765. Data and yields for compounds 7b–j are given in ESI.†

Methods and spectroscopic data for analogues 8–14 are given in ESI.†

#### Procedure for antibacterial MIC determination

*P. fluorescens* Pf-5, *E. coli* (TOP10 or C43), *B. subtilis* (W23) or *M. flavus* colonies were isolated from an agar plate and inoculated in 5 mL of Luria-Bertani broth overnight at 37 °C. *E. coli* C43/pET28a-mraY was grown in Luria-Bertani media containing 50 μg mL^−1^ kanamycin. On a sterile 96 welled plate (which had a sterilised lid), 190 μL of seeded broth (CFU mL^−1^ = 1000) was added to each well. Serial dilutions of 125, 62.5, 31.25, 15.63, 7.82, 3.90, 1.95 and 0.97 μg mL^−1^ test compound were prepared. 10 μL of water, MeOH and DMSO were added to separate wells to serve as a growth control. Each test condition was tested in triplicate. The 96-well plate was covered and incubated overnight at 30 or 37 °C. Optical density (OD_595_) was measured using a HIDEX Sense microplate reader 425–301. The inhibitor concentration which reduced the growth by 50% was measured as the MIC_50_ of the compound.

MIC determination against clinical antibiotic-resistant strains was performed according to Clinical Laboratories Standards Institute M07- A9 by Dr. Jenny Littler (Antimicrobial discovery facility, University of Warwick). Media used was cation-adjusted Mueller Hinton broth (Sigma-Aldrich), and experiments were carried out with a biological repeat. Organisms used: *Enterobacter cloacae* NCTC 13405 (ESBL positive and AmpC enzyme); *Pseudomonas aeruginosa* NCTC 13437 (MDR β-lactams and aminoglycosides); *Acinetobacter baumannii* ATCC 19606 (MDR β-lactams and aminoglycosides); *Klebsiella pneumoniae* ATCC 700603 (ESBL positive and SHV-18); *Enterococcus faecium* ATCC 19434 (vancomycin resistance); *Staphylococcus aureus* MRSA JE2 USA300.

#### alamarBlue cell viability assay

A single colony of *E. coli* was isolated from an agar plate and inoculated in 5 mL of MH2 media overnight at 37 °C with shaking. This starter culture was diluted 100-fold into MH2 media (10 mL), which was then incubated at 37 °C until OD_595_ = 0.8 was reached. On a sterile 96 welled plate (with sterile lid), 90 μL of the seeded broth was added to each well. Test compounds were incubated at a final concentration of 250 μg mL^−1^, in triplicate assays. The 96 well plate and incubated for 1 h at 37 °C. To each well, 10 μL of alamarBlueTM reagent (Invitrogen) was added, and the plate was incubated for a further 2–3 h. Fluorescence was measured on a HIDEX Sense microplate reader 425–301 using an excitation wavelength of 530 nm and emission wavelength of 580 nm.

#### MraY enzyme assays

UDP-MurNAc-l-Ala-γ-d-Glu-l-Lys-d-Ala-d-Ala was prepared enzymatically using the methods described by Lloyd *et al.*,^32^ and was converted to UDP-MurNAc-l-Ala-γ-d-Glu-l-Lys(Nε-dansyl)-d-Ala-d-Ala using the method of Brandish *et al.*^5^ The MraY-catalysed reaction was monitored on a HIDEX Sense microplate reader 425–301 (*λ*_ex_ 340 nm, *λ*_em_ 530 nm). To monitor the formation of dansyl-lipid I, membranes containing overexpressed *E. coli* MraY (15 μL of 0.6 mg mL^−1^ stock) were incubated with UDP-MurNAc-l-Ala-γ-d-Glu-l-Lys(Nε-dansyl)-d-Ala-d-Ala (25 μM), undecaprenyl phosphate (30 μM, from stock solution in 50 μM Tris buffer pH 7.5 containing 2 mM β-mercaptoethanol, 1 mM MgCl_2_, 20% glycerol, 0.5% Triton X-100), in 100 mM Tris buffer pH 7.5 containing 25 mM MgCl_2_, in a total volume of 100 μL. Test compounds were assayed at 200 μM final concentration, in triplicate assays, and compound 6d was assayed at 25–200 μM concentration in triplicate assays. Tunicamycin (20 μM) was used as a positive control for enzyme inhibition assays. Membranes containing overexpressed *E. coli* R288L mutant MraY were prepared as described in Rodolis *et al.*^16^ Radiochemical assays were carried out using membranes containing overexpressed *E. coli* MraY as described in Mihalyi *et al.*^19^

### Computational methods

To generate a structure of *Escherichia coli* MraY, AlphaFold2 was used,^23^ inputting the amino acid sequence of *E. coli* MraY (Uniprot). Docking simulations were performed either with Schrodinger Maestro software, using ProteinPrep, LigPrep, and GridGen functions, or with SwissDock with Attracting Cavities.^24^ Docking returned output files as pdb files, that were analysed using Pymol.

## Data availability

The primary data supporting this work are included in the ESI.†

## Conflicts of interest

There is no conflict of interest to declare.

## Supplementary Material

MD-OLF-D4MD00937A-s001
