# Supplementary material for: Antimicrobial triazinedione inhibitors of the translocase MraY–protein E interaction site: synergistic effects with bacitracin imply a new mechanism of action
Source: RSC Med Chem. 2025 Jan 30;16(4):1641–53. doi: 10.1039/d4md00937a (PMC11812184; doi:10.1039/d4md00937a)

Supplementary Information (SI) for RSC Medicinal Chemistry.
This journal is © The Royal Society of Chemistry 2025

Antimicrobial triazinedione inhibitors of the translocase MraY-protein E interaction site: synergistic effects with bacitracin imply a new mechanism of action

Julia A. Fairbairn, Rachel V. Kerr, Nika-Kare A. Pierre-White, Anthony Jacovides, Becca W.A. Baileeves, Phillip J. Stansfeld, Gerhard Bringmann, Andrew T. Merritt, Timothy D.H. Bugg*

Supporting Information

Table S1. MIC_{90} values for triazinedione compounds against *E. coli* C43 overexpressing *E. coli* MraY
Table S2. Binding scores (Glidescore) for the top 50 poses predicted by the docking model of the peptidomimetics with *E. coli* MraY.

Figure S1. Predicted binding interactions for docked structure (using Schrodinger Maestro) of compound **6h** in binding site 1 in *E. coli* MraY.
Figure S2. Predicted binding interactions for docked structure (using Schrodinger Maestro) of compound **6j** in binding site 2 in *E. coli* MraY.
Figure S3. Three lowest energy poses (using SwissDock) for compound **7j** near helix 9 in *E. coli* MraY
Figure S4. Three lowest energy poses (using SwissDock) for Arg-Trp octyl ester near helix 9 in *E. coli* MraY
Figure S5. Alignment of MraY sequences near Phe-165, Phe-175 in helix 5
Figure S6. Sequence alignment of protein E sequences from bacteriophage fX174 and 3 other Microviridae, showing conserved Phe-12.
Figure S7. Docked conformation of michellamine B with hydrophobic cleft near helix 9 of MraY.

Supporting Experimental Data:
Spectroscopic data for compounds **5a-j**, **6a-j**, **7a-j**
^{1}H NMR and ^{13}C NMR spectra for compounds **6a-j**, **7a-j**
Methods and spectroscopic data for analogues **8-14**
^{1}H and ^{13}C NMR spectra for final compounds

			MIC$_{90}$ (μg/mL)		
Compound	R_1	R_2	*E. coli* C43	C43/ pET28a	C43/ pET28a-mraY
**6a**	H	amine	16	16	16
**6b**	4-OCH_3	amine	16	16	16
**6d**	2-OCH_3	amine	64	128	128
**6j**	4-CF_3	amine	64	8	8
**7a**	H	guanidine	16	16	16
**7b**	4-OCH_3	guanidine	16	16	16
**7j**	4-CF_3	guanidine	4	4	4
**13**	bis-aryl	amine	256	256	128

Table S1. MIC$_{90}$ values for triazinedione compounds against *E. coli* C43 containing pET28a-mraY overexpressing *E. coli* MraY, compared with *E. coli* C43 containing empty pET28a vector, and *E. coli* C43 containing no plasmid.

Compound	Aromatic substituent	Amine/ guanidine	GlideScores
**Binding site 1**			
**6h**	4-NO_2	Amine	-6.641
**6d**	2-OCH_3	Amine	-6.546, -5.684, -5.627
**6e**	4-F	Amine	-6.090, -5.883, -5.783
**6a**	H	Amine	-6.033, -5.936, -4.935
**7a**	H	Guanidine	-5.918
**6b**	4-OCH_3	Amine	-5.886, -5.822, -5.780
**7b**	4-OCH_3	Guanidine	-5.626, -5.534
**7d**	2-OCH_3	Guanidine	-5.366, -5.054
**7f**	4-Cl	Guanidine	-5.289, -4.813
**7j**	4-CF_3	Guanidine	-5.179, -5.151, -5.063
**7g**	4-Br	Guanidine	-5.078, -5.054
**7c**	3-OCH_3	Guanidine	-4.732, -4.472
**Binding site 2**			
**6j**	4-CF_3	Amine	-6.192, -4.776, -4.528
**6c**	3-OCH_3	Amine	-5.644, -5.097, -4.520
**6b**	4-OCH_3	Amine	-5.608
**6f**	4-Cl	Amine	-5.110, -5.077, -4.649
**7f**	4-Cl	Guanidine	-5.023
**7c**	3-OCH_3	Guanidine	-4.956
**7a**	H	Guanidine	-4.804, -4.615
**6g**	4-Br	Amine	-4.751, -4.588, -3.988
**7g**	4-Br	Guanidine	-4.560
**7h**	4-NO_2	Guanidine	-4.555, -3.634, -3.448
**7e**	4-F	Guanidine	-4.512, -4.507, -4.375

Table S2. Binding scores (Glidescore) for the top 50 poses predicted by the docking model of the peptidomimetics with *E. coli* MraY.

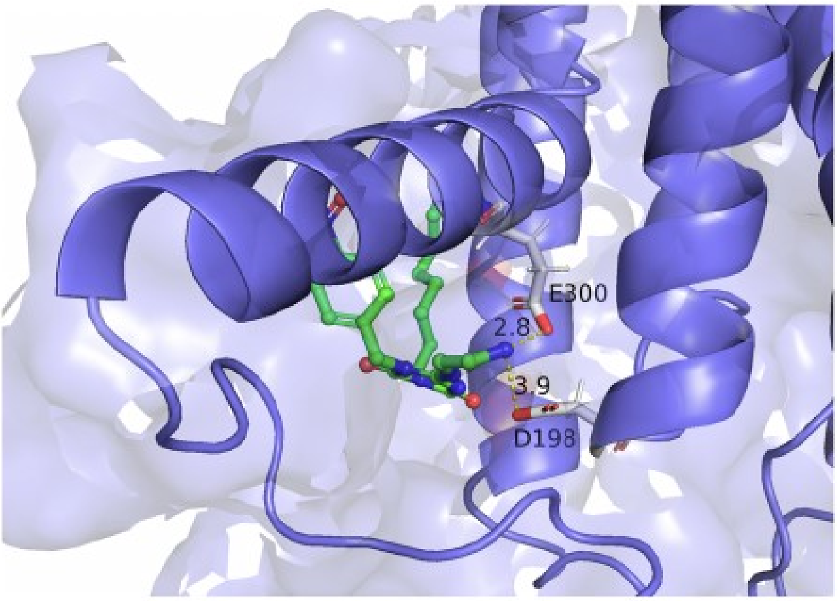

Figure S1. Predicted binding interactions for docked structure (using Schrodinger Maestro) of compound **6h** in binding site 1 in *E. coli* MraY, showing interactions between the amine sidechain of **6h** with the sidechains of Glu-300 and Asp-198 of MraY.

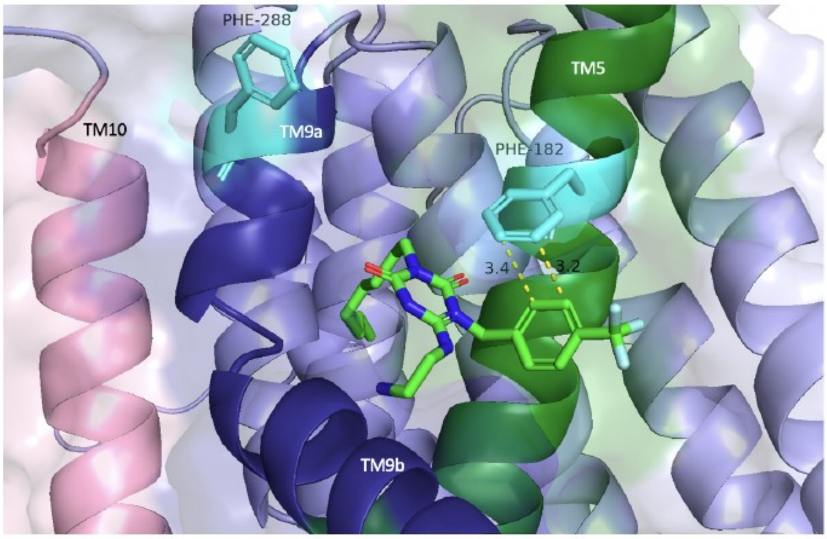

Figure S2. Predicted binding interactions for docked structure (using Schrodinger Maestro) of compound **6j** in binding site 2 in *E. coli* MraY, showing π–π interactions between the aromatic sidechain of **6j** and the sidechain of Phe-182 in helix 5 of MraY.

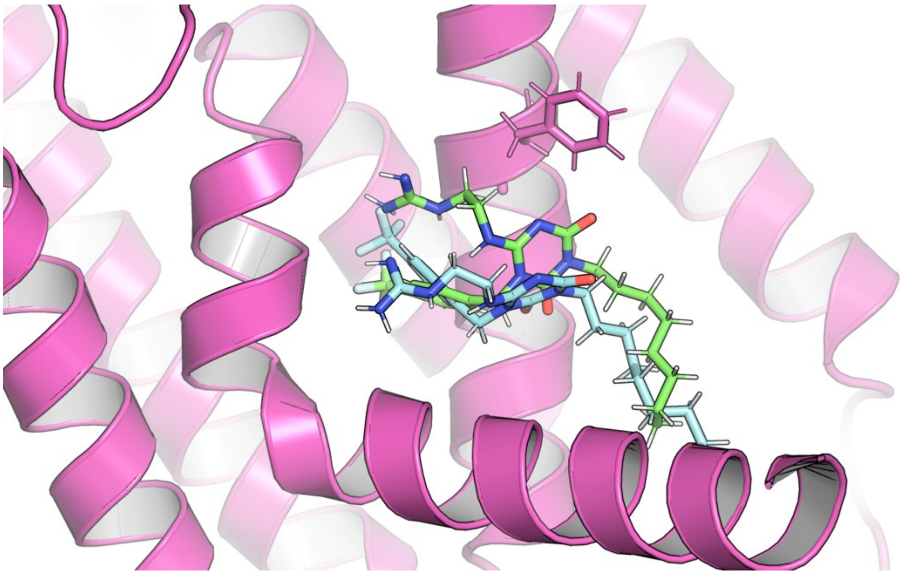

Figure S3. Three lowest energy poses (using SwissDock) for compound **7j** near helix 9 in *E. coli* MraY, showing the sidechain of Phe-182 in helix 5 of MraY.

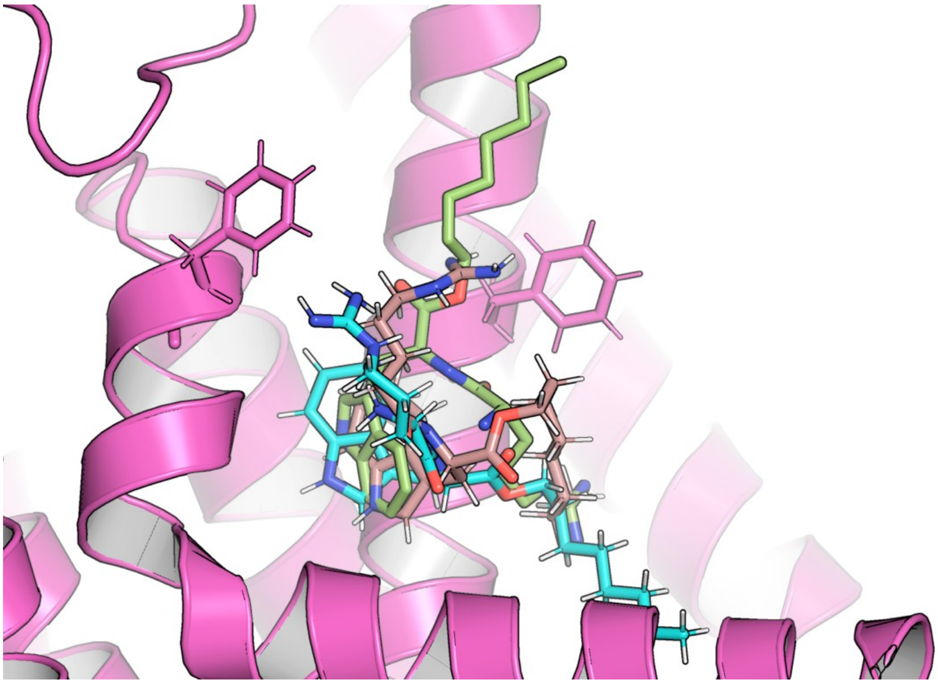

Figure S4. Three lowest energy poses (using SwissDock) for Arg-Trp octyl ester near helix 9 in *E. coli* MraY, showing the sidechains of Phe-182 on helix 5, and Phe-288 on helix 9.

	Helix 5<br>175 182
**Enterobacteriaceae**	
Escherichia coli	LGLFYILLAYFVIVGT
Enterobacter sp.	LGLFYILLAYFVIVGT
S. typhimurium	LGLFYILLSYFVIVGT
K. pneumoniae	LGLLYILLAYFVIVGT
**Other Gram-negative:**	
Vibrio cholerae	LGLMYIVLTYFVIVGT
H. influenzae	LGLFYIVLSYFVIVGT
Acinetobacter baumannii	LGLAFIVFTVLVINGA
Pseudomonas aeruginosa	LGIFFVVLTYFVIVGS
P. fluorescens Pf-5	LGAGFIVLTYFVIVGS
Burkholderia cepacia	GVWGFIVLTYLVIVGA
**Gram positive:**	
Micrococcus luteus	AGPAIGVILFVIWSNL
M. tuberculosis	LFVLFCVVIVSAWSNA
Rhodococcus jostii	VFVAVCYLLVSAWSNA
Enterococcus faecalis	FYGVFIIFWLGVFSNA
Enterococcus faecium	IYGIFAIFWLGVFSNA
S. pneumoniae	FYIFFALFWLGVFSNA
S. aureus	AYVIFIVFWQVGFSNA
B. subtilis	LGWAYFILVLFMLVGG
A. aeolicus	LYVDLGVLYLPFAVFV

Figure S5. Alignment of MraY sequences near Phe-175 & Phe-182 in helix 5

φX174	M	V	R	W	T	L	W	D	T	L	A	F	L	L	L	L	S	L	L	L	P	S	L	L	I	M	F	I	P	S	T	F	K	R	P	V	S
A3	M	E	R	W	T	L	L	D	I	L	A	F	L	L	L	L	S	L	L	L	P	S	L	L	I	M	F	I	P	S	M	Y	K	Q	H	A	S
G4	M	E	H	W	T	L	S	G	I	L	A	F	L	L	L	L	S	L	F	L	P	S	L	L	I	T	F	I	P	L	T	S	K	P	P	V	S
φK	M	E	R	W	T	L	S	A	I	L	A	F	L	L	L	L	S	L	L	L	P	S	L	L	I	M	F	I	P	S	T	F	R	Q	H	A	S

Figure S6. Sequence alignment of protein E sequences from bacteriophage φX174 and 3 other Microviridae, showing conserved Phe-12 (highlighted in green).

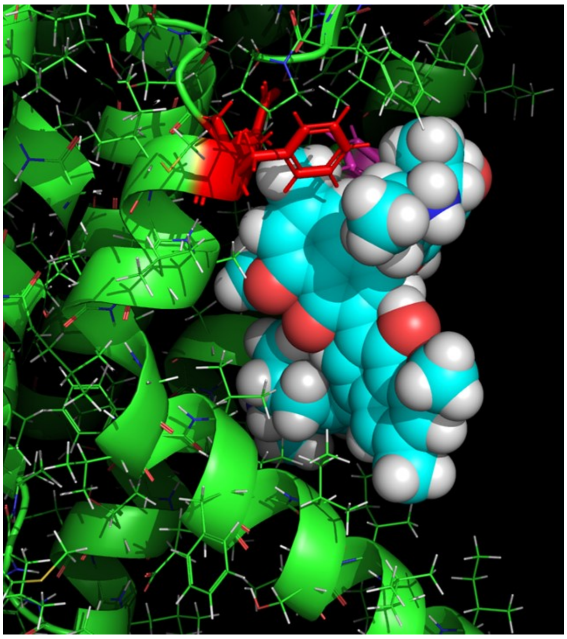

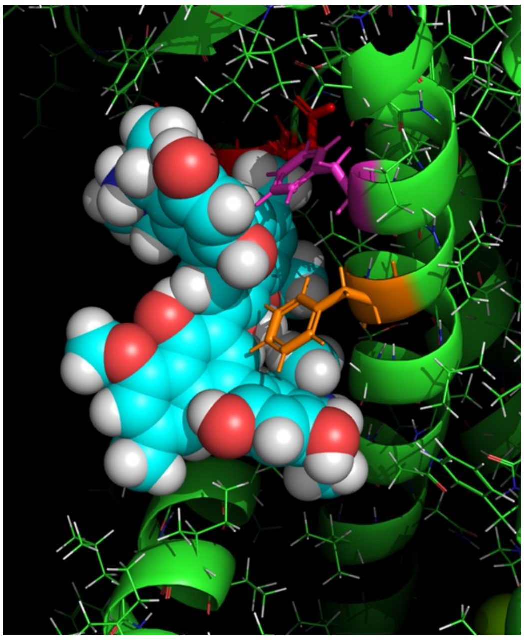

Figure S7. Docked conformation of michellamine B (from reference 19) with hydrophobic cleft near helix 9 of MraY. Left panel, view shows interaction with Phe-288 (in red) of helix 9a, and helix 9b. Right panel, view shows interaction with Phe-182 (in orange) and Phe-175 (in magenta) of helix 5.

Spectroscopic data for compounds 5a-j

Compound **5a** was isolated via method A (0.418 g, 48%). R_f 0.73 (1:1 EtOAc: petroleum ether). ^{1}H NMR: (400 MHz, d_6-acetone) δ_H: 7.40 – 7.29 (m, 5H), 5.19 (s, 2H, CH_2Ar), 3.86 (t, J = 7.4 Hz, 2H, CH_2N), 2.51 (s, 3H, SCH_3), 1.65 (qui, J = 7.0 Hz, 2H), 1.43 – 1.25 (m, 10H), 0.88 (t, J = 6.7 Hz, 3H, CH_3) ppm. ^{13}C NMR: (100 MHz, d_6-acetone): 170.5, 152.4, 151.5, 136.3, 129.5, 128.6, 128.0, 48.8, 42.9, 32.5, 30.0, 29.9, 28.1, 27.5, 23.3, 15.1, 14.3 ppm. LRMS *m/z* (ESI): 384.2 $(M+Na)^+$. HRMS *m/z* (ESI): calculated for $C_{19}H_{27}N_3NaO_2S^+$ 384.1709, observed 384.1716.

Compound **5b** was prepared using 4-methoxybenzyl chloride, and was isolated via method A as a white solid (0.419 g, 41%). R_f = 0.76 (1:1 EtOAc: petroleum ether). ^{1}H NMR: (300 MHz, d_6-acetone) δ_H 7.33 (d, J = 8.3 Hz, 2H, aryl 2'-H), 6.90 (d, J = 8.4 Hz, 2H, aryl 3'-H), 5.11 (s, 2H, CH_2Ar), 3.86 (t, J = 7.0 Hz, 2H, CH_2N), 3.78 (s, 3H, OCH_3), 2.52 (s, 3H, SCH_3), 1.64 (qui, J = 7.3 Hz, 2H), 1.39 – 1.21 (m, 10H), 0.88 (t, J = 5.7 Hz, 3H, CH_3) ppm. ^{13}C NMR: (75 MHz, d_6-acetone) δ_C 170.4, 160.4, 152.4, 151.2, 129.8, 128.1, 114.8, 55.5, 48.3, 42.8, 32.5, 30.0, 30.0, 28.1, 27.5, 23.3, 15.1, 14.3 ppm. LRMS *m/z* (ESI): 392.2 $(M+H)^+$, 414.1 $(M+Na)^+$. HRMS *m/z* (ESI): calculated for $C_{20}H_{29}N_3NaO_3S^+$ 414.1822, observed 414.1817.

Compound **5c** was prepared using 3-methoxybenzyl bromide, and was isolated by method B as a yellow solid (0.719 g, 62 %). R_f 0.20 (9:1 Petroleum ether: EtOAc). ^{1}H NMR: (400 MHz, d_6-acetone) δ_H 7.26 (t, J = 8.1 Hz, 1H, aryl 5'-H), 6.99 – 6.82 (m, 3H), 5.15 (s, 2H, CH_2Ar), 3.86 (t, J = 7.1 Hz, 2H, CH_2N), 3.78 (s, 3H, OCH_3), 2.50 (s, 3H, SCH_3), 1.65 (qui, J = 7.1 Hz, 2H), 1.39 – 1.22 (m, 10H), 0.88 (t, J = 6.4 Hz, 3H, CH_3) ppm. ^{13}C NMR: (100 MHz, d_6-acetone) δ_C 170.5, 161.0, 152.5, 151.6, 137.8, 130.6, 120.1, 114.0, 113.8, 55.5, 48.7, 42.8, 32.6, 30.1, 30.0, 28.2, 27.6, 23.4 ,15.2, 14.4 ppm. LRMS *m/z* (ESI): 414.2 $(M+Na)^+$. HRMS *m/z* (ESI): calculated for $C_{20}H_{29}N_3NaO_3S^+$ 414.1822, observed 414.1818.

Compound **5d** was prepared using 2-methoxybenzyl chloride, and was isolated by method B as a yellow liquid (0.766 g, 67%). R_f 0.63 (9:1 Petroleum ether: EtOAc). ^{1}H NMR: (400 MHz, d_6-acetone) δ_H 7.42 (d, J = 7.8 Hz, 1H, aryl 6'-H), 7.31 (t, J = 8.2 Hz, 1H, aryl 4'-H), 7.21 (t, J = 7.8 Hz, 1H, aryl 5'-H), 7.03 (d, J = 8.3 Hz, 1H, aryl 3'-H), 5.17 (s, 2H, CH_2Ar), 3.87 (t, J = 7.3 Hz, 2H, CH_2N), 3.82 (s, 3H, OCH_3), 2.48 (s, 3H, SCH_3), 1.65 (qui, J = 7.4 Hz, 2H), 1.37 – 1.24 (m, 10H), 0.89 (t, J = 7.9 Hz, 3H, CH_3) ppm. ^{13}C NMR: (100 MHz, d_6-acetone) δ_C 170.7, 157.7, 152.6, 151.3, 131.3, 129.6, 128.6, 128.0, 126.7, 56.0, 44.3, 42.8, 32.5, 30.0, 30.0, 28.1, 27.5, 23.3, 15.0, 14.3 ppm. LRMS *m/z* (ESI): 392.1 $(M+H)^+$, 414.1 $(M+Na)^+$. HRMS *m/z* (ESI): calculated for $C_{20}H_{29}N_3NaO_3S^+$ 414.1822, observed 414.1817.

Compound **5e** was prepared using 4-fluorobenzyl bromide, and was isolated by method A as a yellow solid (0.742 g, 66%). $R_f = 0.68$ (1:1 EtOAc: petroleum ether). ^{1}H NMR: (400 MHz, d_6-acetone) δ_H 7.50 – 7.40 (m, 2H), 7.16 – 7.02 (m, 2H), 5.17 (s, 2H, CH_2Ar), 3.85 (t, J = 7.0 Hz, 2H, CH_2N), 2.52 (s, 3H, SCH3), 1.63 (qui, J = 7.3 Hz, 2H), 1.41 – 1.22 (m, 10H), 0.88 (t, J = 6.4 Hz, 3H, CH_3) ppm. ^{13}C NMR: (100 MHz, d_6-acetone) δ_C 170.3, 160.4, 152.4, 151.5, 132.4, 130.4, 116.0, 48.2, 42.9, 32.5, 30.0, 29.9, 28.1, 27.5, 23.3, 15.1, 14.3 ppm. LRMS *m/z* (ESI): 380.1 $(M+H)^+$, 402.1 $(M+Na)^+$. HRMS *m/z* (ESI): calculated for $C_{19}H_{26}FN_3NaO_2S^+$ 402.1622, observed 402.1618.

Compound **5f** was prepared using 4-chlorobenzyl bromide, and was isolated by method A as a white solid (0.576 g, 55%). R_f 0.67 (1:1 Petroleum ether: EtOAc). ^{1}H NMR: (400 MHz, d_6-acetone) δ_H 7.46 – 7.36 (m, 4H), 5.18 (s, 2H, CH_2Ar), 3.85 (t, J = 7.3 Hz, 2H, CH_2N), 2.52 (s, 3H, SCH3), 1.63 (qui, J = 7.3 Hz, 2H), 1.38 – 1.22 (m, 10H), 0.88 (t, J = 6.8 Hz, 3H, CH_3) ppm. ^{13}C NMR: (100 MHz, d_6-acetone) δ_C 170.2, 152.4, 151.5, 135.2, 134.0, 130.0, 129.5, 48.2, 42.9, 32.5, 30.0, 29.9, 28.1, 27.5, 23.3, 15.1, 14.3 ppm. LRMS *m/z* (ESI): 396.1 $(M+H)^+$, 418.0 $(M+Na)^+$. HRMS *m/z* (ESI): calculated for $C_{19}H_{26}ClN_3NaO_2S^+$ 418.1326, observed 418.1322.

Compound **5g** was prepared using 4-bromobenzyl bromide, and was isolated by method A as a cream solid (1.23 g, 95%). $R_f = 0.32$ (9:1 Petroleum ether: EtOAc). ^{1}H NMR: (400 MHz, d_6-DMSO) δ_H 7.58 (d, J = 8.4 Hz, 2H), 7.35 (d, J = 8.6 Hz, 2H), 5.06 (s, 2H, CH_2Ar), 3.74 (t, J = 7.5 Hz, 2H, CH_2N), 2.47 (s, 3H, SCH_3), 1.54 (qui, J = 7.3 Hz, 2H), 1.32 – 1.22 (m, 10H), 0.85 (t, J = 6.7 Hz, 3H, CH_3) ppm. ^{13}C NMR: (100 MHz, d_6-DMSO) δ_C 169.5, 151.6, 150.2, 134.5, 131.5, 128.5, 120.8, 47.3, 41.8, 31.2, 28.6, 28.7, 26.8, 26.2, 22.1, 14.7, 13.9 ppm. LRMS *m/z* (ESI): 462.1 $(M+Na)^+$. HRMS *m/z* (ESI): calculated for $C_{19}H_{26}BrN_3NaO_2S^+$ 462.0821, observed 462.0822.

Compound **5h** was prepared using 4-nitrobenzyl bromide, and was isolated by method B as a yellow liquid (0.868 g, 83%). R_f 0.44 (1:1 EtOAc: petroleum ether). ^{1}H NMR: (400 MHz, d_6-acetone) δ_H 8.23 (d, J = 78.9 Hz, 2H), 7.69 (d, J = 8.9 Hz, 2H), 5.35 (s, 2H, CH_2Ar), 3.86 (t, J = 7.5 Hz, 2H, CH_2N), 2.53 (s, 3H, SCH_3), 1.68 – 1.60 (m, 2H), 1.31 – 1.24 (m, 10H), 0.87 (t, J = 6.4 Hz, 3H, CH_3) ppm. ^{13}C NMR: (100 MHz, d_6-acetone) δ_C 170.2, 152.4, 151.5, 148.5, 143.9, 129.1, 124.5, 48.4, 43.0, 32.5, 30.0, 29.9, 28.1, 27.5, 23.3, 15.1, 14.3 ppm. LRMS *m/z* (ESI): 407.2 $(M+H)^+$, 429.2 $(M+Na)^+$. HRMS *m/z* (ESI): calculated for $C_{19}H_{26}N_4NaO_4S^+$ 429.1567, observed 429.1562.

Compound **5j** was prepared using 4-trifluoromethylbenzyl bromide, and was isolated by method B as a pale-yellow solid (0.238 g, 19%). R_f 0.28 (9:1 petroleum ether:EtOAc). ^{1}H

NMR: (400 MHz, d_6-acetone) δ_H 7.71 (d, J = 8.4 Hz, 2H), 7.62 (d, J = 8.1 Hz, 2H), 5.30 (s, 2H, CH_2Ar), 3.86 (t, J = 7.4 Hz, 2H, CH_2N), 2.52 (s, 3H, SCH_3), 1.64 (qui, J = 7.4 Hz, 2H), 1.32 – 1.26 (m, 10H), 0.88 (t, J = 7.7 Hz, 3H, CH_3) ppm. ^{13}C NMR: (100 MHz, d_6-acetone) δ_C 170.3, 152.4, 151.5, 142.0, 141.0, 130.3, 128.7, 117.7, 48.4, 42.9, 32.5, 30.0, 29.9, 28.1, 27.5, 23.3, 15.1, 14.3 ppm. LRMS *m/z* (ESI): 430.2 $(M+H)^+$, 452.1 $(M+Na)^+$. HRMS *m/z* (ESI): calculated for $C_{20}H_{26}F_3N_3NaO_2S^+$: 452.1590, observed 452.1584.

Spectroscopic data for compounds 6a-j

Data for compound **6a** (0.232 g, 54% yield). ^1H NMR: (400 MHz, d_6-acetone): δ_H 7.48 – 7.14 (m, 5H), 5.19 (s, 2H, CH_2Ar), 3.84 (t, J = 7.5 Hz, 2H, CH_2N), 3.52 (t, J = 6.2 Hz, 2H, CH_2N), 3.25 (t, J = 6.2 Hz, 2H, CH_2N), 1.61 (qui, J = 8.7 Hz, 2H), 1.39 – 1.18 (m, 10H), 0.88 (t, J = 6.0 Hz, 3H, CH_3) ppm. ^{13}C NMR: (100 MHz, d_6-acetone): δ_C 169.0, 154.5, 152.6, 136.3, 129.7, 128.6, 127.6, 50.1, 45.7, 43.2, 42.6, 32.5, 30.1, 30.0, 28.6, 27.6, 23.3, 14.4 ppm. LRMS *m/z* (ESI): 374.2 $(M+H)^+$. HRMS *m/z* (ESI): calculated for $C_{20}H_{32}N_5O_2^+$ 374.2544, observed 374.2551.

Data for compound **6b** (0.287 g, 70 % yield). ^1H NMR: (400 MHz, d_6-acetone) δ_H 7.28 (d, J = 8.7 Hz, 2H, aryl 2'-H), 6.89 (d, J = 8.8 Hz, 2H, aryl 3'-H), 5.10 (s, 2H, CH_2Ar), 3.82 (t, J = 7.2 Hz, 2H, CH_2N), 3.77 (s, 3H, OCH_3), 3.51 (t, J = 6.1 Hz, 2H, CH_2N), 3.25 (t, J = 6.1 Hz, 2H,CH_2N), 1.65 – 1.54 (m, 2H), 1.36 – 1.19 (m, 10H), 0.87 (t, J = 7.0 Hz, 3H, CH_3) ppm. ^{13}C NMR: (100 MHz, d_6-acetone) δ_C 169.3, 160.3, 154.6, 152.6, 129.2, 128.1, 115.0, 55.6, 50.1, 45.2, 43.1, 42.6, 32.5, 30.2, 30.0, 28.6, 27.6, 23.3, 14.4 ppm. LRMS *m/z* (ESI): 404.2 $(M+H)^+$, 426.2 $(M+Na)^+$. HRMS *m/z* (ESI): calculated for $C_{21}H_{33}N_5NaO_3^+$ 426.2476, observed 426.2470.

Data for compound **6c** (0.546 g, 72%). HPLC retention time 34.3 min. ^1H NMR: (400 MHz, CD_3OD) δ_H 7.28 (t, J = 8.2 Hz, 1H, aryl 5'-H), 6.95 – 6.75 (m, 3H), 5.14 (s, 2H, CH_2Ar), 3.87 (t, J = 7.3 Hz, 2H, CH_2N), 3.78 (s, 3H, OCH_3), 3.44 (t, J = 6.2 Hz, 2H, CH_2N), 2.75 (t, J = 6.2 Hz, 2H, CH_2N), 1.69 – 1.58 (m, 2H), 1.39 – 1.20 (m, 10H), 0.90 (t, J = 6.4 Hz, 3H, CH_3) ppm. ^{13}C NMR: (100 MHz, CD_3OD) δ_C 175.6, 157.3, 156.2, 152.8, 138.4, 131.1, 119.2, 114.1, 113.4, 55.7, 46.0, 44.6, 43.4, 41.4, 32.9, 30.4, 30.3, 28.8, 27.8, 23.7, 14.4 ppm. LRMS *m/z* (ESI): 404.2 $(M+H)^+$. HRMS *m/z* (ESI): calculated for $C_{21}H_{34}N_5O_3^+$ 404.2656, observed 404.2653.

Data for compound **6d** (0.339 g, 43%). HPLC retention time: 34.7 min. ^1H NMR: (400 MHz, CD_3OD) δ_H 7.24 (t, J = 8.7 Hz, 1H, aryl 4'-H), 7.04 (d, J = 7.6 Hz, 1H, aryl 6'-H), 6.96 (d, J = 8.3 Hz, 1H, aryl 3'-H), 6.88 (t, J = 7.5 Hz, 1H, aryl 5'-H), 5.07 (s, 2H, CH_2Ar), 3.89 (t, J = 7.4 Hz, 2H, CH_2N), 3.81 (s, 3H, OCH_3), 3.46 (t, J = 6.2 Hz, 2H, CH_2N), 2.80 (t, J = 6.3 Hz, 2H,

CH_2N), 1.68 – 1.54 (m, 2H), 1.40 – 1.17 (m, 10H), 0.89 (t, J = 7.0 Hz, 3H, CH_3) ppm. ^{13}C NMR: (100 MHz, CD_3OD) δ_C 170.2, 155.8, 155.3, 154.5, 134.9, 129.6, 128.1, 121.4, 111.6, 56.0, 44.0, 43.3, 42.8, 41.6, 32.9, 30.3, 30.2, 28.8, 27.7, 23.7, 14.4 ppm. LRMS *m/z* (ESI): 404.2 $(M+H)^+$. HRMS *m/z* (ESI): calculated for $C_{21}H_{34}N_5O_3^+$ 404.2656, observed 404.2653.

Data for compound **6e** (0.429 g, 56%). ^1H NMR: (300 MHz, d_6-acetone) δ_H 7.42 – 7.36 (m, 2H), 7.11 – 7.02 (m, 2H), 5.19 (s, 2H, CH_2Ar), 3.83 (t, J = 7.4 Hz, 2H, CH_2N), 3.54 (t, J = 6.0 Hz, 2H, CH_2N), 3.28 (t, J = 6.1 Hz, 2H, CH_2N), 1.67 – 1.55 (m, 2H), 1.37 – 1.24 (m, 10H), 0.88 (t, J = 6.8 Hz, 3H, CH_3) ppm. ^{13}C NMR: (75 MHz, d_6-acetone) δ_C 164.7, 161.0, 154.8, 152.5, 132.4, 129.9, 116.3, 49.9, 45.0, 43.1, 42.6, 32.5, 30.0, 30.0, 28.6, 27.6, 23.3, 14.3 ppm. LRMS *m/z* (ESI): 392.2 $(M+H)^+$. HRMS *m/z* (ESI): calculated for $C_{20}H_{31}FN_5O_2^+$ 392.2456, observed 392.2450.

Data for compound **6f** (0.436 g, 75%). ^1H NMR: (400 MHz, d_6-acetone) δ_H 7.44 – 7.31 (m, 4H), 5.20 (s, 2H, CH_2Ar), 3.82 (t, J = 7.6 Hz, 2H, CH_2N), 3.53 (t, J = 6.1 Hz, 2H, CH_2N), 3.27 (t, J = 6.0 Hz, 2H, CH_2N), 1.66 – 1.53 (m, 2H), 1.36 – 1.24 (m, 10H), 0.88 (t, J = 5.9 Hz, 3H, CH_3) ppm. ^{13}C NMR: (100 MHz, d_6-acetone) δ_C 159.7, 151.6, 150.7, 135.3, 129.9, 129.6, 129.5, 50.1, 45.1, 43.1, 42.6, 32.5, 30.0, 29.9, 28.6, 27.6, 23.3, 14.4 ppm. LRMS *m/z* (ESI): 408.1 $(M+H)^+$. HRMS *m/z* (ESI): calculated for $C_{20}H_{31}ClN_5O_2^+$: 408.2161, observed 408.2157.

Data for compound **6g** (0.668 g, 54 %). HPLC retention time: 35.3 min. ^1H NMR: (400 MHz, CD_3OD) δ_H 7.52 (d, J = 8.6 Hz, 2H), 7.18 (d, J = 8.6 Hz, 2H), 5.11 (s, 2H, CH_2Ar), 3.85 (t, J = 7.4 Hz, 2H, CH_2N), 3.45 (t, J = 6.2 Hz, 2H, CH_2N), 2.78 (t, J = 6.3 Hz, 2H, CH_2N), 1.67 – 1.57 (m, 2H), 1.37 – 1.17 (m, 10H), 0.90 (t, J = 6.6 Hz, 3H, CH_3) ppm. ^{13}C NMR: (100 MHz, CD_3OD) δ_C 158.6, 156.1, 152.7, 135.6, 133.0, 129.3, 122.6, 45.7, 43.4, 41.4, 39.4, 32.9, 30.4, 29.9, 28.8, 27.8, 23.7, 14.4 ppm. LRMS *m/z* (ESI): 452.1 $(M+H)^+$. HRMS *m/z* (ESI): calculated for $C_{20}H_{31}BrN_5O_2^+$ 452.1656, observed 452.1655.

Data for compound **6h** (0.513 g, 58%). HPLC retention time: 33.4 min. ^1H NMR: (400 MHz, d_6-acetone) δ_H 8.22 (d, J = 8.7 Hz, 2H), 7.63 (d, J = 8.9 Hz, 2H), 5.38 (s, 2H, CH_2Ar), 3.82 (t, J = 7.6 Hz, 2H, CH_2N), 3.54 (t, J = 6.1 Hz, 2H, CH_2N), 3.28 (t, J = 5.9 Hz, 2H, CH_2N), 1.61 (qui, J = 7.0 Hz, 2H), 1.36 – 1.22 (m, 10H), 0.87 (t, J = 7.6 Hz, 3H, CH_3) ppm.^{13}C NMR: (100 MHz, d_6-acetone) δ_C 154.6, 152.4, 152.1, 148.3, 144.1, 128.8, 124.6, 50.2, 45.4, 43.2, 42.7, 32.6, 30.4, 30.0, 28.6, 27.6, 23.3, 14.4 ppm. LRMS *m/z* (ESI): 419.1 $(M+H)^+$. HRMS *m/z* (ESI): calculated for $C_{20}H_{31}N6O_4^+$: 419.2401, observed 419.2399.

Data for compound **6j** (0.896 g, 51%). HPLC retention time: 35.3 min. ^1H NMR: (400 MHz, CD_3OD) δ_H 7.68 (d, J = 8.3 Hz, 2H), 7.43 (d, J = 8.2 Hz, 2H), 5.23 (s, 2H, CH_2Ar), 3.86 (t, J = 7.2 Hz, 2H, CH_2N), 3.45 (t, J = 6.3 Hz, 2H, CH_2N), 2.79 (t, J = 6.3 Hz, 2H, CH_2N), 1.69 – 1.54

(m, 2H), 1.37 – 1.14 (m, 10H), 0.89 (t, J = 6.8 Hz, 3H, CH_3) ppm. ^{13}C NMR: (100 MHz, CD_3OD) δ_C 156.5, 154.0, 151.3, 130.1, 128.8, 127.9, 126.8, 118.1, 46.0, 43.4, 42.7, 41.4, 32.9, 30.3, 28.8, 28.0, 27.9, 23.7, 14.4 ppm. LRMS *m/z* (ESI): 442.2 $(M+H)^+$. HRMS *m/z* (ESI): calculated for $C_{21}H_{31}F_3N_5O_2^+$ 442.2424, observed 442.2421.

Spectroscopic data for compounds 7a-j

Data for **7a** (57% yield). ^{1}H NMR: (400 MHz, d_6-DMSO) δ_H 7.43 – 7.16 (m, 5H), 5.15 (s, 2H, CH_2Ar), 3.70 (t, J = 7.6 Hz, 2H, CH_2N), 3.36 (t, J = 5.7 Hz, 2H, CH_2N), 3.31 (t, J = 5.6 Hz, 2H, CH_2N), 1.50 (qui, J = 7.2 Hz, 2H), 1.32 – 1.13 (m, 10H), 0.84 (t, J = 6.9 Hz, 3H, CH_3) ppm. ^{13}C NMR: (100 MHz, d_6-DMSO) δ_C 157.3, 154.2, 153.7, 151.2, 135.7, 128.7, 127.6, 126.8, 44.9, 41.5, 40.3, 39.4, 31.4, 28.9, 28.8, 27.5, 26.5, 22.3, 14.1 ppm. LRMS *m/z* (ESI): 416.3 $(M+H)^+$. HRMS *m/z* (ESI): calculated for $C_{21}H_{34}N7O_2^+$ 416.2768, observed 416.2765.

Data for **7b** (0.076 g, 48%). ^{1}H NMR: (400 MHz, CD_3OD) δ_H 7.21 (d, J = 8.9 Hz, 2H, aryl 2'-H), 6.90 (d, J = 8.8 Hz, 2H, aryl 3'-H), 5.08 (s, 2H, CH_2Ar), 3.86 (t, J = 7.4 Hz, 2H, CH_2N), 3.77 (s, 3H, OCH_3), 3.51 (t, J = 6.8 Hz, 2H, CH_2N), 3.36 (t, J = 6.8 Hz, 2H, CH_2N), 1.63 (qui, J = 7.1 Hz, 2H), 1.39 – 1.20 (m, 10H), 0.90 (t, J = 6.7 Hz, 3H, CH_3) ppm. ^{13}C NMR: (100 MHz, CD_3OD) δ_C 160.9, 159.0, 157.1, 156.4, 152.5, 129.0, 127.8, 115.3, 55.8, 45.9, 43.4, 41.4, 41.3, 32.9, 30.4, 30.3, 28.7, 27.8, 23.7, 14.4 ppm. LRMS *m/z* (ESI): 446.2 $(M+H)^+$. HRMS *m/z* (ESI): calculated for $C22H_{36}N_7O_3^+$ 446.2861, observed 446.2868.

Data for **7c** (0.0203 g, 7 %). ^{1}H NMR: (500 MHz, CD_3OD) δ_H 7.28 (t, J = 7.9 Hz, 1H, aryl 5'-H), 6.87 (d, J = 8.3 Hz, 1H, aryl 6'-H), 6.82 – 6.76 (m, 2H), 5.11 (s, 2H, CH_2Ar), 3.88 (t, J = 6.9 Hz, 2H, CH_2N), 3.79 (s, 3H, OCH_3), 3.50 (t, J = 6.8 Hz, 2H, CH_2N), 3.35 (t, J = 6.8 Hz, 2H, CH_2N), 1.64 (qui, J = 7.0 Hz, 2H), 1.37 – 1.25 (m, 10H), 0.90 (t, J = 6.8 Hz, 3H, CH_3) ppm. ^{13}C NMR: (125 MHz, CD_3OD) δ_C 161.6, 159.0, 157.1, 156.5, 152.5, 137.4, 131.1, 119.2, 114.0, 113.5, 55.7, 46.2, 43.4, 41.4, 41.3, 32.9, 30.4, 30.3, 28.7, 27.8, 23.7, 14.4 ppm. LRMS *m/z* (ESI): 446.3 $(M+H)^+$. HRMS *m/z* (ESI): calculated for $C_{22}H_{36}N_7O_3^+$ 446.2874, observed 446.2871.

Data for **7d** (0.142 g, 76%). ^{1}H NMR: (400 MHz, CD_3OD) δ_H 7.31 – 7.24 (m, 1H), 7.06 – 6.98 (m, 2H), 6.93 – 6.87 (m, 1H), 5.05 (s, 2H, CH_2Ar), 3.88 (s, 3H, OCH_3), 3.82 (t, J = 7.2 Hz, 2H, CH_2N), 3.48 (t, J = 6.8 Hz, 2H, CH_2N), 3.33 (t, J = 7.0 Hz, 2H, CH_2N), 1.59 (qui, J = 7.7 Hz, 2H), 1.36 – 1.17 (m, 10H), 0.85 (t, J = 6.7 Hz, 3H, CH_3) ppm. ^{13}C NMR: (100 MHz, CD_3OD) δ_C 159.0, 158.2, 157.2, 156.6, 152.5, 130.5, 128.0, 123.6, 122.0, 112.0, 56.2, 43.4, 42.6, 41.4, 41.3, 32.9, 30.3, 30.3, 28.7, 27.8, 23.7, 14.4 ppm. LRMS *m/z* (ESI): 446.3 $(M+H)^+$. HRMS *m/z* (ESI): calculated for $C_{22}H_{36}N_7O_3^+$ 446.2874, observed 446.2866.

Data for **7e** (0.0594 g, 25%). ^{1}H NMR: (400 MHz, CD_3OD) δ_H 7.34 – 7.26 (m, 2H), 7.14 – 7.05 (m, 2H), 5.12 (s, 2H, CH_2Ar), 3.87 (t, J = 7.4 Hz, 2H, CH_2N), 3.52 (t, J = 6.8 Hz, 2H, CH_2N), 3.37 (t, J = 6.7 Hz, 2H, CH_2N), 1.65 (qui, J = 7.3 Hz, 2H), 1.37 – 1.27 (m, 10H), 0.90 (t, J = 6.5 Hz, 3H, CH_3) ppm. ^{13}C NMR: (100 MHz, CD_3OD) δ_C 181.0, 165.0, 157.0, 154.2, 152.4, 132.0, 129.6, 116.7, 45.8, 43.4, 41.4, 41.3, 32.9, 30.3, 30.3, 28.7, 27.8, 23.7, 14.4 ppm. LRMS *m/z* (ESI): 434.2 $(M+H)^+$. HRMS *m/z* (ESI): calculated for $C_{21}H_{33}FN_7O_2^+$ 434.2674, observed 434.2668.

Data for **7f** (0.137 g, 57%). ^{1}H NMR: (400 MHz, d_6-DMSO) δ_H 7.40 (d, J = 8.6 Hz, 2H), 7.32 (d, J = 8.6 Hz, 2H), 5.13 (s, 2H, CH_2Ar), 3.69 (t, J = 7.5 Hz, 2H, CH_2N), 3.41 – 3.28 (m, 4H), 1.49 (qui, J = 7.3 Hz, 2H), 1.30 – 1.12 (m, 10H), 0.84 (t, J = 6.9 Hz, 3H, CH_3) ppm. ^{13}C NMR: (100 MHz, d_6-DMSO) δ_C 157.3, 153.9, 153.4, 150.9, 134.6, 132.0, 128.7, 128.5, 44.2, 41.4, 40.2, 39.3, 31.2, 28.7, 28.6, 27.3, 26.3, 22.1, 13.9 ppm. LRMS *m/z* (ESI): 450.2 $(M+H)^+$. HRMS *m/z* (ESI): calculated for $C_{21}H_{33}ClN_7O_2^+$ 450.2379, observed 450.2374.

Data for **7g** (0.112 g, 32 %). ^{1}H NMR: (400 MHz, CD_3OD) δ_H 7.53 (d, J = 8.6 Hz, 2H), 7.18 (d, J = 8.2 Hz, 2H), 5.10 (s, 2H, CH_2Ar), 3.87 (t, J = 7.7 Hz, 2H, CH_2N), 3.50 (t, J = 6.8 Hz, 2H, CH_2N), 3.35 (t, J = 7.0 Hz, 2H, CH_2N), 1.74 – 1.54 (m, 2H), 1.39 – 1.21 (m, 10H), 0.90 (t, J

= 6.7 Hz, 3H, CH_3) ppm. ^{13}C NMR: (100 MHz, CD_3OD) δ_C 163.5, 159.9, 155.4, 154.0, 135.9, 134.3, 133.0, 129.4, 45.9, 44.2, 41.4, 41.3, 32.9, 30.3, 27.8, 26.3, 25.9, 23.7, 14.4 ppm. LRMS *m/z* (ESI): 492.2 $(M-H)^-$. HRMS *m/z* (ESI): calculated for $C_{21}H_{33}BrN_7O_2^+$: 494.1874, observed 494.1870.

Data for **7h** (0.241 g, 85%). ^{1}H NMR: (400 MHz, CD_3OD) δ_H 8.24 (d, J = 8.9 Hz, 2H), 7.50 (d, J = 8.8 Hz, 2H), 5.26 (s, 2H, CH_2Ar), 3.87 (t, J = 7.7 Hz, 2H, CH_2N), 3.51 (t, J = 6.8 Hz, 2H, CH_2N), 3.37 (t, J = 6.8 Hz, 2H, CH_2N), 1.69 – 1.59 (m, 2H), 1.36 – 1.26 (m, 10H), 0.89 (t, J = 6.8 Hz, 3H, CH_3) ppm. ^{13}C NMR: (100 MHz, CD_3OD) δ_C 159.0, 157.0, 156.3, 152.4, 149.1, 143.6, 128.4, 124.9, 46.2, 43.5, 41.5, 41.2, 32.9, 30.7, 30.3, 28.7, 27.8, 23.7, 14.4 ppm. LRMS *m/z* (ESI):461.2 $(M+H)^+$. HRMS *m/z* (ESI): calculated for $C_{21}H_{33}N_8O_4^+$: 461.2619, observed 461.2616.

Data for **7j** (0.048 g, 10%). ^{1}H NMR: (400 MHz, CD_3OD) δ_H 7.68 (d, J = 8.0 Hz, 2H), 7.46 (d, J = 8.0 Hz, 2H), 5.24 (s, 2H, CH_2Ar), 3.87 (t, J = 7.4 Hz, 2H, CH_2N), 3.51 (t, J = 6.7 Hz, 2H, CH_2N), 3.38 (t, J = 6.5 Hz, 2H, CH_2N), 1.64 (qui, J = 7.1 Hz, 2H), 1.39 – 1.22 (m, 10H), 0.89 (t, J = 6.5 Hz, 3H, CH_3) ppm. ^{13}C NMR: (100 MHz, CD_3OD) δ_C 159.0, 157.0, 156.4, 152.4, 140.6, 130.8, 128.0, 126.8, 126.7, 46.2, 43.5, 41.4, 41.2, 32.9, 30.3, 30.3, 28.7, 27.8, 23.7, 14.4

ppm. LRMS *m/z* (ESI): 484.2 $(M+H)^+$. HRMS *m/z* (ESI): calculated for $C_{22}H_{33}F_3N_7O_2^+$ 484.2642, observed 484.2641.

Methods & spectroscopic data for preparation of analogues 8-14.

Preparation of N-methyl triazinedione amine compound **8a**. N-methylurea (0.72 g) was reacted with ethoxycarbonyl isothiocyanate using the method described above for compound **3**, to give the corresponding thiourea (1.184 g, 59%). Data: R_f 0.73 (1:1 EtOAc: pet. ether). ^{1}H NMR: (400 MHz, d_6-acetone) δ_H (rotamers observed) 11.56 (s, 1H, NH), 10.43 (s, 1H, NH), 9.20 (s, 1H, NH), 4.23 (2 x q, J = 7.1 Hz, 2H, OCH_2CH_3), 3.00 (2 x d, J = 4.7 Hz, 3H, NCH_3), 1.28 (2 x t, J = 7.1 Hz, 3H, CH_3) ppm. ^{13}C NMR: (100 MHz, d_6-acetone) δ_C (rotamers observed) 180.0, 156.5, 153.1, 63.7 & 62.8, 32.2 & 26.5, 14.5 & 14.3 ppm. LRMS *m/z* (ESI): 206.0 $(M+H)^+$, 228.0 $(M+Na)^+$. HRMS *m/z* (ESI): calculated for $C_6H_{11}N_3NaO_3S^+$ 228.0413, observed 228.0428. The thiourea (1.18 g) was cyclised with sodium methoxide as described above for compound **4**, to give the N-methyl triazinedione (0.87 g, 87%). Data: R_f 0.32 (1:1 EtOAc: pet. ether). ^{1}H NMR: (400 MHz, CD_3OD) δ_H 3.24 (s, 3H, CH_3N), 2.39 (s, 3H, SCH_3) ppm. ^{13}C NMR: (100 MHz, CD_3OD) δ_C 181.7, 160.5, 141.8, 27.6, 13.0 ppm. LRMS *m/z* (ESI): 174.0 $(M+H)^+$, 196.0 $(M+Na)^+$. HRMS *m/z* (ESI): calculated for $C_5H_7N_3NaO_2S^+$ 196.0151, observed 196.0173. This compound was then alkylated with benzyl bromide as described above for compounds **5a-g**, to give the benzylated triazinedione, which was isolated via method B as a yellow solid (0.566 g, 43%). Data: R_f 0.34 (3:7 EtOAc: pet. ether). ^{1}H NMR: (400 MHz, CD_3OD) δ_H 7.29 – 7.21 (m, 5H), 5.08 (s, 2H, CH_2Ar), 3.25 (s, 3H, CH_3N), 2.46 (s, 3H, SCH_3) ppm. ^{13}C NMR: (100 MHz, CD_3OD) δ_C 171.5, 154.2, 151.1, 135.3, 128.6, 128.3, 127.2, 48.7, 28.7, 14.7 ppm. LRMS *m/z* (ESI): 264.0 $(M+H)^+$, 286.0 $(M+Na)^+$. HRMS *m/z* (ESI): calculated for $C_{12}H_{13}N_3NaO_2S^+$ 286.0621, observed 286.0623. This compound was then reacted with ethylenediamine as described above for compounds **6a-g**, to give compound **8a** (0.510 g, 86%). Data: ^{1}H NMR: (400 MHz, CD_3OD) δ_H 7.41 – 7.16 (m, 5H), 5.19 (s, 2H, CH_2Ar), 3.40 (t, J = 6.3 Hz, 2H, CH_2N), 3.27 (s, 3H, CH_3N), 2.69 (t, J = 6.3 Hz, 2H, CH_2N) ppm. ^{13}C NMR: (100 MHz, CD_3OD) δ_C 155.1, 154.9, 152.3, 135.3, 129.0, 128.0, 126.6, 45.3, 44.2, 40.5, 28.6 ppm. LRMS *m/z* (ESI): 276.1 $(M+H)^+$. HRMS *m/z* (ESI): calculated for $C_{13}H_{18}N_5O_2^+$ 276.1455, observed 276.1469.

Compound **8a** was then converted using the method described above for compounds **7a-g** to the corresponding guanidine **8b** (0.046 g, 16%). ^{1}H NMR: (400 MHz, CD_3OD) δ_H 7.39 – 7.21 (m, 5H), 5.18 (s, 2H, CH_2Ar), 3.53 (t, J = 6.6 Hz, 2H, CH_2N), 3.36 (t, J = 6.6

Hz, 2H, CH_2N), 3.30 (s, 3H, CH_3N) ppm. ^{13}C NMR: (100 MHz, CD_3OD) δ_C 159.7, 156.3, 154.4, 153.9, 136.2, 129.9, 128.9, 127.5, 46.4, 41.4, 41.1, 29.3 ppm. LRMS *m/z* (ESI): 318.1 $(M+H)^+$. HRMS *m/z* (ESI): calculated for $C_{14}H_{20}N_7O_2^+$ 318.1673, observed 318.1674.

Preparation of unbenzylated triazinedione amine **9a**. Compound **4** (1.50 g) was reacted with ethylenediamine as described above for compounds **6a-g** to give compound **9a** (1.38 g, 88%). ^1H NMR: (400 MHz, CD_3OD) δ_H 3.79 (t, J = 7.5 Hz, 2H, CH_2N), 3.34 (t, J = 6.1 Hz, 2H, CH_2N), 2.76 (t, J = 6.3 Hz, 2H, CH_2N), 1.66 – 1.52 (m, 2H), 1.38 – 1.18 (m, 10H), 0.89 (t, J = 6.8 Hz, 3H, CH_3) ppm. ^{13}C NMR: (100 MHz, CD_3OD) δ_C 166.4, 156.2, 152.9, 43.3, 41.7, 41.3, 32.3, 29.9, 29.7, 28.7, 27.4, 23.0, 13.8 ppm. LRMS *m/z* (ESI): 306.2 $(M+Na)^+$. HRMS *m/z* (ESI): calculated for $C_{13}H_{25}N_5NaO_2^+$ 306.1900, observed 306.1897.
Compound **9a** was then converted using the method described above for compounds **7a-g** to the corresponding guanidine **9b.** In this case no precipitate was formed at the end of the reaction, so the acetonitrile was removed under reduced pressure, and ethyl acetate added, whereupon a white precipitate was formed, which was collected by filtration. Data for compound **9b** (0.058 g, 7%). ^1H NMR: (400 MHz, CD_3OD) δ_H 3.79 (t, J = 7.6 Hz, 2H, CH_2N), 3.43 (t, J = 7.0 Hz, 2H, CH_2N), 2.94 (t, J = 7.0 Hz, 2H, CH_2N), 1.64 – 1.54 (m, 2H), 1.37 – 1.26 (m, 10H), 0.89 (t, J = 6.3 Hz, 3H, CH_3). LRMS *m/z* (ESI): 326.3 $(M+H)^+$. HRMS *m/z* (ESI): calculated for $C_{14}H_{28}N_7O_2^+$: 326.2299, observed 326.2294.

Preparation of *N*-ethyl analogue **10**. Compound **5a** (0.63 g) was dissolved in anhydrous methanol (30 mL) and the solution was stirred under nitrogen. Ethylamine (2M in MeOH) (4.31 mL, 5 eq.) was added, and the resulting solution was stirred at room temperature for 48 h. Upon completion of the reaction by TLC, the methanol was removed under reduced pressure, and the residue was resuspended in ethyl acetate (80 mL) and transferred to a separatory funnel. The organic fraction was washed with distilled water (2 x 80 mL) and sat. sodium chloride solution (80 mL), dried ($MgSO_4$) and evaporated at reduced pressure to give compound **10** as a white solid (0.48 g, 77%). R_f 0.08 (9:1 EtOAc: MeOH). ^1H NMR: (400 MHz, d_6-acetone) δ_H 7.39 – 7.24 (m, 5H), 5.22 (s, 2H, CH_2Ar), 3.83 (t, J = 7.2 Hz, 2H, CH_2N), 3.38 (q, J = 7.2 Hz, 2H, NCH_2), 1.62 (qui, J = 7.3 Hz, 2H), 1.39 – 1.23 (m, 10H), 1.06 (t, J = 7.2 Hz, 3H, $NCH_2C\underline{H}_3$), 0.88 (t, J = 6.9 Hz, 3H, CH_3) ppm. ^{13}C NMR: (100 MHz, d_6-acetone) δ_C 154.8, 154.7, 152.5, 136.5, 129.5, 128.4, 127.3, 45.3, 42.5, 37.3, 32.5, 30.1, 30.0, 28.6, 27.6, 23.3, 14.6, 14.3 ppm. LRMS *m/z* (ESI): 359.2 $(M+H)^+$, 381.2 $(M+Na)^+$. HRMS *m/z* (ESI): calculated for $C_{20}H_{30}N_4NaO_2^+$ 381.2261, observed 381.2258.

Preparation of *N*-(2'-hydroxyethyl) analogue **11**. Compound **5a** (0.500 g) was dissolved in anhydrous toluene (30 mL) and the solution was stirred under nitrogen. Ethanolamine (0.422 g, 0.42 mL, 5 eq.) was added, and the reaction was heated to reflux (110 °C) for 18 h. Upon completion of the reaction by TLC, the reaction mixture was cooled to room temperature, and the toluene was removed under reduced pressure. The resulting light brown residue was resuspended in ethyl acetate (40 mL), and the organic layer was washed with distilled water (2 x 30 mL) and saturated sodium chloride solution (30 mL), dried ($MgSO_4$) and evaporated under reduced pressure to give compound **11** as an off-white solid (0.3201 g, 62%). ^1H NMR: (400 MHz, CD_3OD) δ_H 7.41 – 7.22 (m, 5H), 5.15 (s, 2H, CH_2Ar), 3.86 (t, J = 7.5 Hz, 2H, CH_2N), 3.62 (td, J = 5.7, 3.8 Hz, 2H, CH_2OH), 3.48 (t, J = 5.8 Hz, 2H, $NC\underline{H_2}CH_2OH$), 1.69 – 1.55 (m, 2H), 1.39 – 1.26 (m, 10H), 0.90 (t, J = 6.6 Hz, 3H, CH3) ppm. ^{13}C NMR: (100 MHz, CD_3OD) δ_C 157.3, 156.2, 152.8, 136.2, 129.9, 128.9, 127.5, 60.9, 46.2, 45.0, 43.3, 32.9, 30.4, 30.3, 28.8, 27.8, 23.7, 14.4 ppm. LRMS *m/z* (ESI): 375.2 $(M+H)^+$, 397.2 (M+Na)+. HRMS *m/z* (ESI): calculated for $C_{20}H_{30}N_4NaO_3^+$ 397.2210, observed 397.2209.

Preparation of urea sidechain analogue **12**. Compound **6a** (0.70 g) was dissolved in ethanol (20 mL) and heated to 60 °C with stirring. Concentrated hydrochloric acid (0.1 mL, 1.87 $x10^{-3}$ mol, 1 eq.) was added and the resulting solution was stirred for 15 minutes. A solution of potassium cyanate (0.61 g, 7.50 $x10^{-3}$ mol, 4 eq.) in water (20 mL) was added after 15 minutes. The solution was allowed to cool to room temperature and stirred for 48 h. The resulting precipitate was collected via vacuum filtration to afford compound **12** as a pale pink solid (0.067 g, 9%). ^1H NMR: (400 MHz, d_6-DMSO) δ_H 7.38 – 7.22 (m, 5H), 5.14 (s, 2H, CH_2Ar), 3.70 (t, J = 7.3 Hz, 2H, CH_2N), 3.51 (t, J = 6.5 Hz, 2H, CH_2N), 2.97 (t, J = 6.5 Hz, 2H, CH_2N), 1.49 (qui, J = 7.2 Hz, 2H), 1.29 – 1.14 (m, 10H), 0.82 (t, J = 6.6 Hz, 3H, CH_3) ppm. ^{13}C NMR: (100 MHz, d_6-DMSO) δ_C 164.0, 154.5, 153.9, 151.3, 135.6, 128.8, 127.6, 126.6, 44.9, 41.6, 39.0, 38.3, 31.4, 28.9, 28.8, 27.5, 26.4, 22.3, 14.1 ppm. LRMS *m/z* (ESI): 439.3 $(M+Na)^+$.

Preparation of bis-aryl substituted analogue **13**. 1,3-Diphenyl-1-propanol was prepared was prepared by catalytic hydrogenation of chalcone, followed by reduction with sodium borohydride.[S1] 1,3-Diphenyl-1-propanol was then converted to 1,3-diphenyl-1-chloropropane using thionyl chloride.[S2] 1,3-diphenyl-1-chloropropane (0.445 g), compound **4** (1.05 g, 3.86 $x10^{-3}$ mol, 2 eq.) and potassium carbonate (1.07 g, 7.71 $x10^{-3}$ mol, 4 eq.) were dissolved in DMF (20 mL) and stirred under nitrogen at room temperature for 120 h. Upon completion of

the reaction by TLC, distilled water (100 mL) was added, but no precipitate was formed. The product was extracted into ethyl acetate (2 x 80 mL). The combined organic layers were washed with saturated sodium chloride solution (100 mL), dried ($MgSO_4$) and evaporated under reduced pressure to give the alkylated triazinedione (0.023 g). R_f 0.37 (1:1 Petroleum ether: EtOAc). ^{1}H NMR: (400 MHz, d_6-acetone) δ_H 7.38 – 7.31 (m, 10H), 5.35 (t, J = 6.8 Hz, 1H, CHAr), 3.80 (t, J = 6.9 Hz, 2H, CH_2N), 2.63 (t, J = 6.4 Hz, 2H, CH_2Ar), 2.46 (s, 3H, SCH_3), 1.96 – 1.92 (m, 2H), 1.65 – 1.56 (m, 2H), 1.37 – 1.23 (m, 10H), 0.88 (t, J = 6.8 Hz, 3H, CH_3) ppm. ^{13}C NMR: (100 MHz, d_6-acetone) δ_C 161.5, 154.1, 151.4, 142.0, 141.0, 129.5, 129.1, 127.9, 127.6, 126.6, 126.4, 47.5, 42.7, 40.1, 32.7, 32.0, 30.0, 29.9, 28.3, 27.6, 23.2, 14.3, 13.2 ppm. LRMS *m/z* (ESI): 466.2 $(M+H)^+$, 488.2 $(M+Na)^+$. HRMS *m/z* (ESI): calculated for $C_{27}H_{35}N_3NaO_2S^+$ 488.2347, observed 488.2336.

The alkylated triazinedione (0.023 g) was reacted with ethylenediamine using the method described above for compounds **6a-g**, to give compound **13** (0.0202 g). ^{1}H NMR: (400 MHz, d_6-acetone) δ_H 7.39 – 7.31 (m, 10H), 5.34 (t, J = 6.8 Hz, 1H, CHAr), 3.82 (t, J = 6.6 Hz, 2H, CH_2N), 3.72 (t, J = 7.2 Hz, 2H, CH_2N), 2.79 (t, J = 7.3 Hz, 2H, CH_2N), 2.69 – 2.63 (m, 2H), 2.13 – 2.08 (m, 2H), 1.70 – 1.59 (m, 2H), 1.36 – 1.32 (m, 10H), 0.89 (t, J = 6.9 Hz, 3H, CH_3) ppm. ^{13}C NMR: (100 MHz, d_6-acetone) δ_C 157.5, 154.5, 150.3, 143.0, 141.1, 129.9, 129.4, 128.4, 127.7, 127.3, 126.8, 49.8, 43.4, 42.7, 40.2, 39.9, 32.5, 32.1, 30.4, 29.9, 28.6, 27.6, 23.3, 14.4 ppm. LRMS *m/z* (ESI): 478.3 $(M+H)^+$. HRMS *m/z* (ESI): calculated for $C_{28}H_{40}N_5O_2^+$ 478.3177, observed 478.3172.

Preparation of substituted triazine analogues **14a** and **14b** was achieved using a modification of the method of Srivastava *et al* .[S3]

To a solution of cyanuric chloride (5g, 0.0271mol), in tetrahydrofuran (50mL), 1 eq. of octylamine (4.48ml, 0.0271mol) was added. The reaction mixture was stirred on ice, with dropwise addition of potassium carbonate solution (5% v/v) to maintain pH >9 for 4 hr, then stirred overnight. After completion of the reaction was observed via TLC (eluent EtOAc/petroleum ether 1.5:8.5), benzylamine (2.96mL, 0.0271mol) was added to the reaction mixture, with dropwise addition of potassium carbonate solution (5% v/v) to maintain pH >9 for 4 hr, then stirred overnight, and completion of the reaction was again observed by TLC. A white precipitate was formed, which was collected under vacuum and washed with water. The white solid was purified via recrystallisation from hot ethyl acetate, to give the 3,5-disubstituted 1-chlorotriazine in 78% yield. Data: M.p = 182 – 186 ^{0}C; δ_H (300 MHz, $CDCl_3$) 7.31 (5H, s), 4.62 (1H, d, J = 7.0 Hz, Ar-C$\underline{H_2}$NH-), 3.50 – 3.23 (2H, m, -NHC$\underline{H_2}$), 1.58 – 1.50 (2H, m), 1.32

– 1.30 (10H, m), 0.88 (3H, t, J = 6.7, CH_3) ppm; δ_C (75 MHz, $CDCl_3$) quaternary C not observed, 129.7, 128.6, 127.5, 44.9, 41.0, 31.8, 30.0, 29.9, 29.2, 26.8, 22.6, 14.1 ppm. LRMS (ESI) *m/z*: 370.2 = $(M+Na)^+$.

The 1-chlorotriazine intermediate (1.1g, 0.00431mol) was refluxed with 5 eq. of ethylene diamine (1.06mL, 0.0158mol) in toluene (25mL) for 1 hour. Completion of reaction was monitored by TLC (eluent DCM/MeOH 9:1). After completion of reaction, the resulting solution was allowed to cool to room temperature, and was then filtered to remove an oily residue. Ethyl acetate (35mL) was added, and the organic layer was washed twice with distilled water (50mL x 2) and twice with saturated sodium chloride solution (50ml x 2), then dried ($MgSO_4$), and the solvent removed under reduced pressure, to give compound **14a** as an oil (77% yield). Data: δ_H (400 MHz, CD_3OD) 7.28 – 7.09 (5H, m), 4.52 (2H, s ArC$\underline{H_2}$NH-), 3.40 (2H, m, CH_2N), 3.32 (2H, m, CH_2N), 2.76 (2H, m, CH_2N), 1.56 – 1.50 (2H, m), 1.32 – 1.24 (10H, m), 0.89 (3H, t, J = 6.1, CH_3) ppm; δ_C (100 MHz, CD_3OD) quaternary C not observed, 129.4, 127.3, 126.4, 43.8, 42.6, 41.1, 40.1, 31.7, 31.6, 29.5, 29.1, 26.7, 22.4, 13.1 ppm. LRMS (ESI) *m/z*: 372.3 = $(M+H)^+$, 394.3 = $(M+Na)^+$.

To a solution of **14a** (0.9026g, 0.00243mol) in acetonitrile (25mL), 1-H-Pyrazole-1-carboxamidine hydrochloride (0.3561g, 0.00243mol, 1 eq) and di-isopropylethylamine (0.42ml, 0.00243mol, 1 eq) were added. The reaction mixture was stirred for 24 hours at room temperature. After completion of the reaction by TLC (eluent DCM/MeOH 9:1), a white precipitate was isolated via filtration, to give **14b** as a white solid (70% yield). Data: M.p = 69 – 73 ^{0}C. δ_H (400 MHz, CD_3OD) 7.29 - 7.20 (5H, m), 4.54 (2H, s, ArC$\underline{H_2}$NH-), 3.45 (2H, m, CH_2N), 3.37 (4H, s, 2 x CH_2N), 1.56 – 1.50 (2H, m), 1.32 – 1.24 (10H, m), 0.89 (3H, t, J = 5.7 Hz, CH_3) ppm; δ_C (100 MHz, CD_3OD) quaternary C not observed, 129.6, 127.9, 126.8, 43.8, 42.6, 41.1, 40.1, 31.7, 31.6, 29.5, 29.1, 26.6, 22.3, 13.1 ppm; LRMS (ESI) *m/z*: 414.3 = $(M+H)^+$, 436.4 = $(M+Na)^+$.

References for Supporting Information

S1. A.I. Vogel. Vogel's textbook of practical organic chemistry, 5th edition, Longman, 1989, p. 523-524 & 1034-1035.

S2. W.E. Bissinger and F.E. Kung. A study of the reaction of alcohols with thionyl chloride. *J. Am. Chem. Soc.,* 1947, **69**, 2158-2163.

S3. J. K. Srivastava, P. Dubey, S. Singh, H. R. Bhat, M. K. Kumawat and U. P. Singh. Discovery of novel 1,3,5-triazine-thiazolidine-2,4-diones as dipeptidyl peptidase-4 inhibitors

with antibacterial activity targeting the S1 pocket for the treatment of type 2 diabetes. *RSC Advances*, 2015, **5**, 14095–14102.

^{1}H and ^{13}C NMR spectra for final compounds

6a Amine R = H ^{1}H NMR

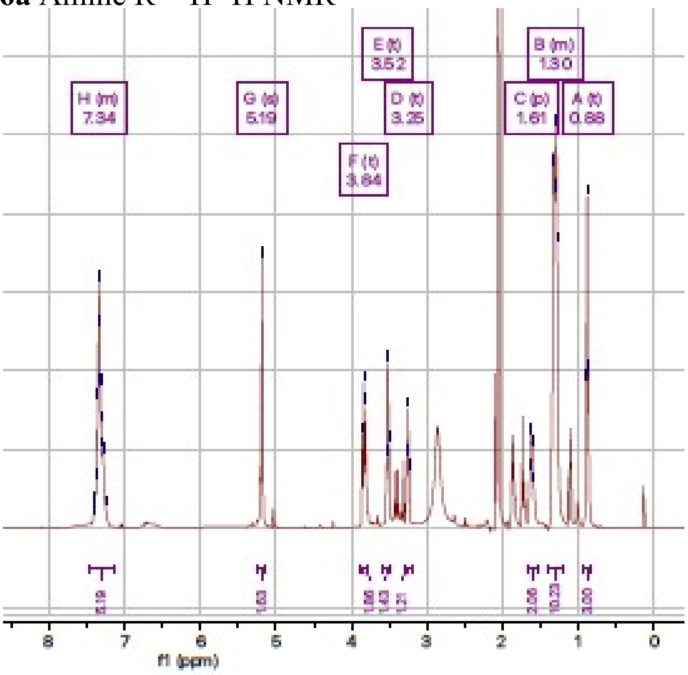

^{13}C NMR

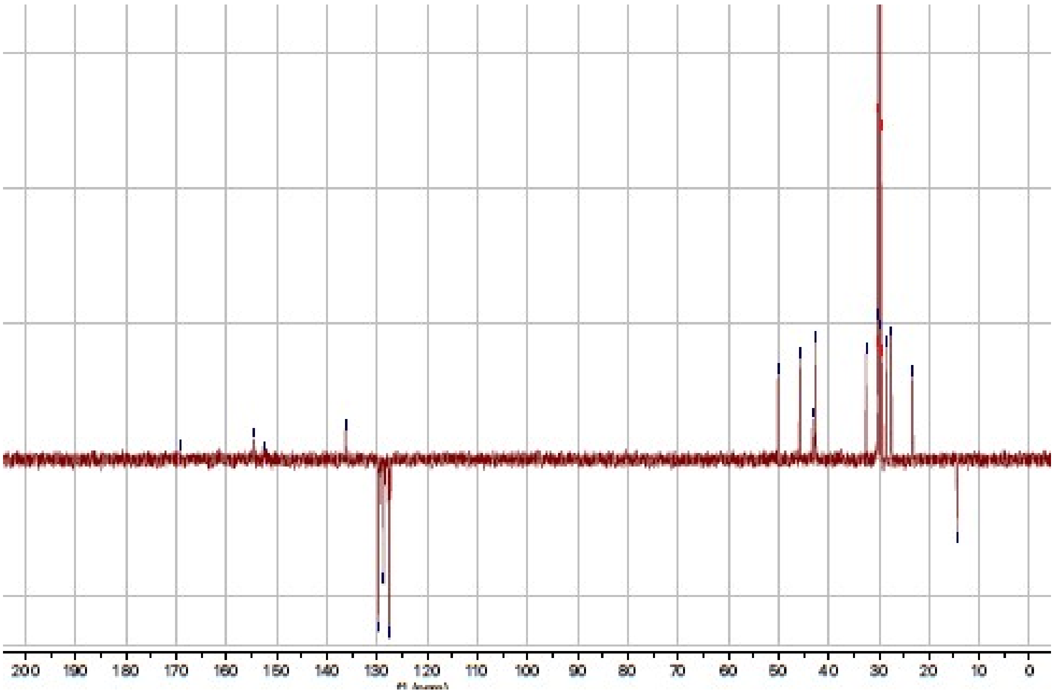
200 190 180 170 160 150 140 130 120 110 100 90 80 70 60 50 40 30 20 10 0

7a Guanidine R=H ^{1}H NMR

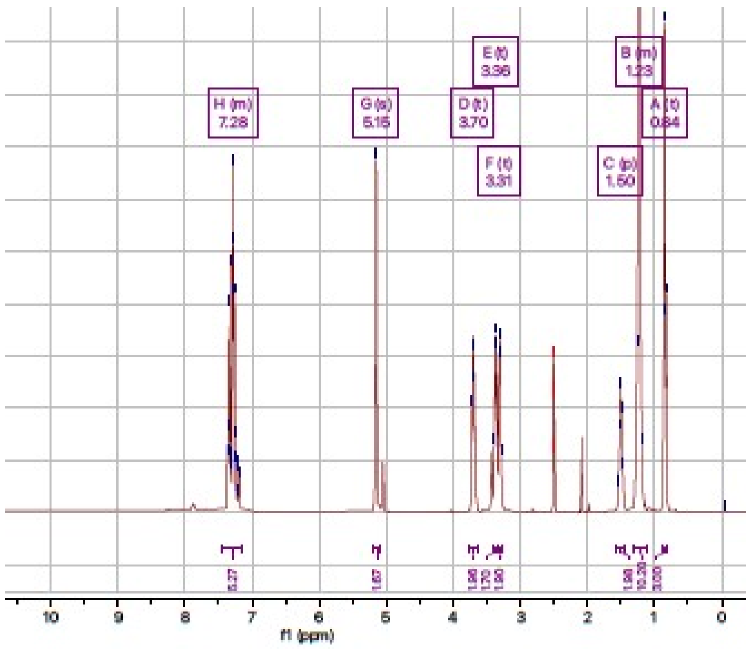

^{13}C NMR

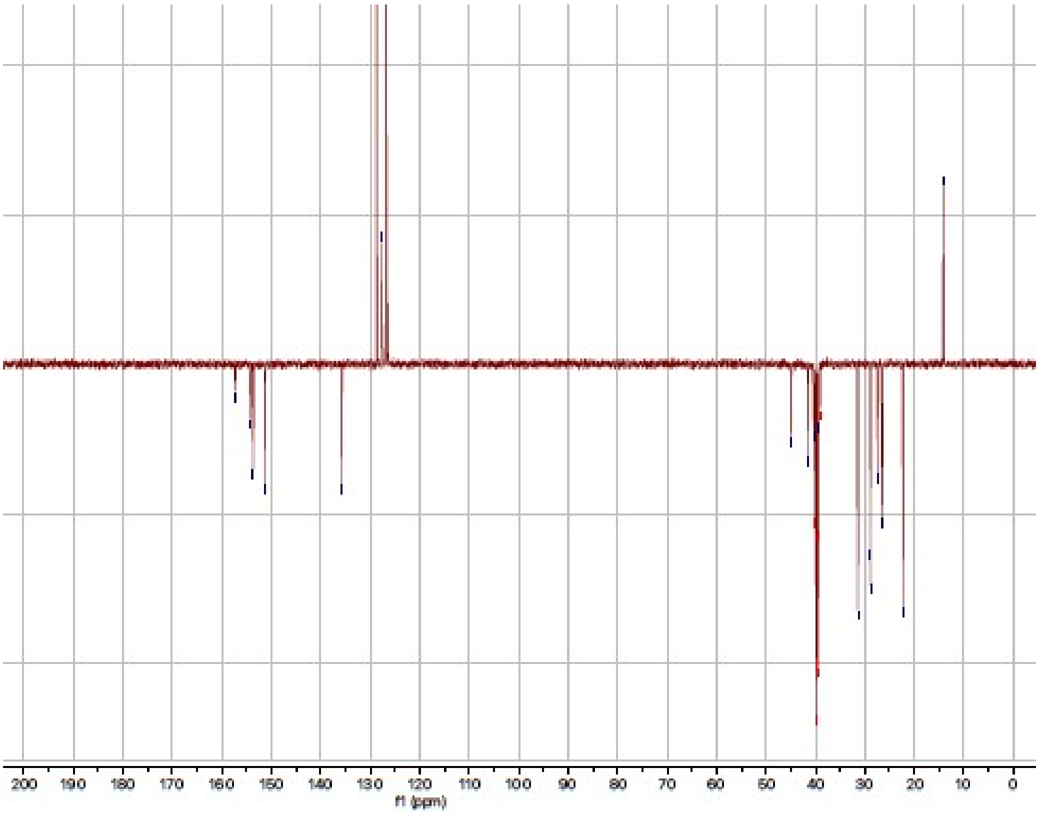
200 190 180 170 160 150 140 130 120 110 100 90 80 70 60 50 40 30 20 10 0
f1 (ppm)

6b Amine R = 4-OCH[illegible] ^{1}H NMR

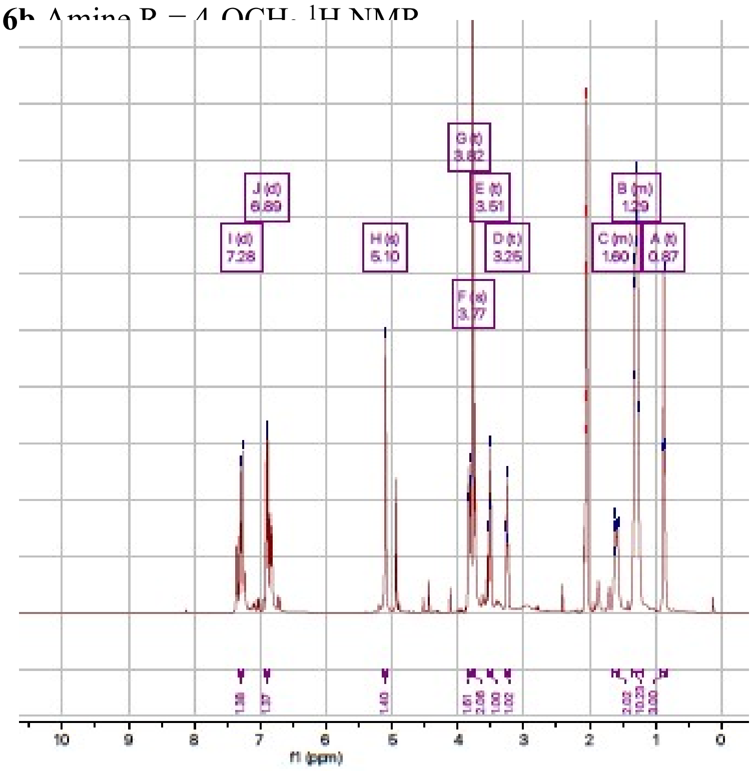

^{13}C NMR

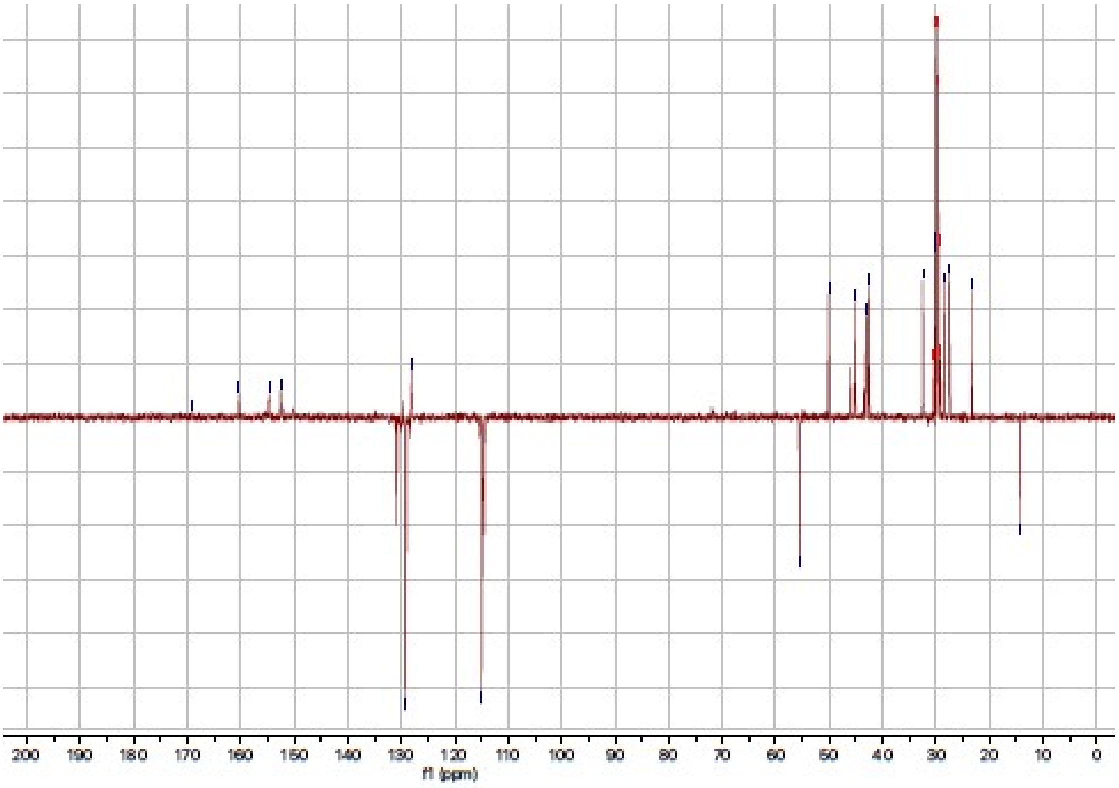
200
190
180
170
160
150
140
130
120
110
100
90
80
70
60
50
40
30
20
10
0
f1 (ppm)

7b Guanidine R = 4-OCH_3 ^1H NMR

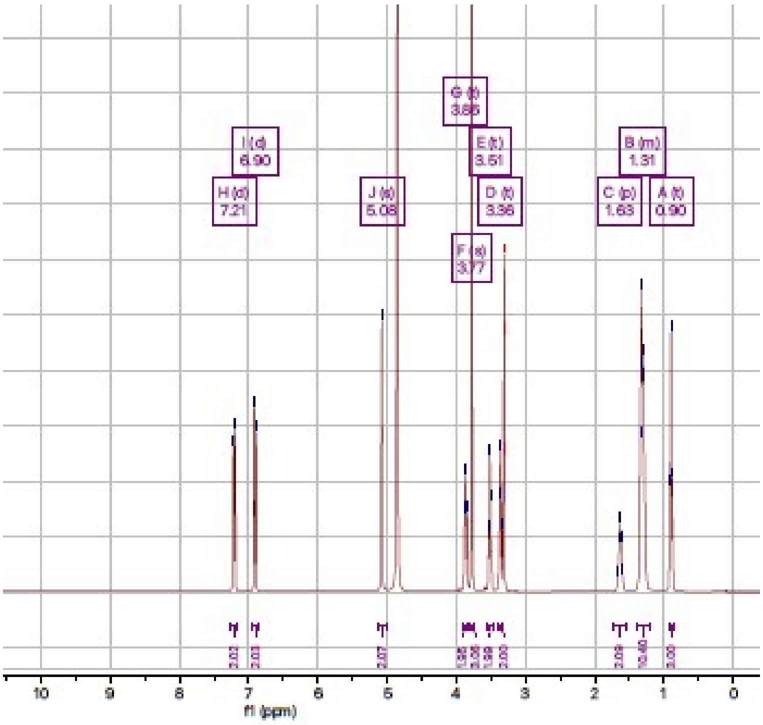

^{13}C NMR

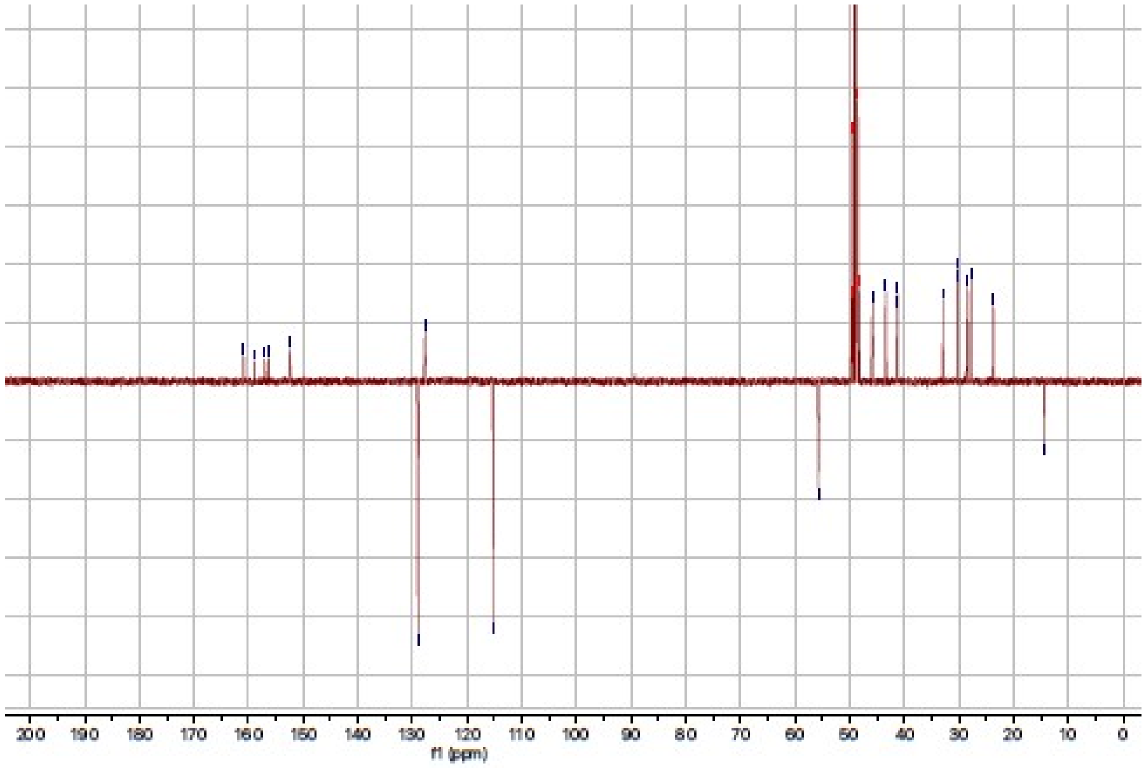

200
190
180
170
160
150
140
130
120
110
100
90
80
70
60
50
40
30
20
10
0
f1 (ppm)

6c A

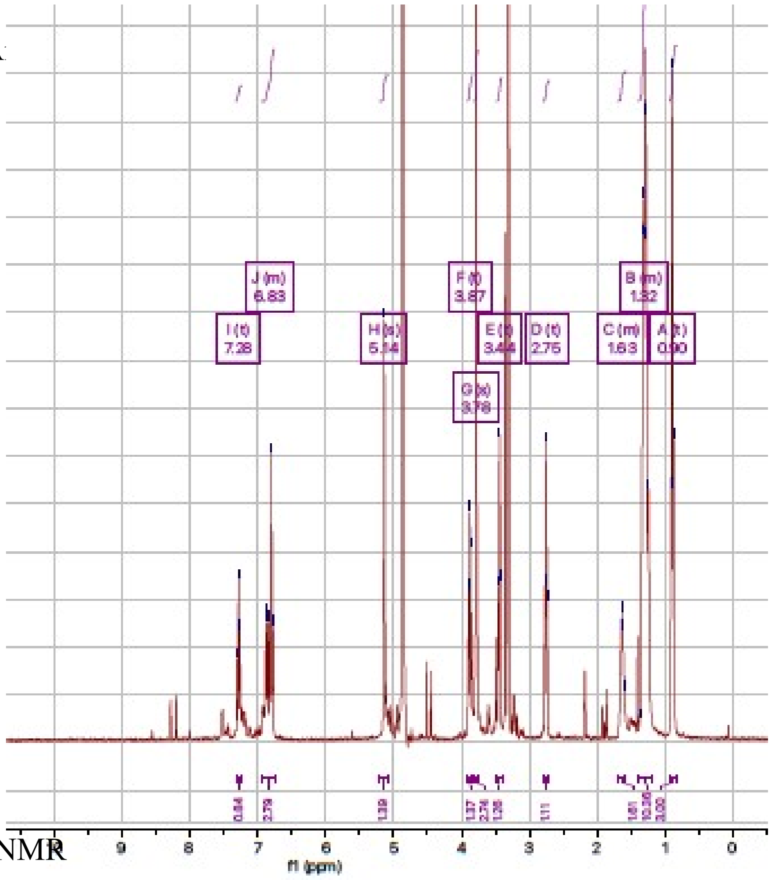
J (m)
6.83
I (t)
7.28
H (s)
5.14
F (t)
3.87
E (t)
3.44
G (s)
3.78
D (t)
2.75
B (m)
1.32
C (m)
1.63
A (t)
0.90
9
8
7
6
5
4
3
2
1
0
f1 (ppm)

^{13}C NMR

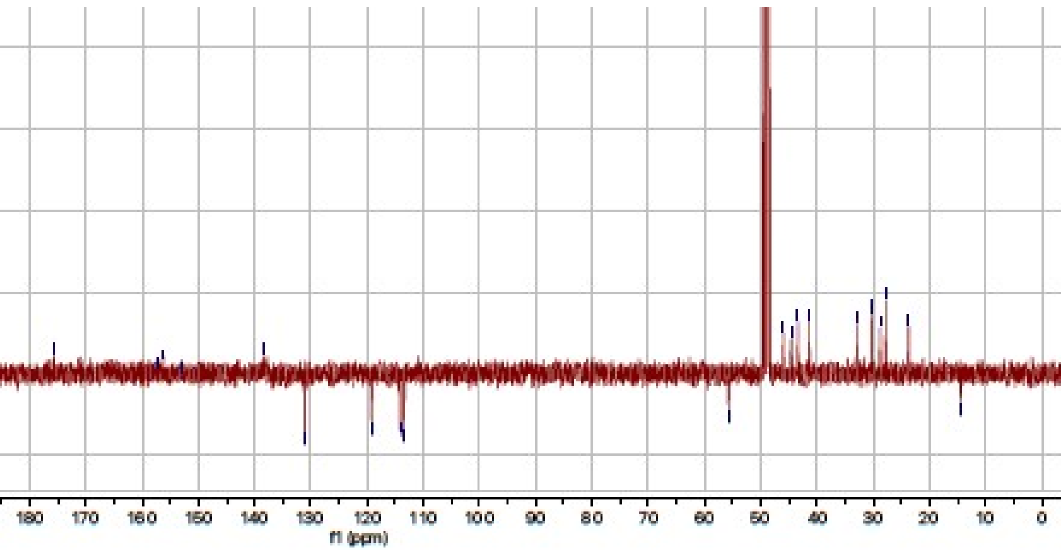
180 170 160 150 140 130 120 110 100 90 80 70 60 50 40 30 20 10 0
f1 (ppm)

7c Guanidine R = 3-OCH_3 ^1H NMR

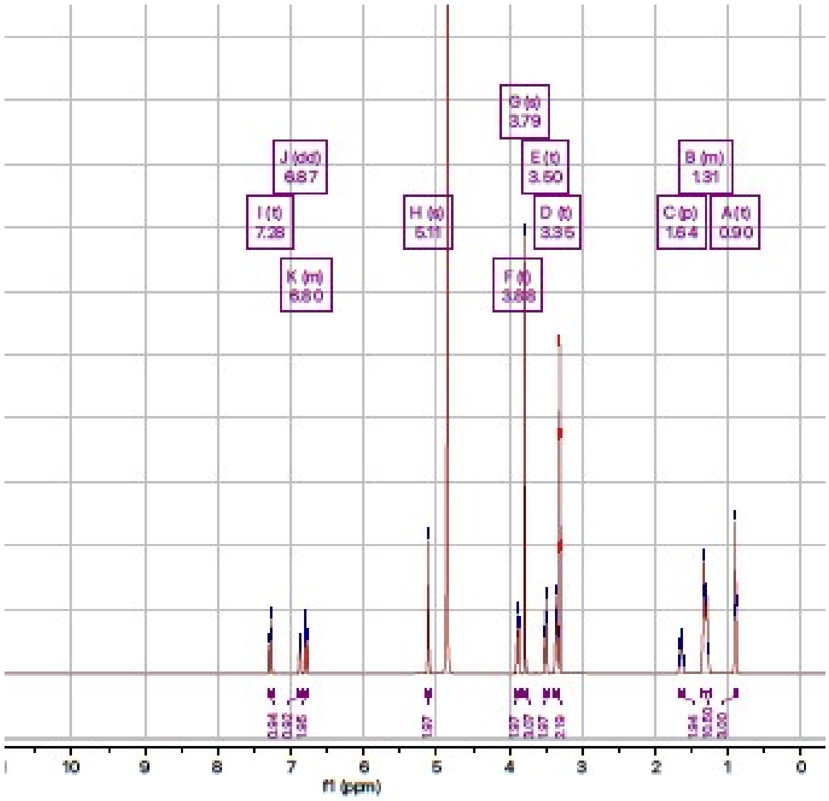

^{13}C NMR

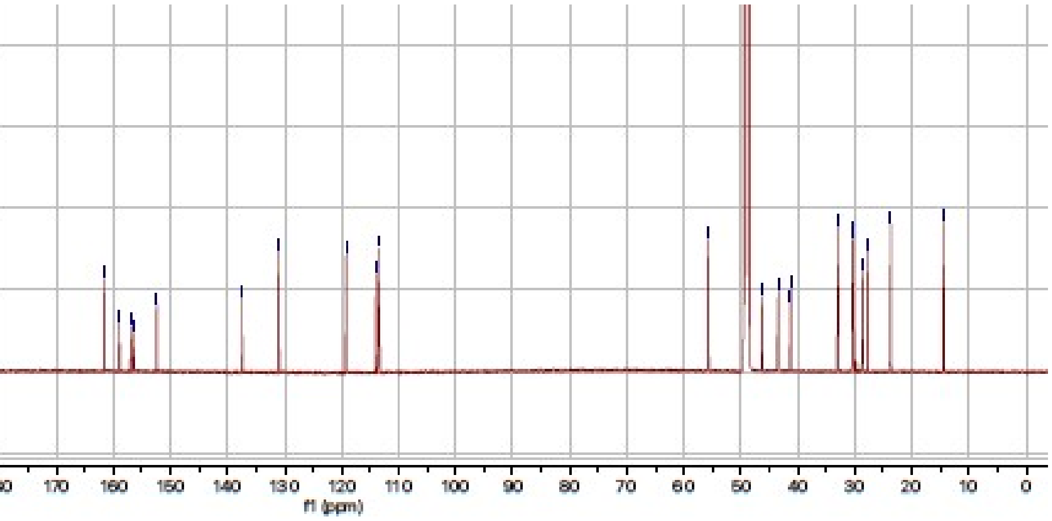
170
160
150
140
130
120
110
100
90
80
70
60
50
40
30
20
10
0
f1 (ppm)

6d Amine R = 2-OCH_3 ^1H NMR

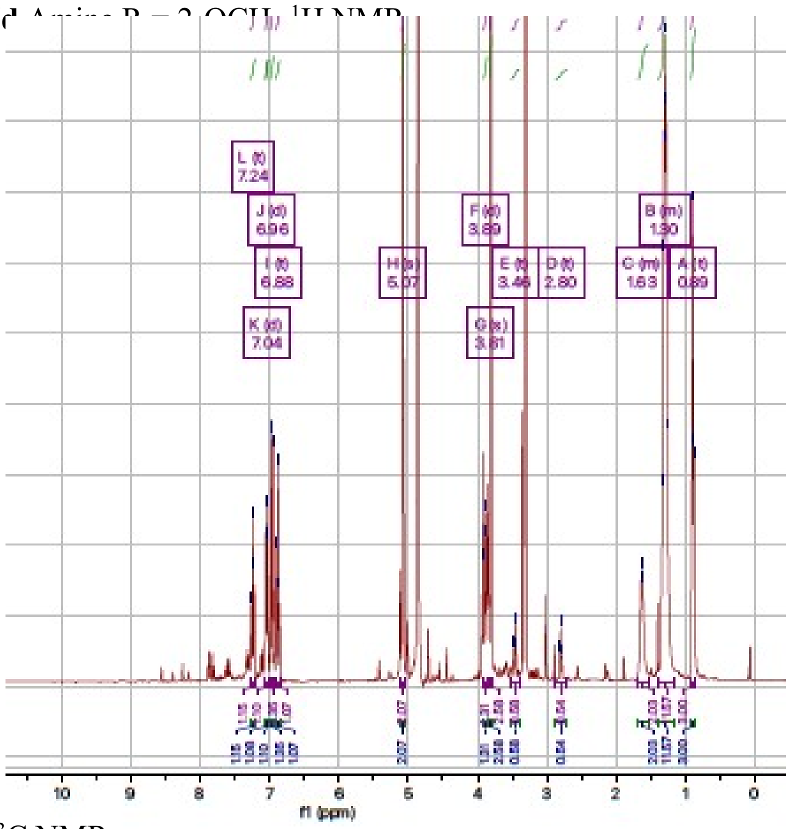

^{13}C NMR

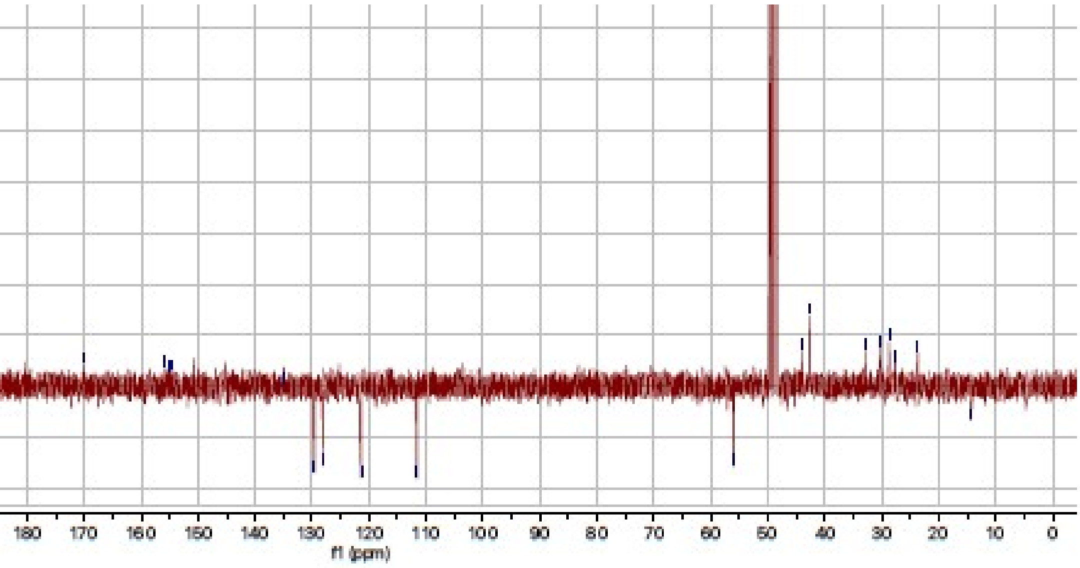
180 170 160 150 140 130 120 110 100 90 80 70 60 50 40 30 20 10 0
f1 (ppm)

7d C

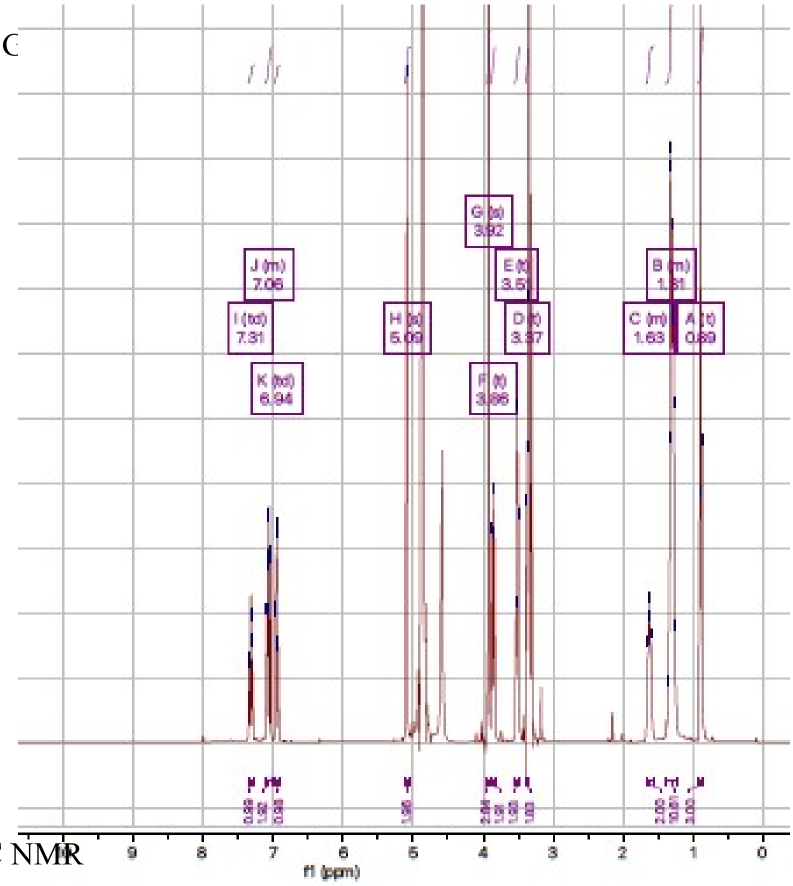

^{13}C NMR

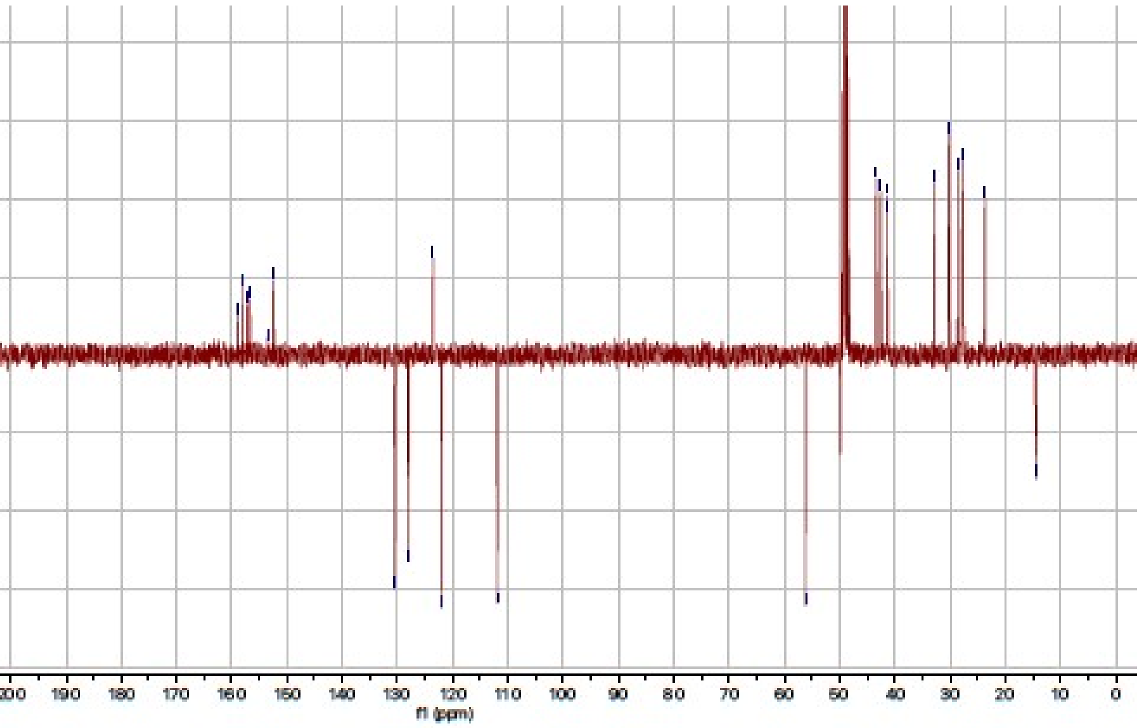

200 190 180 170 160 150 140 130 120 110 100 90 80 70 60 50 40 30 20 10 0
f1 (ppm)

6e Amine R = 4-F ^{1}H NMR

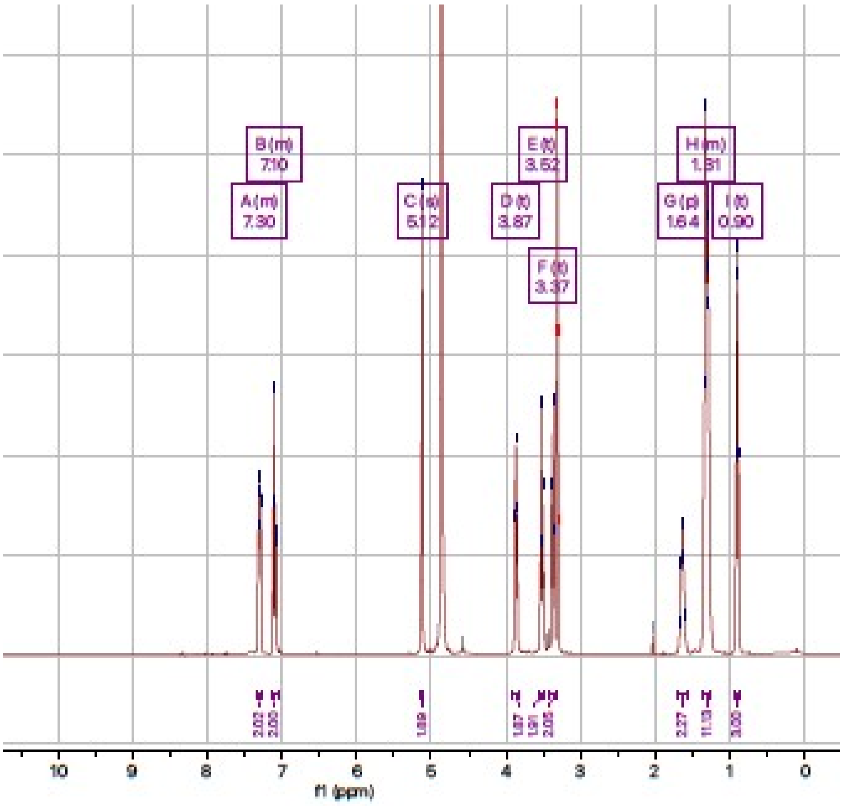

^{13}C NMR

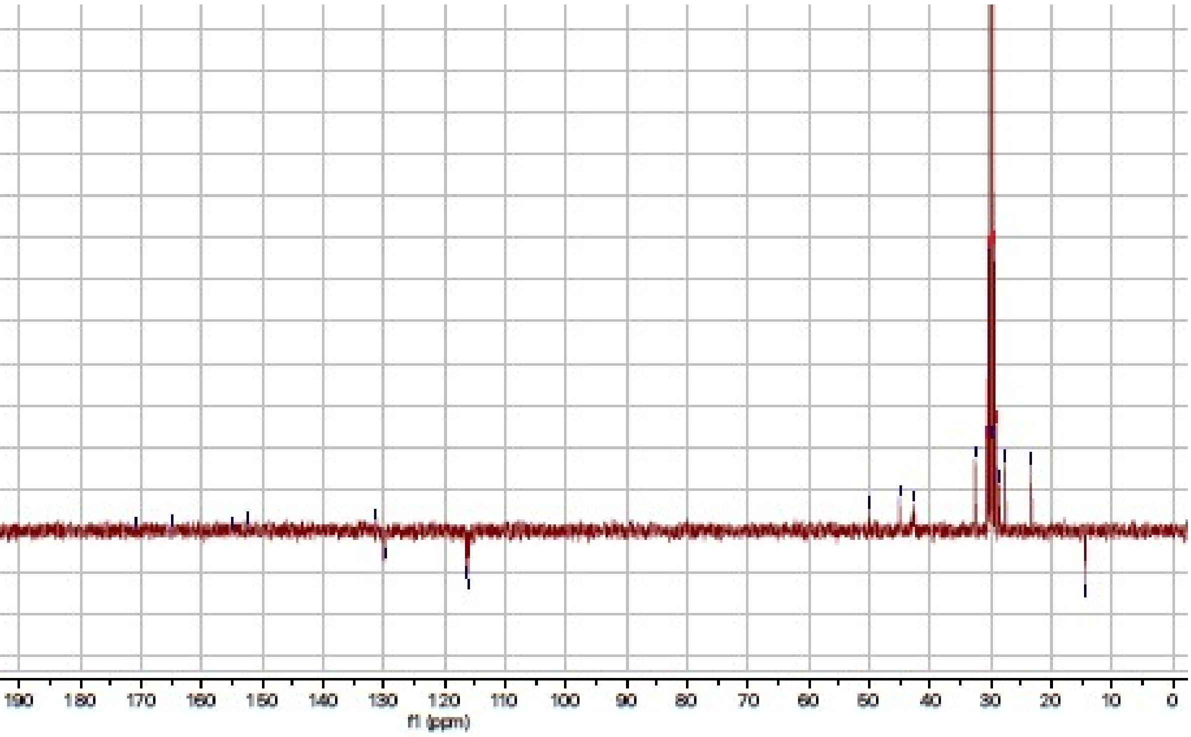
190 180 170 160 150 140 130 120 110 100 90 80 70 60 50 40 30 20 10 0
f1 (ppm)

7e Guanidine R = 4-F ^{1}H NMR

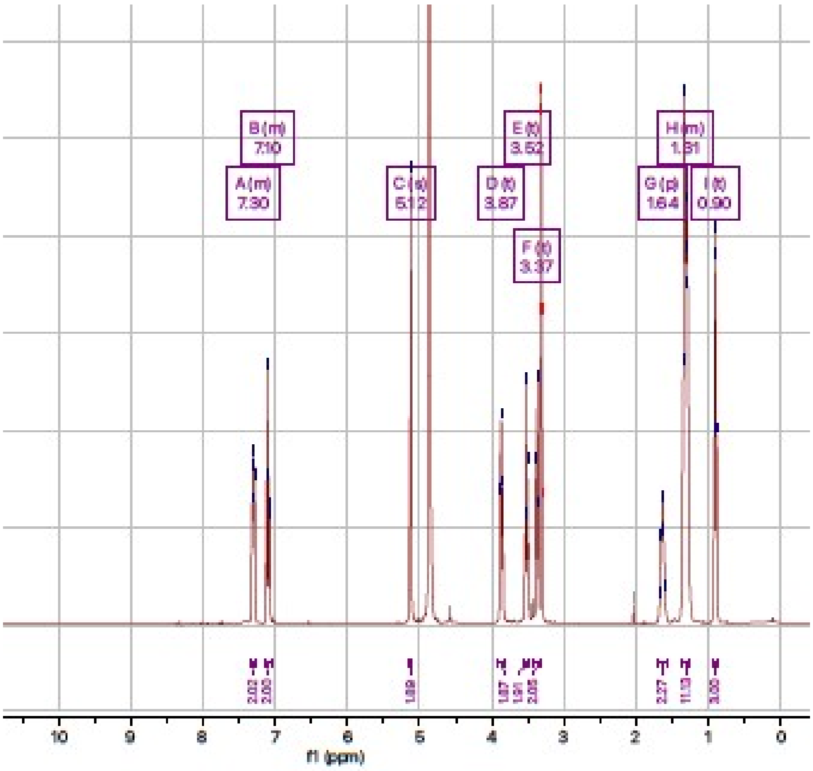

^{13}C NMR

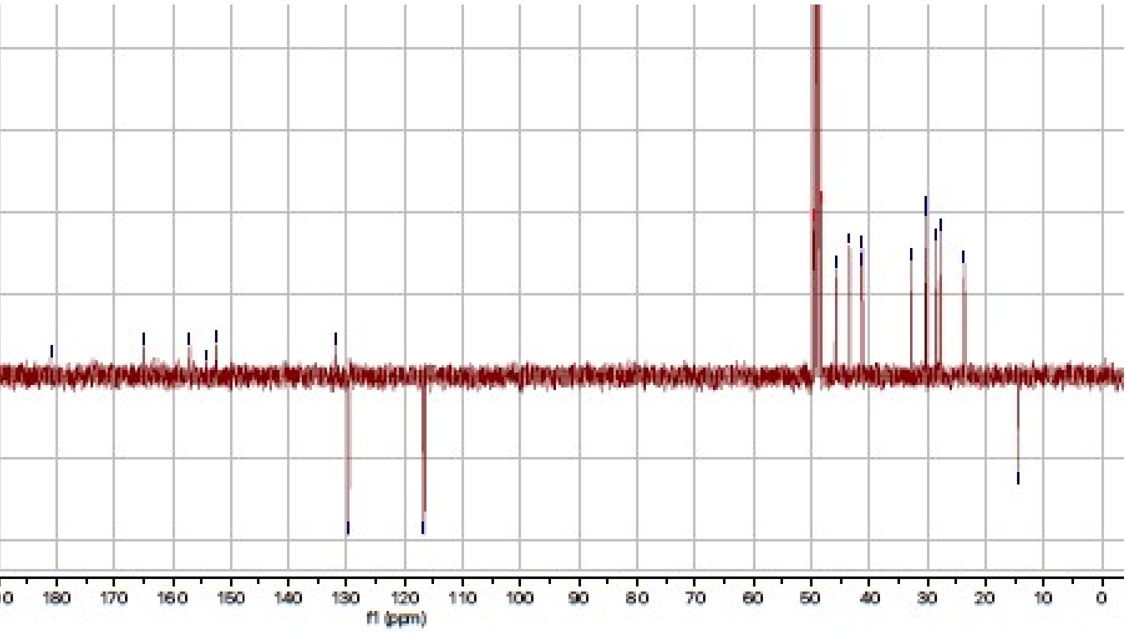
f1 (ppm)

6f Amine R = 4-Cl ¹H NMR

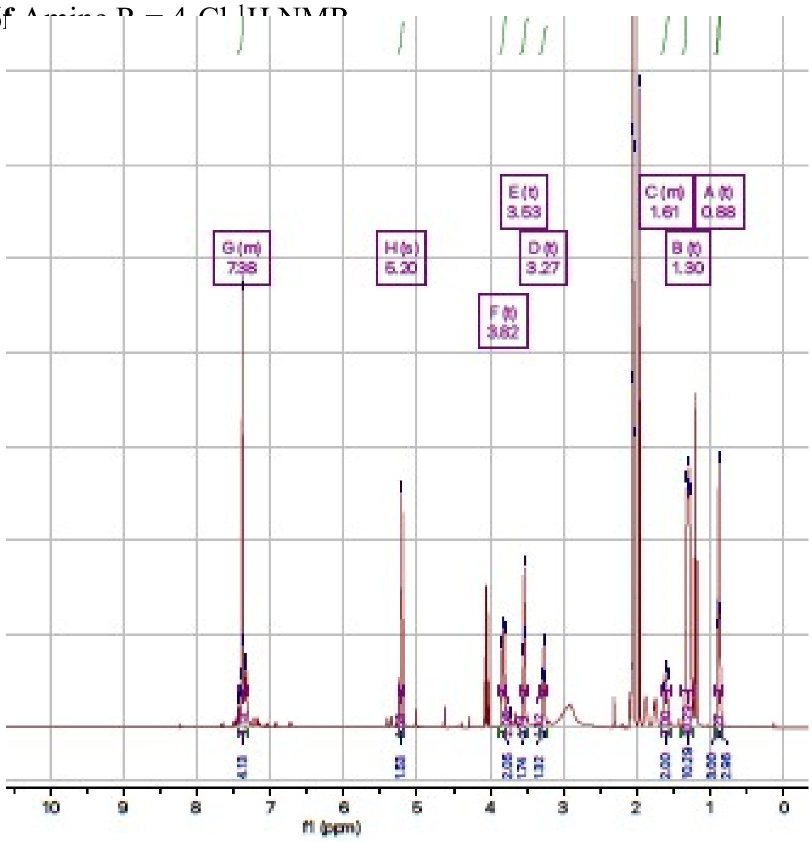

^{13}C NMR

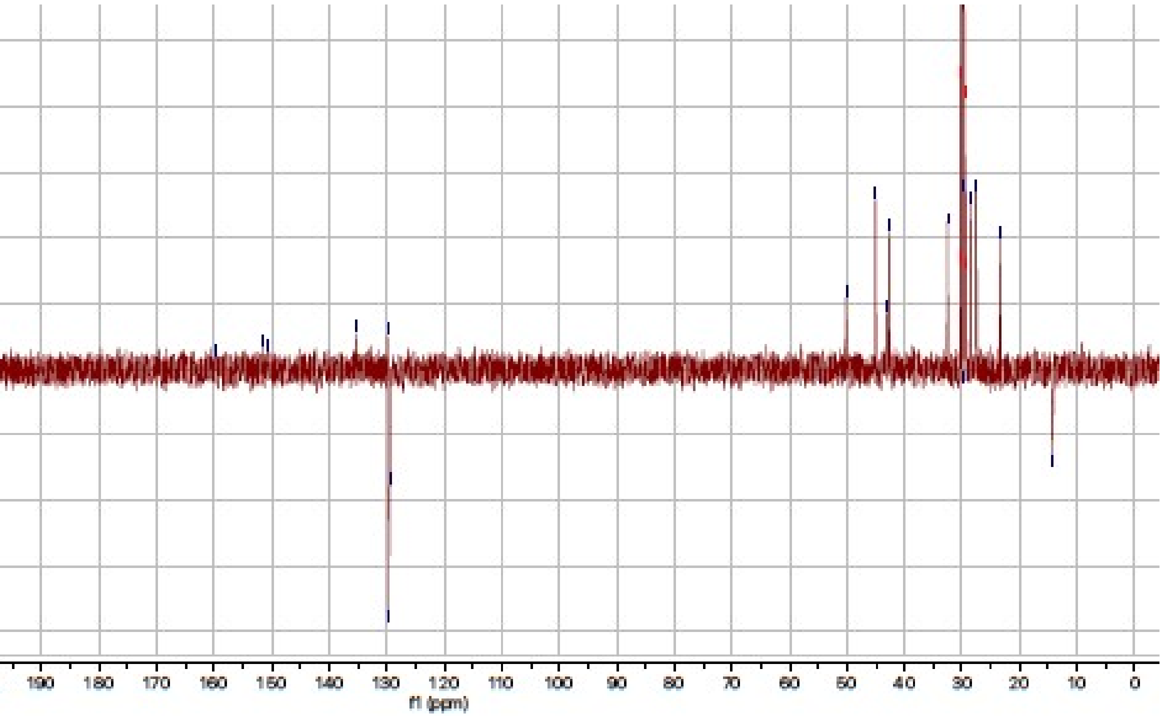
190
180
170
160
150
140
130
120
110
100
90
80
70
60
50
40
30
20
10
0
f1 (ppm)

7f Guanidine R = 4-Cl ^{1}H NMR

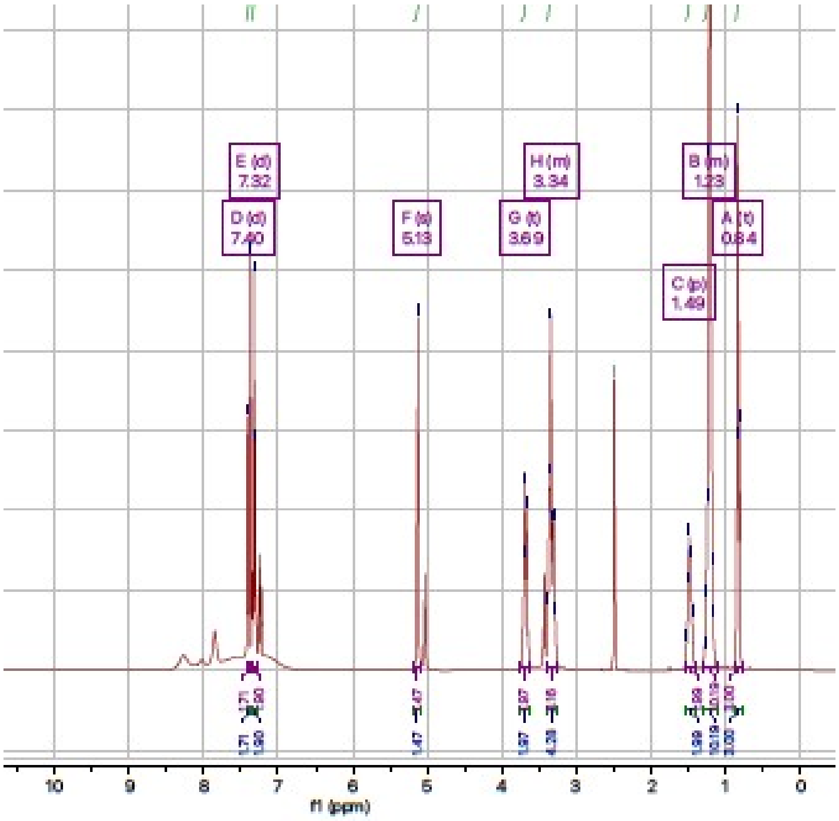

^{13}C NMR

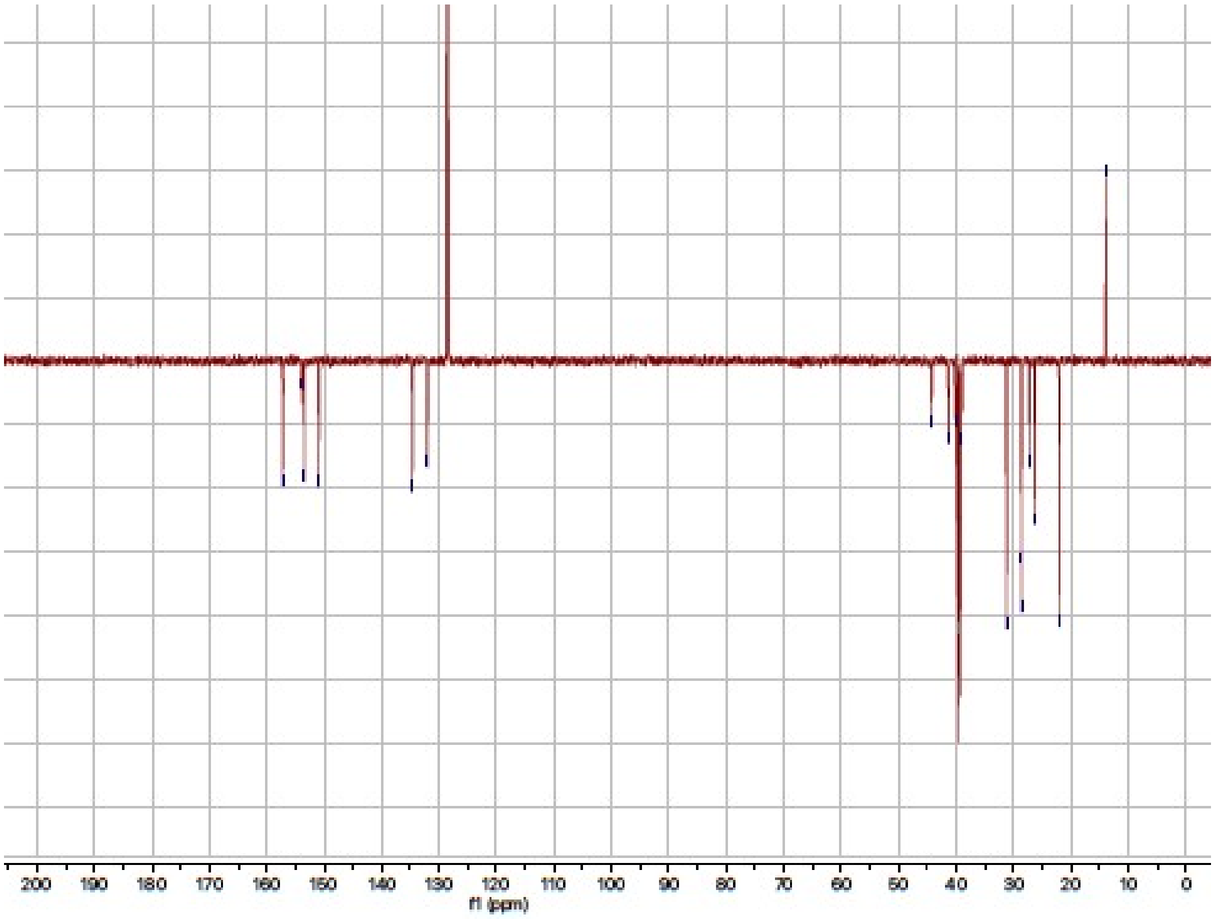

6g Amine R = 4-Br ^{1}H NMR

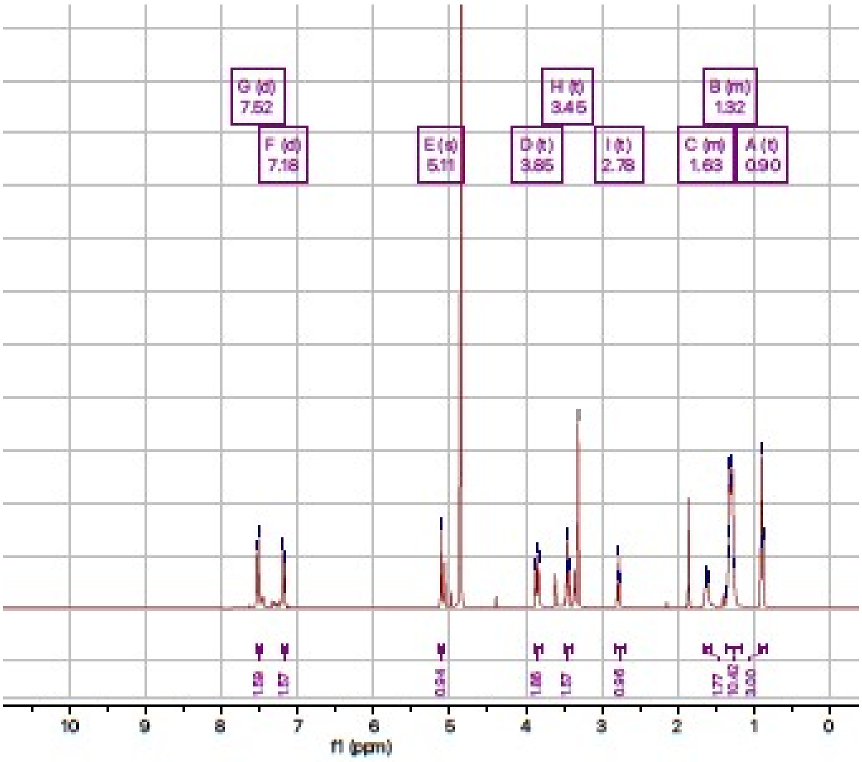
G (d)
7.52
F (d)
7.18
E (s)
5.11
D (t)
3.85
H (t)
3.45
I (t)
2.78
B (m)
1.32
C (m)
1.63
A (t)
0.90
1.59
1.57
0.94
1.86
1.57
0.96
1.77
10.40
3.00
10
9
8
7
6
5
4
3
2
1
0
f1 (ppm)

^{13}C NMR

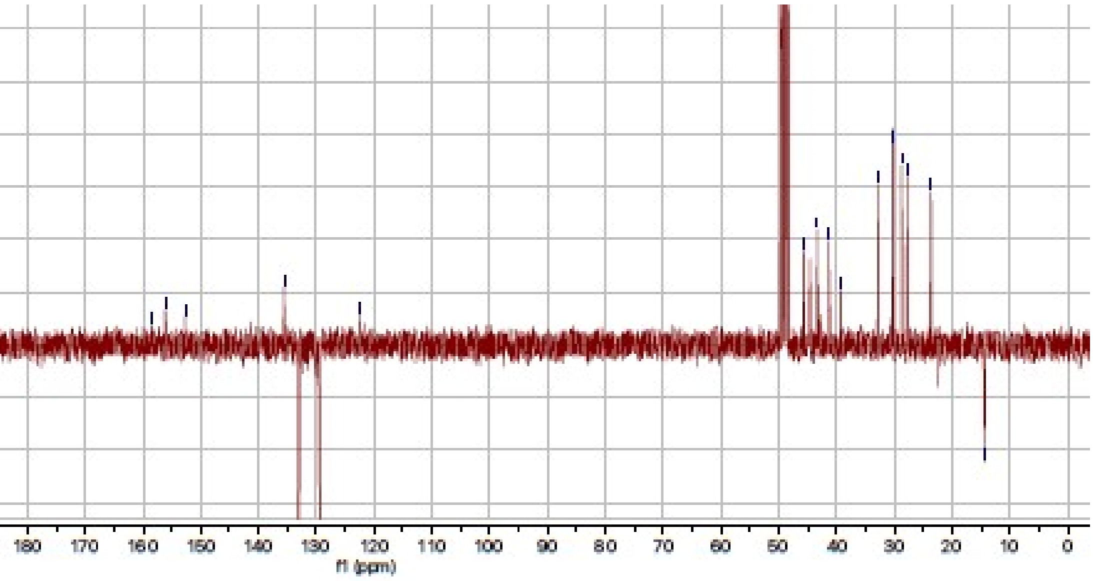
180
170
160
150
140
130
120
110
100
90
80
70
60
50
40
30
20
10
0
f1 (ppm)

7g Guanidine R = 4-Br ^{1}H NMR

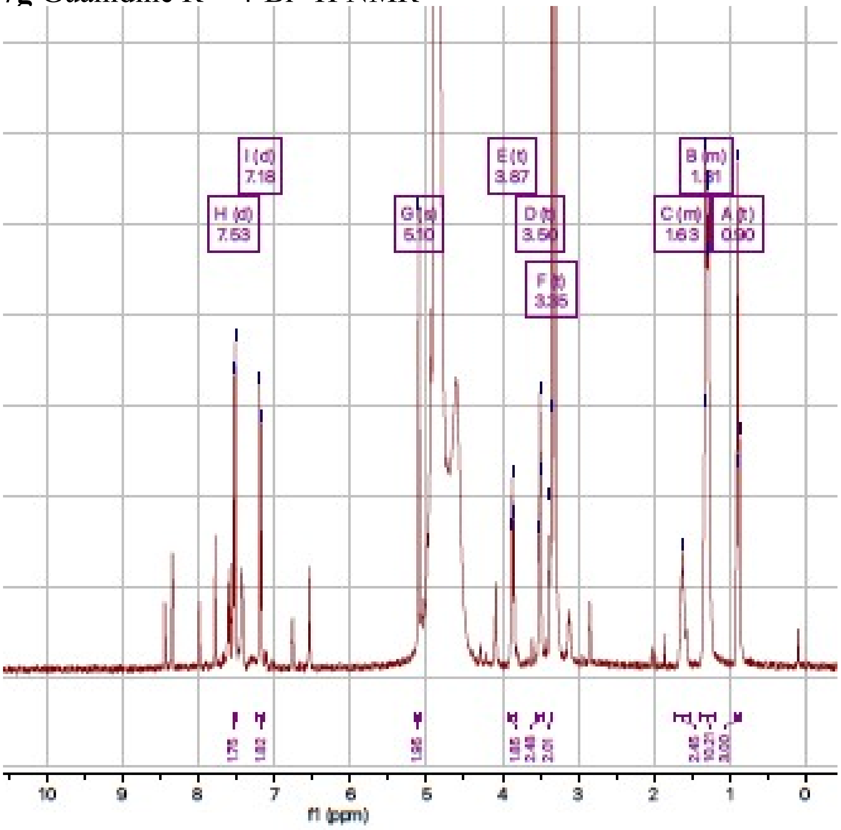

^{13}C NMR

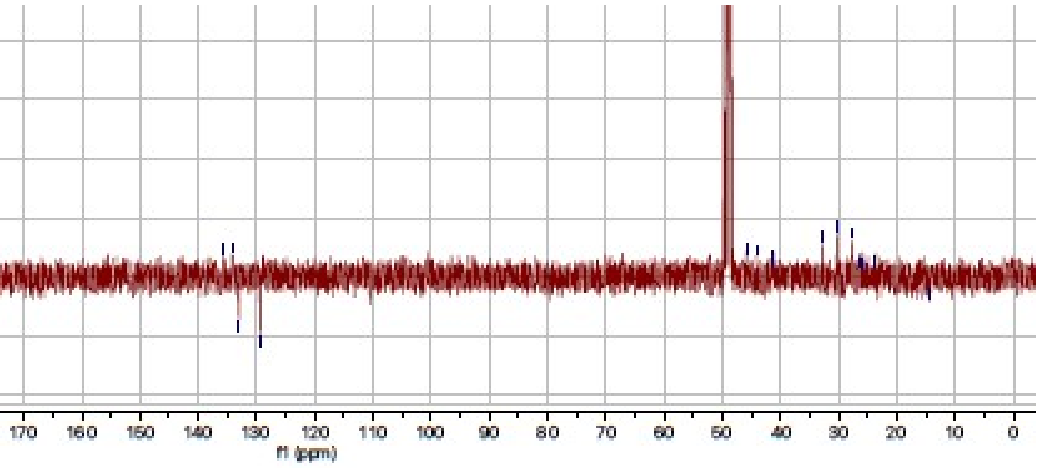
170 160 150 140 130 120 110 100 90 80 70 60 50 40 30 20 10 0
f1 (ppm)

6h Amine R = 4-NO_2 ^{1}H NMR

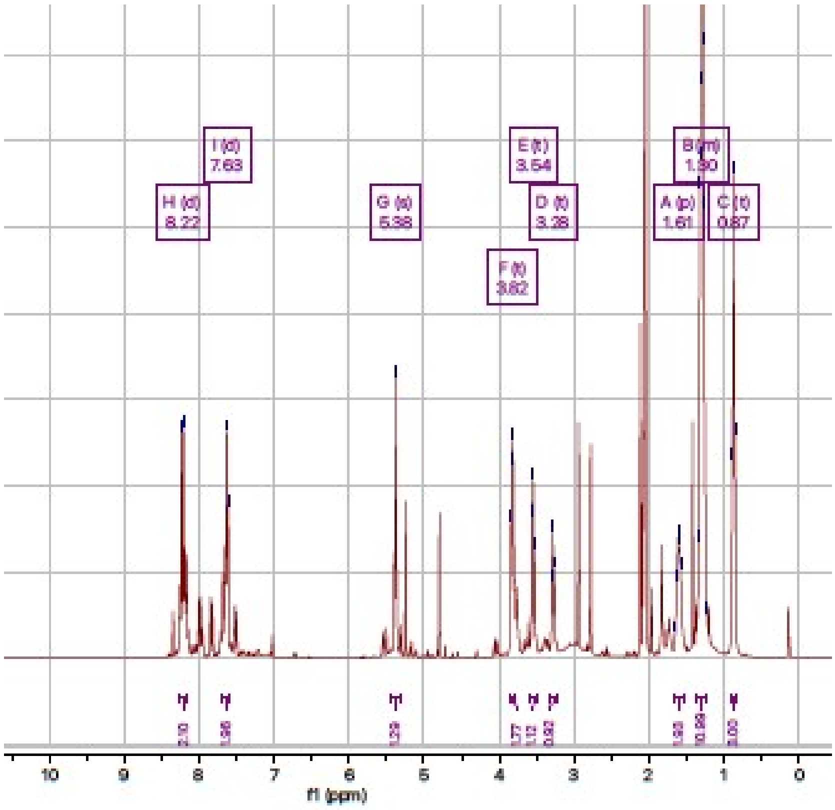

^{13}C NMR

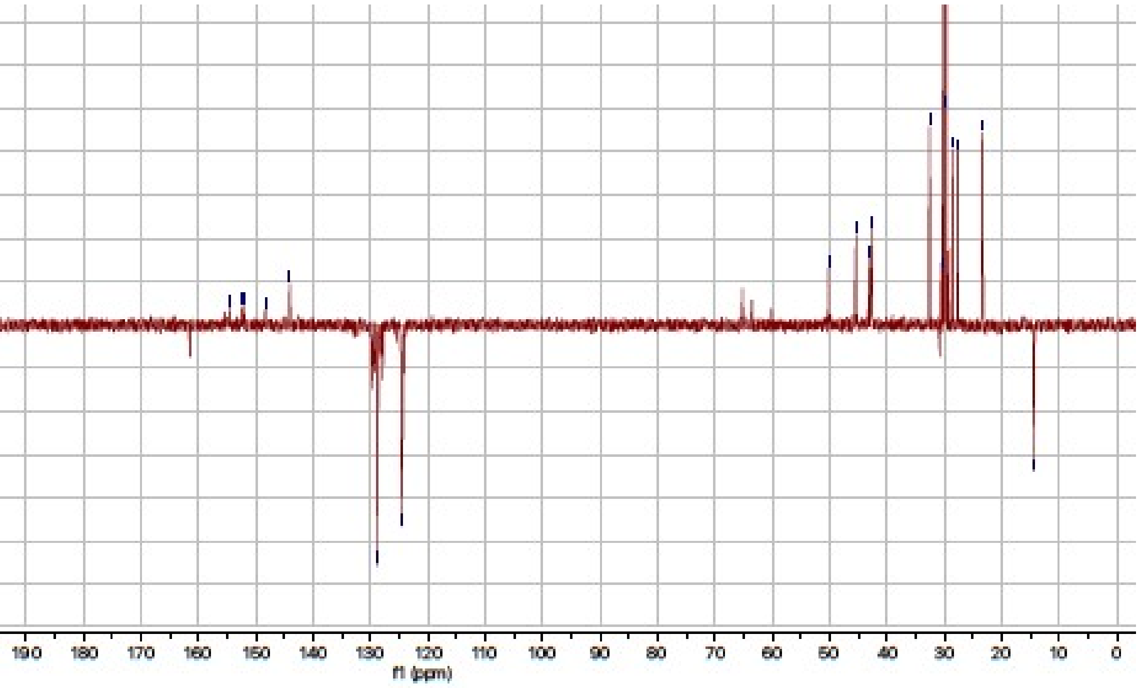
190 180 170 160 150 140 130 120 110 100 90 80 70 60 50 40 30 20 10 0
f1 (ppm)

7h Guanidine R = 4-NO_2 ^{1}H NMR

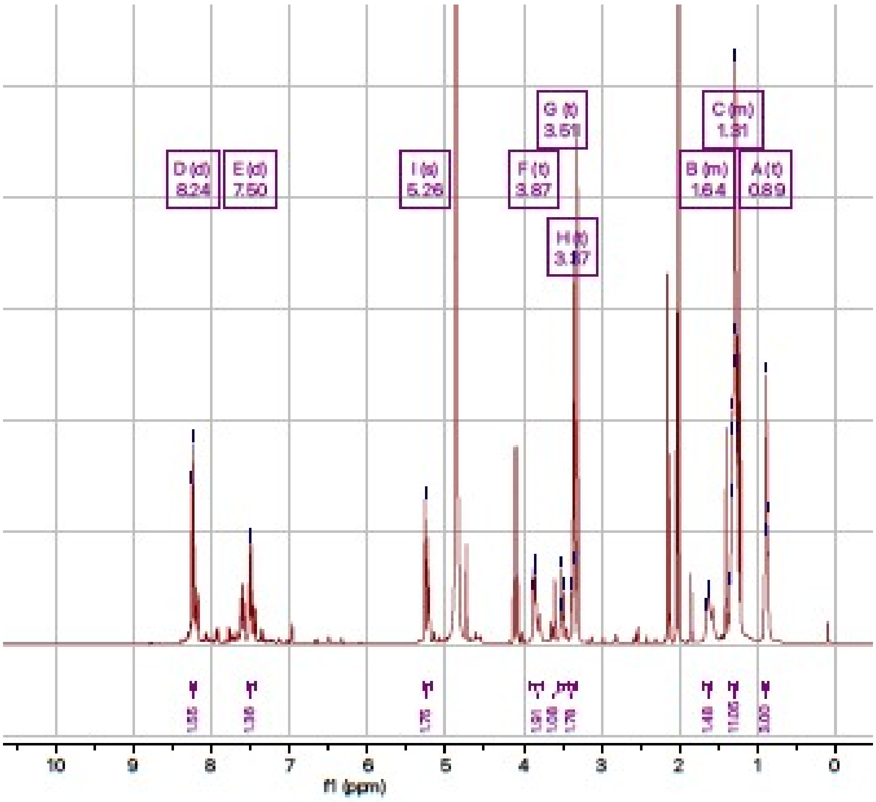

^{13}C NMR

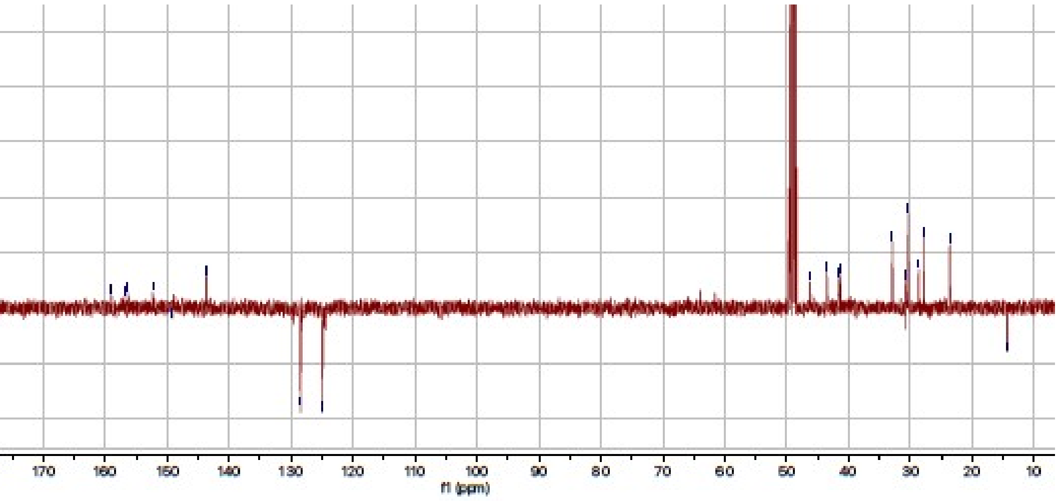
170
160
150
140
130
120
110
100
90
80
70
60
50
40
30
20
10
f1 (ppm)

6j Amine R = 4-CF_3 ^1H NMR

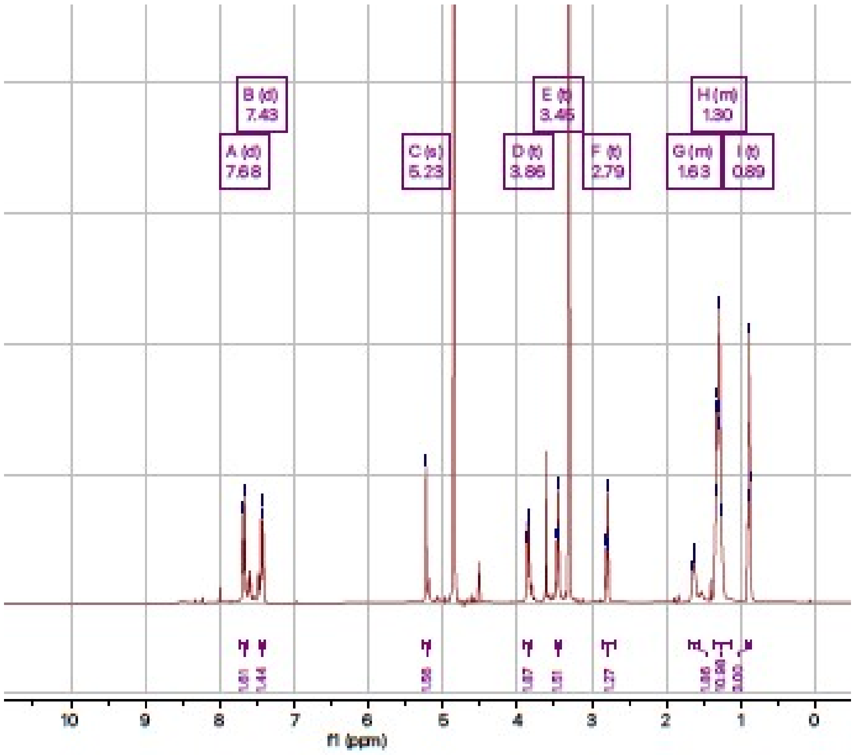

^{13}C NMR

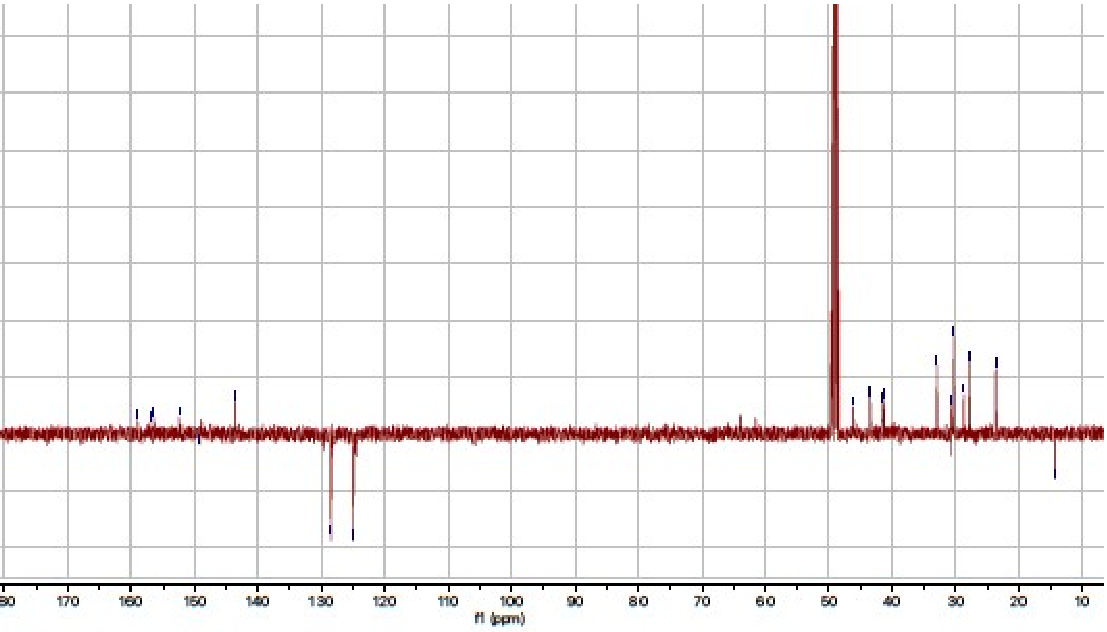

170
160
150
140
130
120
110
100
90
80
70
60
50
40
30
20
10
f1 (ppm)

7j Guanidine R = 4-CF_3 ^1H NMR

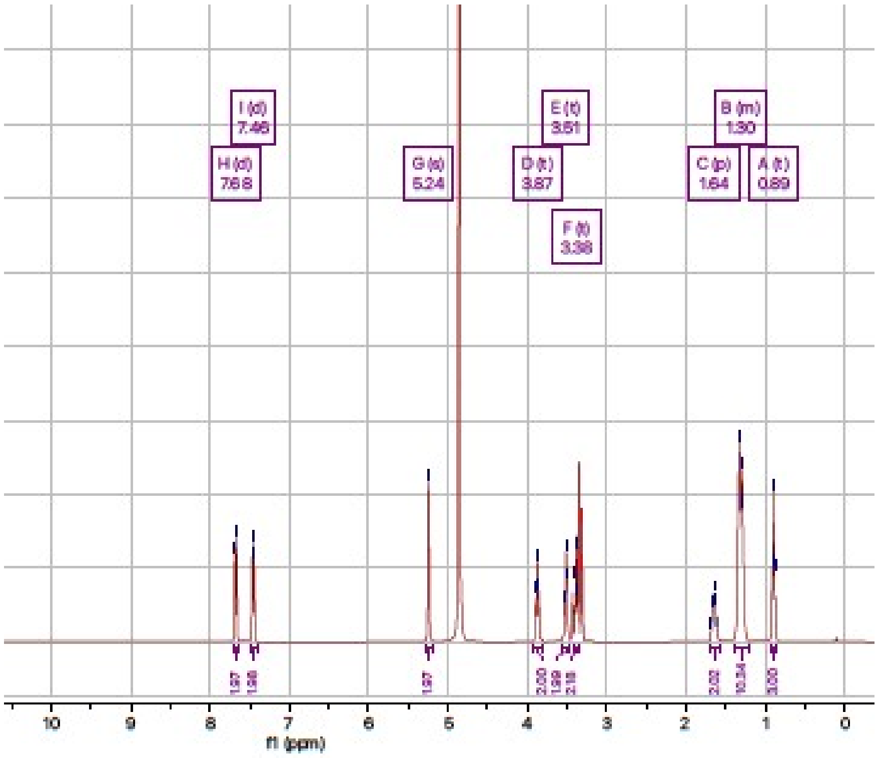

^{13}C NMR

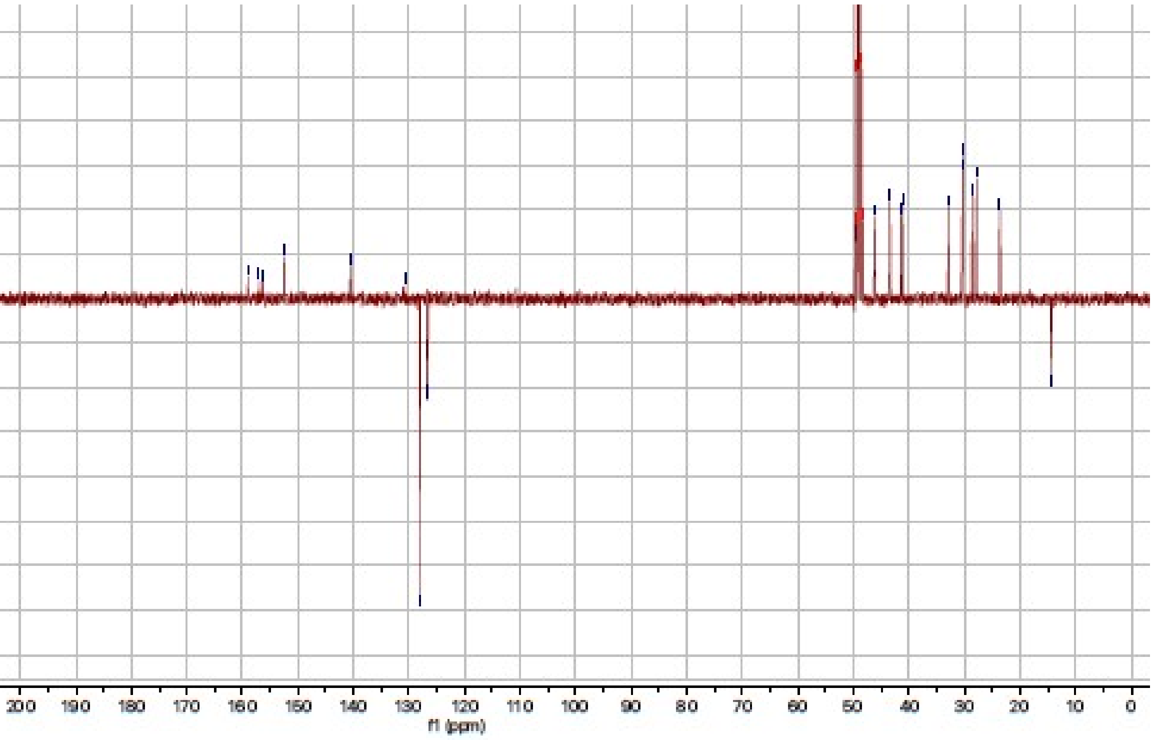
200
190
180
170
160
150
140
130
120
110
100
90
80
70
60
50
40
30
20
10
0
f1 (ppm)

8a N-methyl analogue R = H ^{1}H NMR

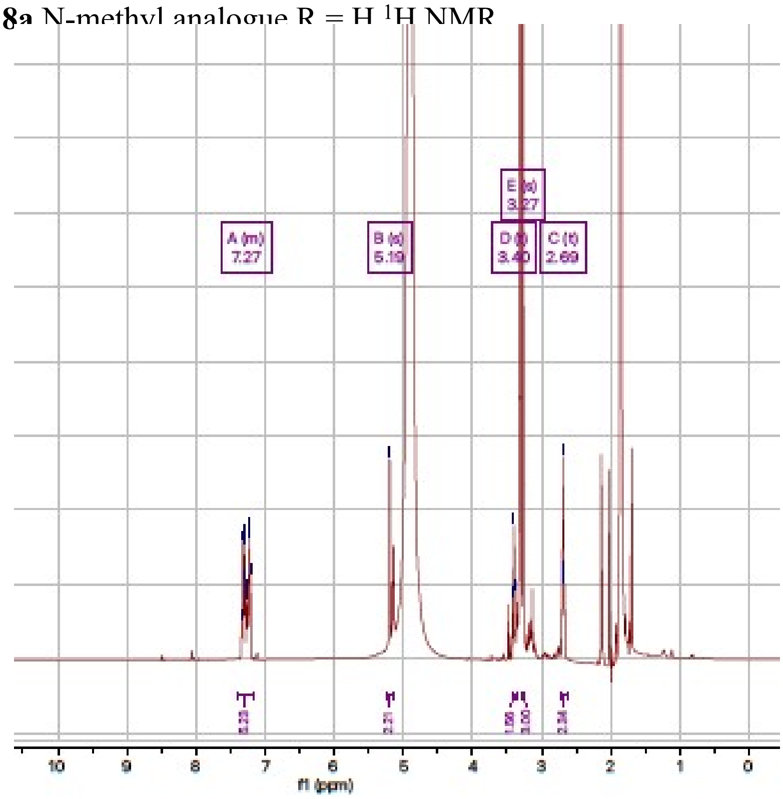

^{13}C NMR

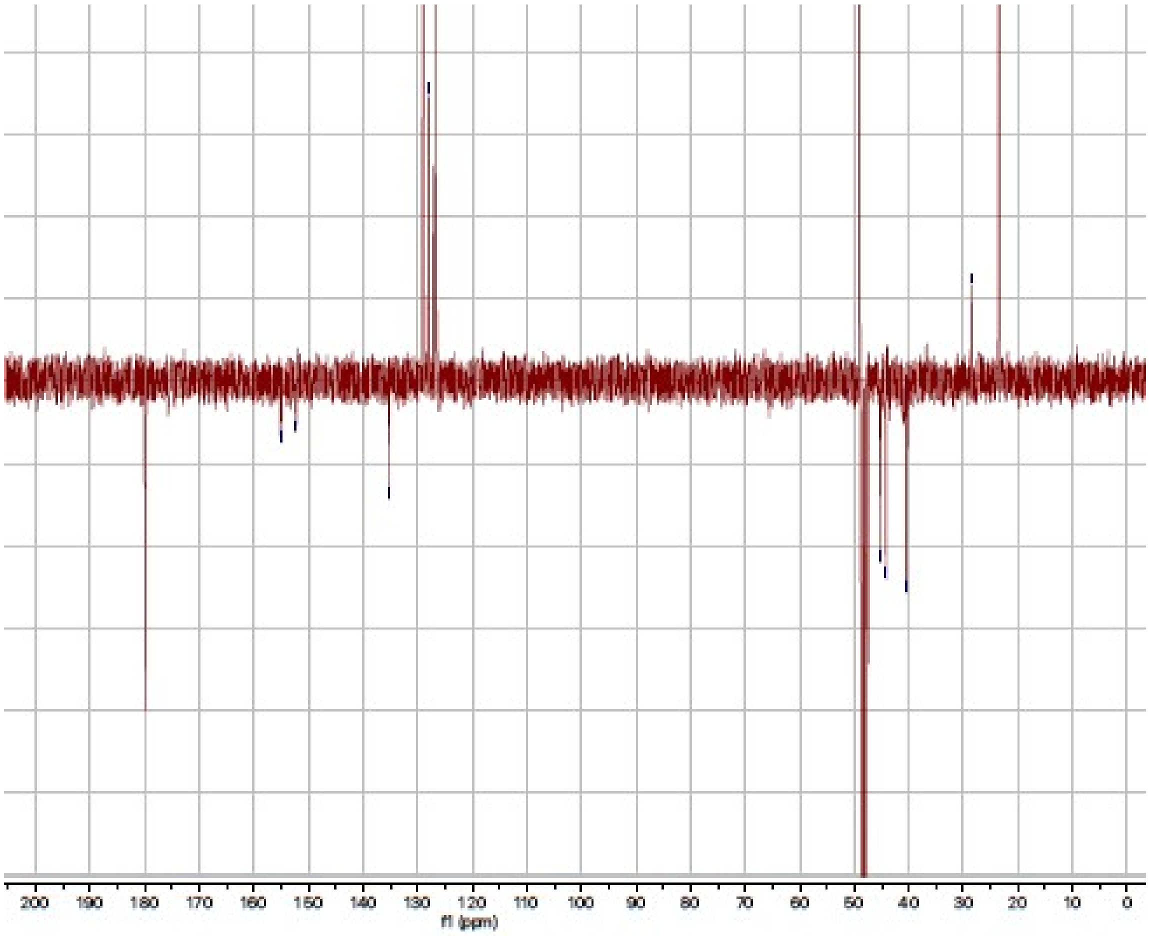

8b N-methyl analogue R = C(=NH)NH_2 ^{1}H NMR

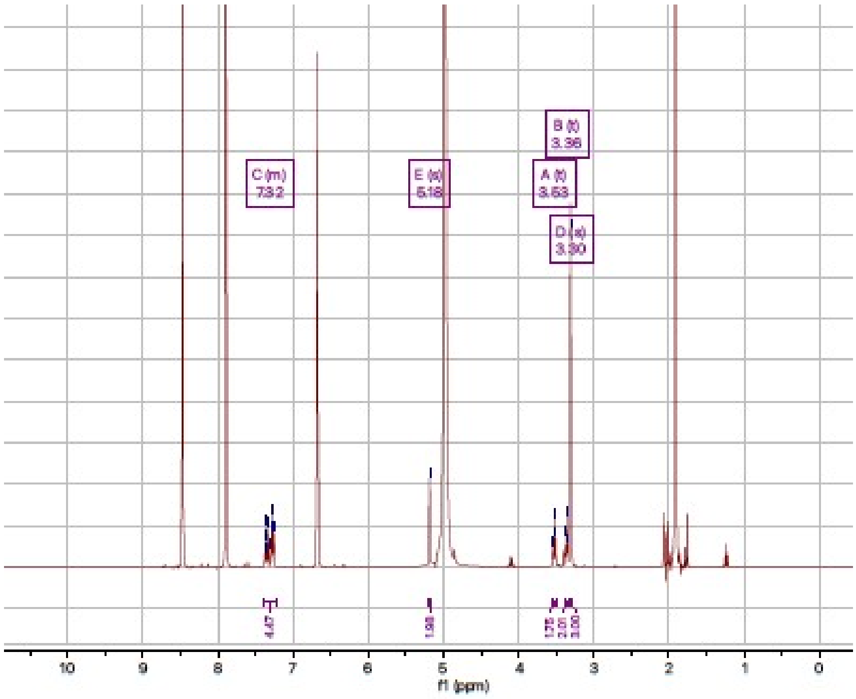

C (m)
7.32
E (s)
5.18
B (t)
3.36
A (t)
3.53
D (s)
3.30
4.47
1.98
1.25
2.01
3.00
10
9
8
7
6
5
4
3
2
1
0
f1 (ppm)

^{13}C NMR

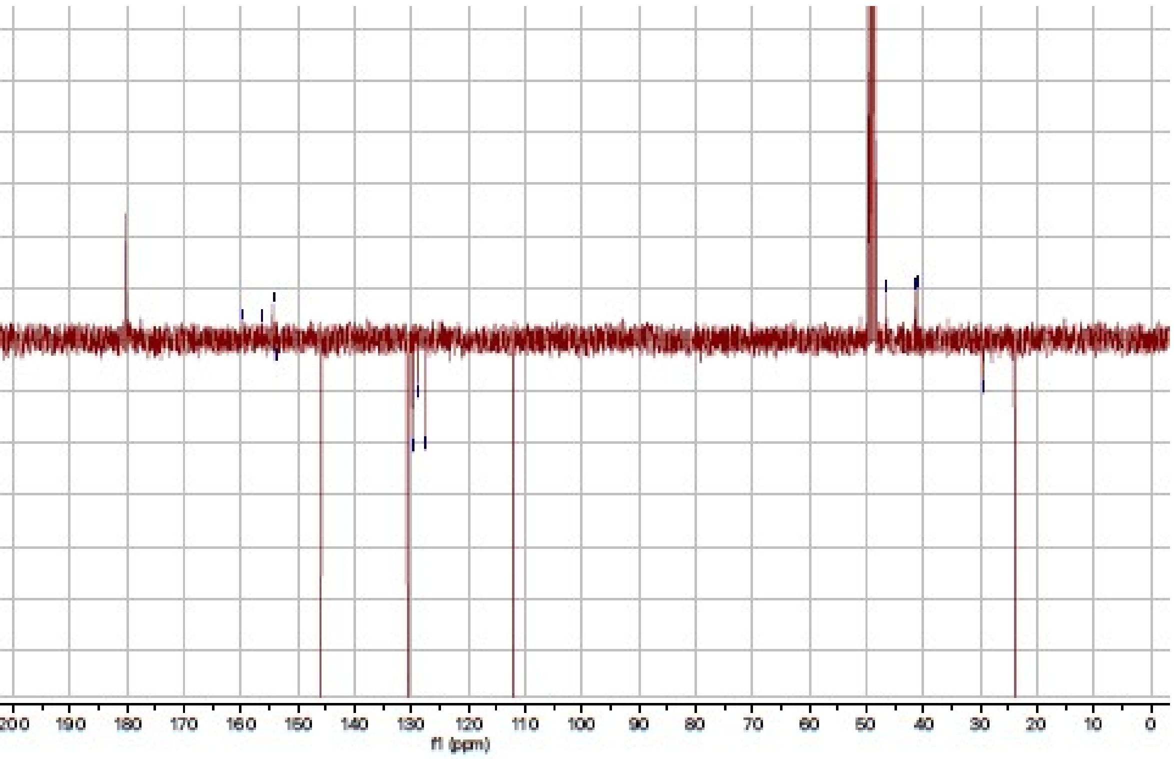
f1 (ppm)

9a Amine analogue lacking benzyl ^{1}H NMR

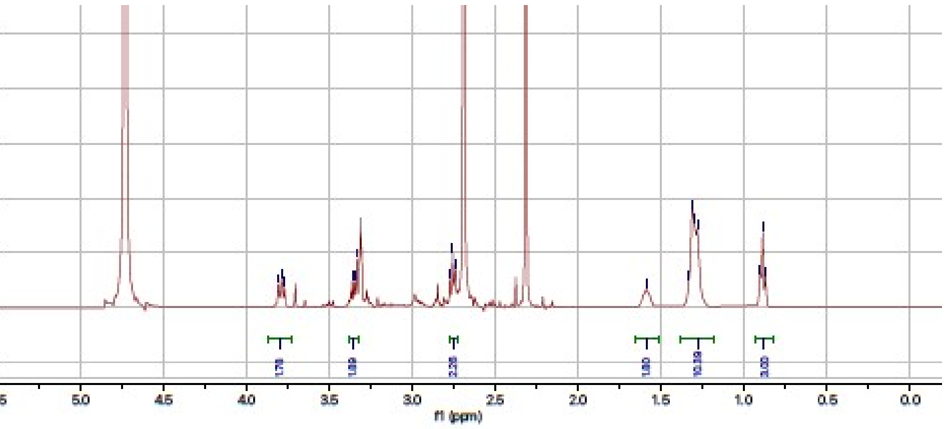

^{13}C NMR

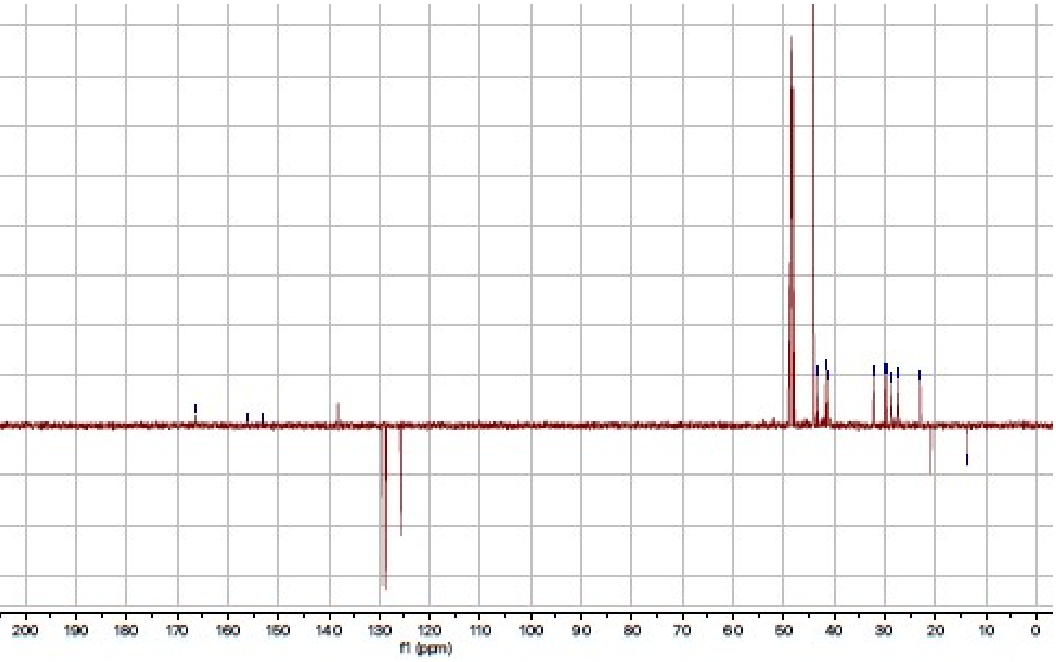

9b Guanidine analogue lacking benzyl ^{1}H NMR

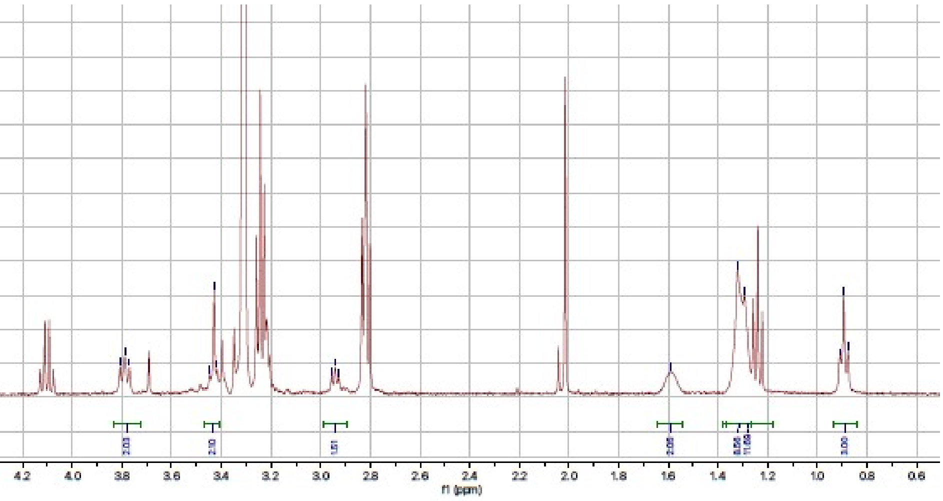

2.03
2.10
1.51
2.05
8.56
11.69
3.00
4.2
4.0
3.8
3.6
3.4
3.2
3.0
2.8
2.6
2.4
2.2
2.0
1.8
1.6
1.4
1.2
1.0
0.8
0.6
f1 (ppm)

10 N-ethyl analogue ^{1}H NMR

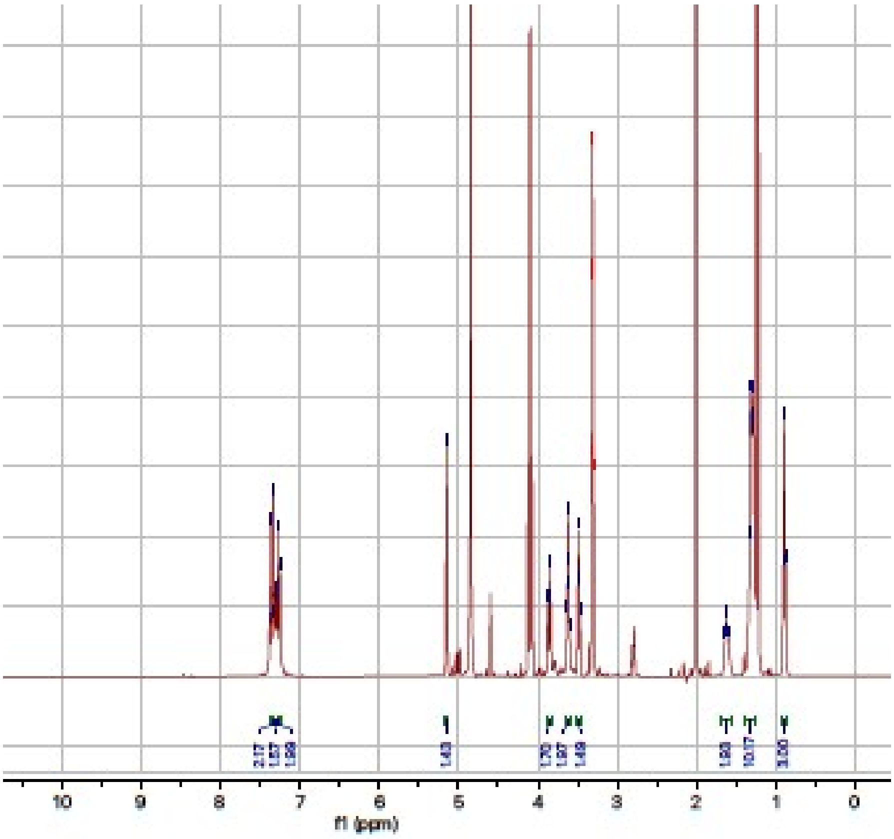

^{13}C NMR

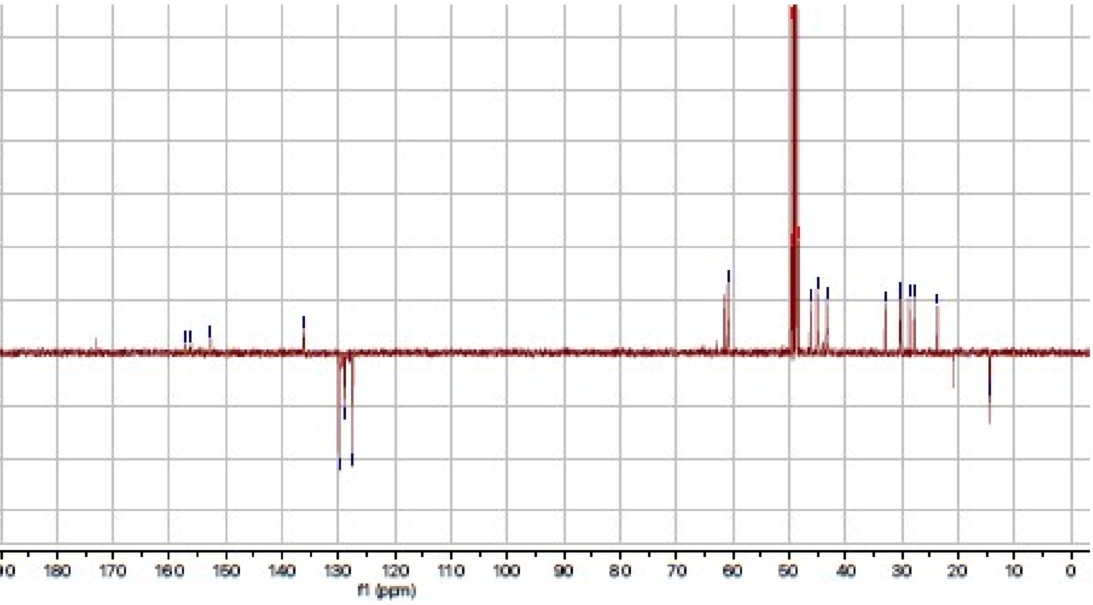

f1 (ppm)

11 N-hydroxyethyl analogue ^{1}H NMR

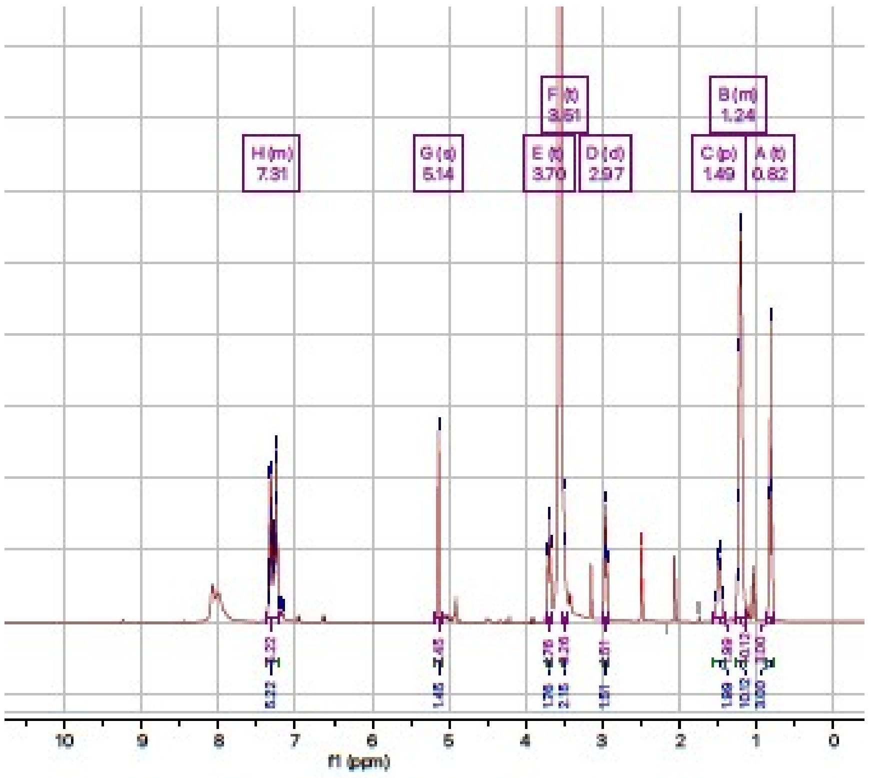

^{13}C NMR

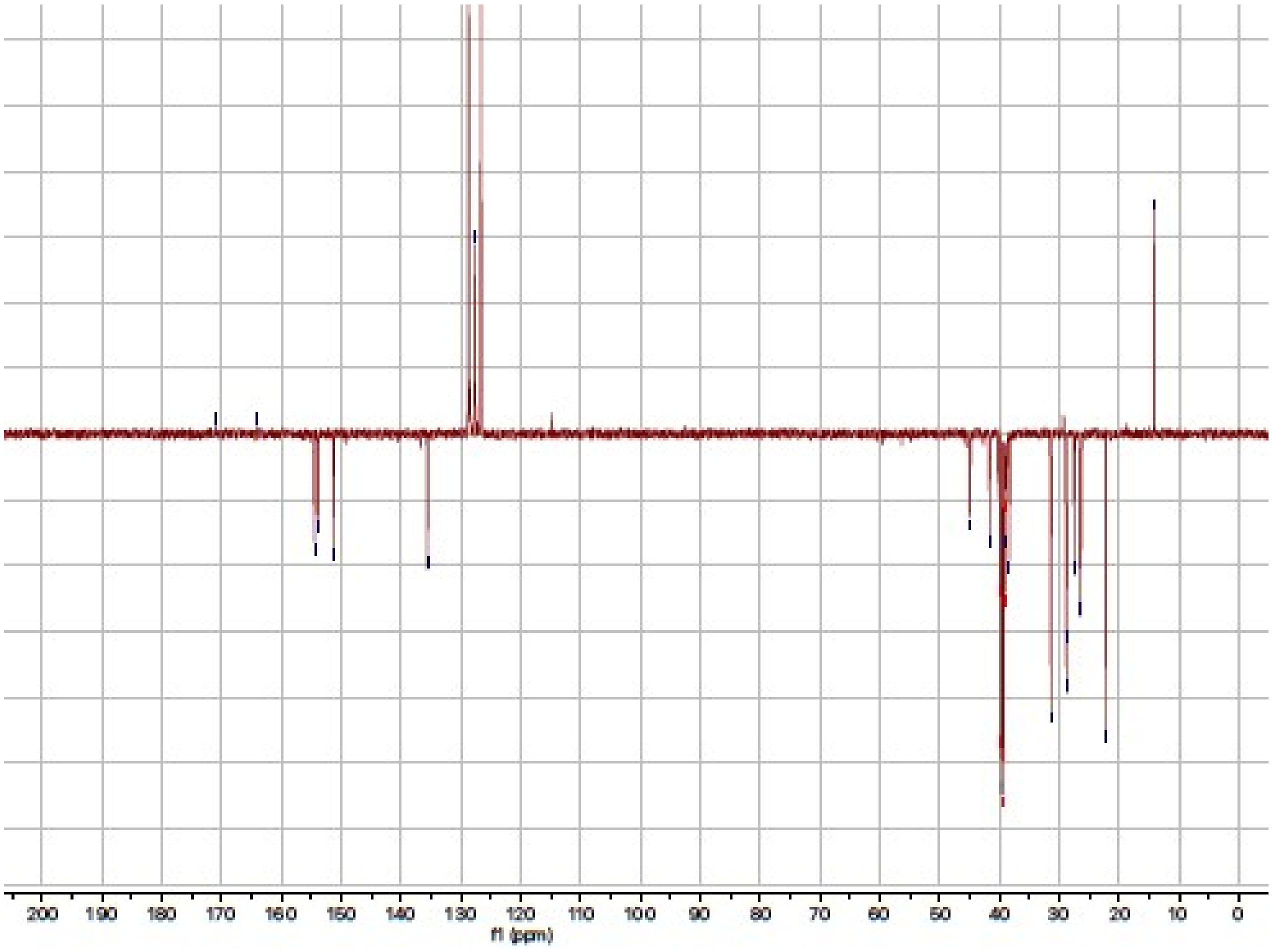
200 190 180 170 160 150 140 130 120 110 100 90 80 70 60 50 40 30 20 10 0
f1 (ppm)

12 Ur

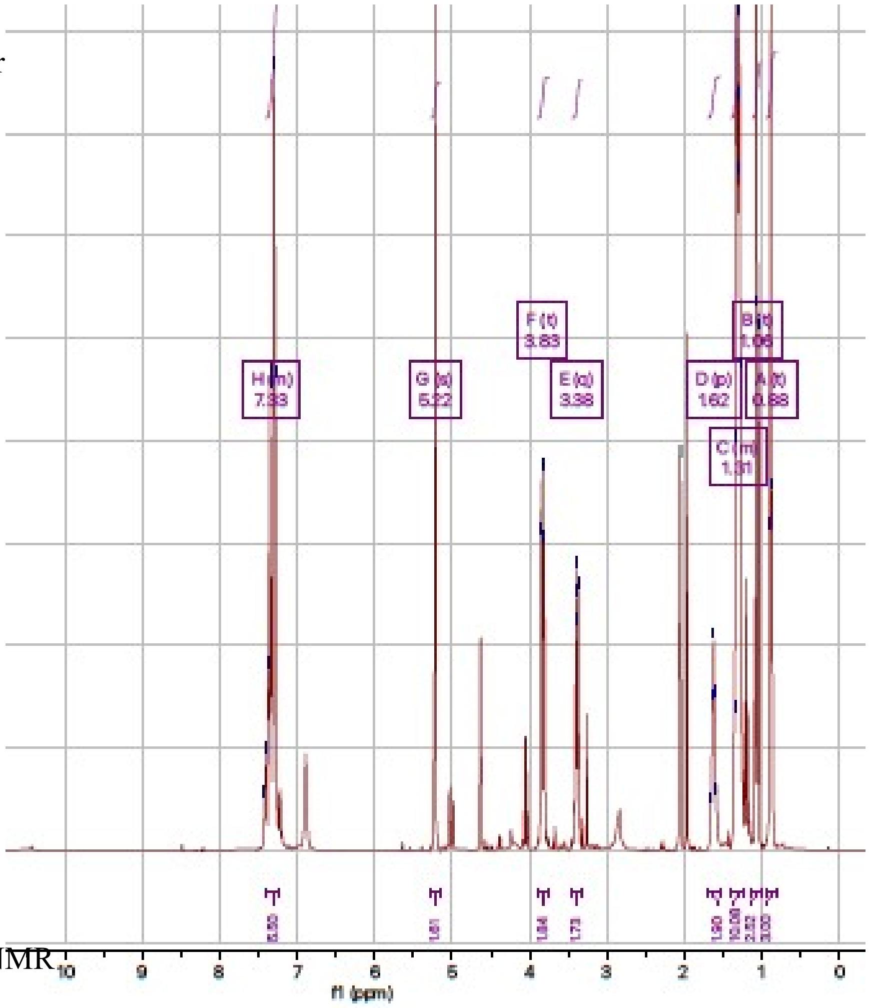

^{13}C NMR

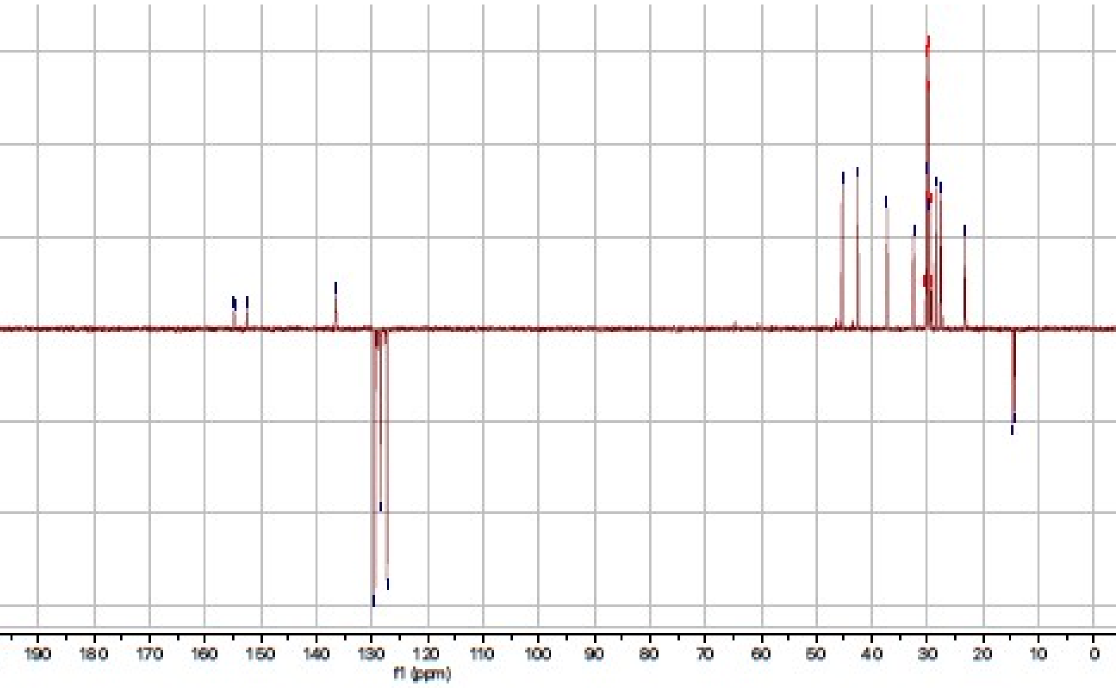
190 180 170 160 150 140 130 120 110 100 90 80 70 60 50 40 30 20 10 0
f1 (ppm)

13 Bis-aryl analogue ^{1}H NMR

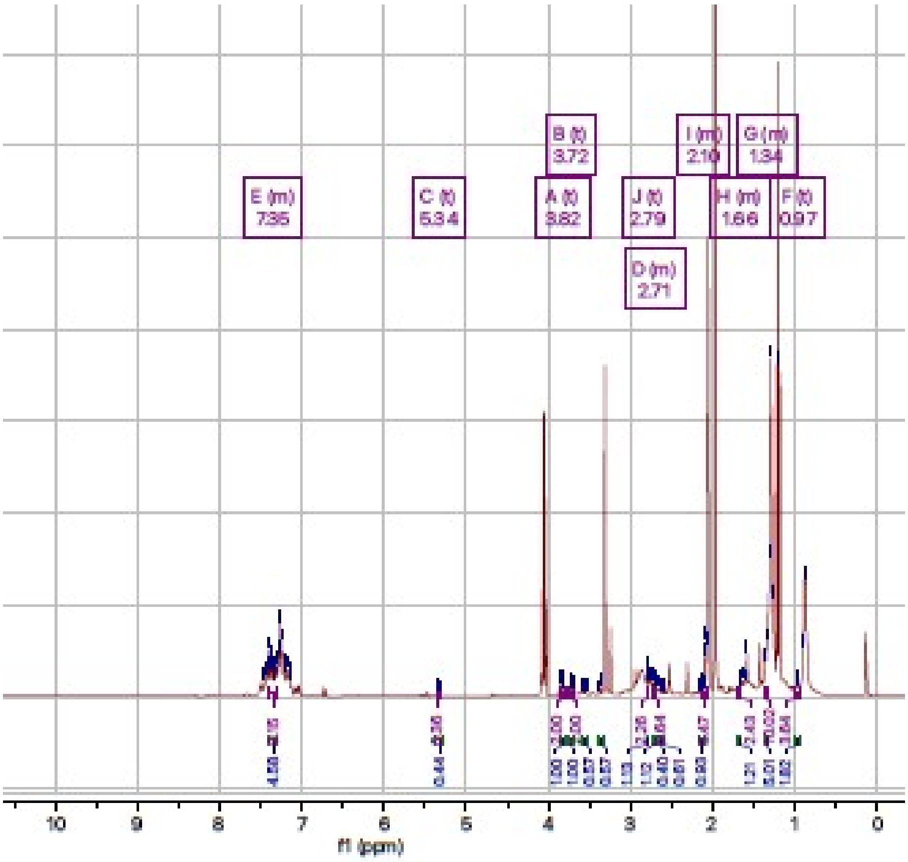

^{13}C NMR

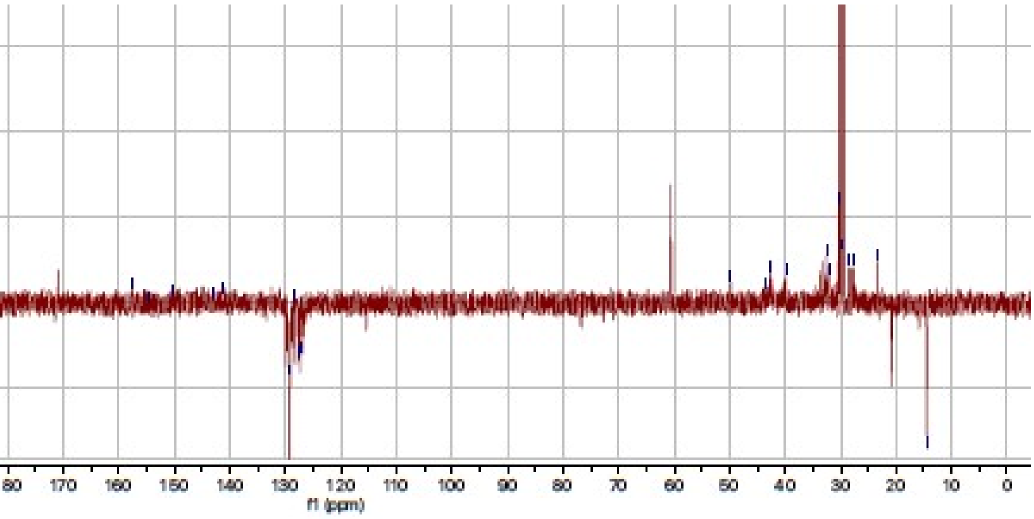
80 170 160 150 140 130 120 110 100 90 80 70 60 50 40 30 20 10 0
f1 (ppm)

14a Triazine amine analogue ^{1}H NMR

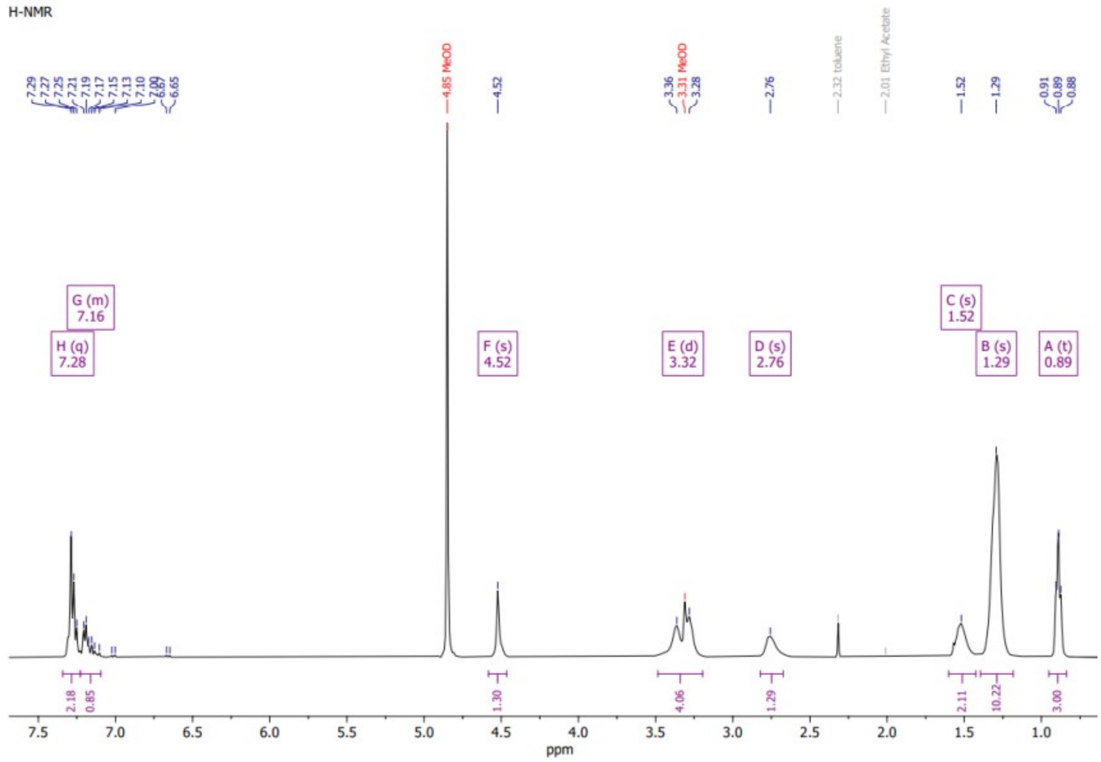

14b Triazine analogue guanidine ^{1}H NMR

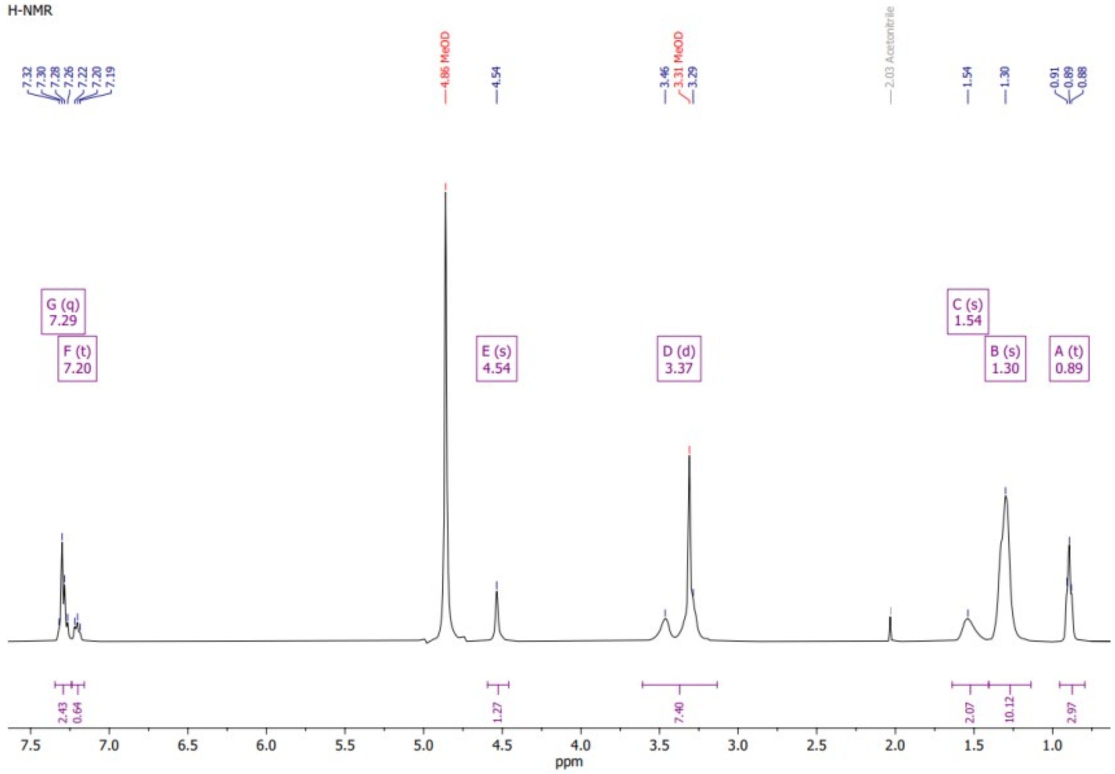

Supplement: MD-016-D4MD00937A-s001 [file MD-016-D4MD00937A-s001.pdf]
